# Factors impacting survival after transarterial radioembolization in patients with hepatocellular carcinoma: Results from the prospective CIRT study

**DOI:** 10.1016/j.jhepr.2022.100633

**Published:** 2022-11-25

**Authors:** Frank Kolligs, Dirk Arnold, Rita Golfieri, Maciej Pech, Bora Peynircioglu, Thomas Pfammatter, Maxime Ronot, Bruno Sangro, Niklaus Schaefer, Geert Maleux, Graham Munneke, Helena Pereira, Bleranda Zeka, Niels de Jong, Thomas Helmberger, CIRT Principal Investigators, Thomas Albrecht, Olivier D’Archambeau, Tugsan Balli, Sadik Bilgic, Allan Bloom, Roberto Cioni, Roman Fischbach, Patrick Flamen, Laurent Gerard, Gerd Grözinger, Marcus Katoh, Michael Koehler, Jan Robert Kröger, Christiane Kuhl, Franco Orsi, Murat Özgün, Peter Reimer, Maxime Ronot, Axel Schmid, Alessandro Vit

**Affiliations:** 1 Department of Internal Medicine and Gastroenterology, Helios Klinikum Berlin-Buch, Berlin, Germany; 2 Oncology and Hematology, Asklepios Tumorzentrum Hamburg, AK Altona, Hamburg, Germany; 3 Department of Radiology, IRCCS Azienda Ospedaliero-Universitaria di Bologna, Bologna, Italy; 4 Department of Radiology and Nuclear Medicine, University of Magdeburg, Magdeburg, Germany; 5 Department of Radiology, School of Medicine, Hacettepe University, Sihhiye Campus, Ankara, Turkey; 6 Institute of Diagnostic and Interventional Radiology, Universitätsspital Zürich, Zürich, Switzerland; 7 Université Paris Cité, Paris & Service de Radiologie, APHP Nord, Hôpital Beaujon, Clichy, France; 8 Liver Unit and HPB Oncology Area, Clínica Universidad de Navarra and CIBEREHD, Pamplona, Spain; 9 Service de médecine nucléaire et imagerie moléculaire, CHUV, Centre Hospitalier Universitaire Vaudois, Lausanne, Switzerland; 10 Radiology, Universitair Ziekenhuis Leuven, Leuven, Belgium; 11 Interventional Oncology, University College London Hospitals NHS Foundation Trust, London, United Kingdom; 12 Assistance Publique-Hôpitaux de Paris, Hôpital Européen Georges-Pompidou, Unité de Recherche Clinique, Paris, France; 13 Clinical Research Department, Cardiovascular and Interventional Radiological Society of Europe, Vienna, Austria; 14 Department of Radiology, Neuroradiology and Minimal-Invasive Therapy, Klinikum Bogenhausen, Munich, Germany; 15 INSERM, Centre d'Investigation Clinique 1418 (CIC1418), Paris, France

**Keywords:** SIRT, observational, liver, radioembolization, dosimetry, registry, ALBI, albumin-bilirubin, BCLC, Barcelona Clinic Liver Cancer, BSA, body surface area, CIRSE, Cardiovascular and Interventional Radiological Society of Europe, CIRT, CIRSE Registry for SIR-Spheres Therapy, ECOG, Eastern Cooperative Oncology Group, HCC, hepatocellular carcinoma, hPFS, hepatic progression-free survival, HR, hazard ratio, INR, international normalized ratio, IPTW, inverse probability of treatment weighting, mBSA, modified body surface area, OS, overall survival, PFS, progression-free survival, PVT, portal vein thrombosis, REILD, radioembolization-induced liver disease, TACE, transcatheter arterial chemoembolization, TARE, transarterial radioembolization, Y90, Yttrium-90

## Abstract

**Background & Aims:**

Transarterial radioembolization (TARE) with Yttrium-90 resin microspheres is an established treatment option for patients with hepatocellular carcinoma (HCC). However, optimising treatment application and patient selection remains challenging. We report here on the effectiveness, safety and prognostic factors, including dosing methods, associated with TARE for HCC in the prospective observational CIRT study.

**Methods:**

We analysed 422 patients with HCC enrolled between Jan 2015 and Dec 2017, with follow-up visits every 3 months for up to 24 months after first TARE. Patient characteristics and treatment-related data were collected at baseline; adverse events and time-to-event data (overall survival [OS], progression-free survival [PFS] and hepatic PFS) were collected at every 3-month follow-up visit. We used the multivariable Cox proportional hazard model and propensity score matching to identify independent prognostic factors for effectiveness outcomes.

**Results:**

The median OS was 16.5 months, the median PFS was 6.1 months, and the median hepatic PFS was 6.7 months. Partition model dosimetry resulted in improved OS compared to body surface area calculations on multivariable analysis (hazard ratio 0.65; 95% CI 0.46-0.92; *p* = 0.0144), which was confirmed in the exact matching propensity score analysis (hazard ratio 0.56; 95% CI 0.35-0.89; *p =* 0.0136). Other independent prognostic factors for OS were ECOG-performance status >0 (*p =* 0.0018), presence of ascites (*p =* 0.0152), right-sided tumours (*p =* 0.0002), the presence of portal vein thrombosis (*p =* 0.0378) and main portal vein thrombosis (*p =* 0.0028), ALBI grade 2 (*p =* 0.0043) and 3 (*p =* 0.0014). Adverse events were recorded in 36.7% of patients, with 9.7% of patients experiencing grade 3 or higher adverse events.

**Conclusions:**

This large prospective observational dataset shows that TARE is an effective and safe treatment in patients with HCC. Using partition model dosimetry was associated with a significant improvement in survival outcomes.

**Impact and implications:**

Transarterial radioembolization (TARE) is a form of localised radiation therapy and is a potential treatment option for primary liver cancer. We observed how TARE was used in real-life clinical practice in various European countries and if any factors predict how well the treatment performs. We found that when a more complex but personalised method to calculate the applied radiation activity was used, the patient responded better than when a more generic method was used. Furthermore, we identified that general patient health, ascites and liver function can predict outcomes after TARE.

**Clinical trial number:**

NCT02305459.

## Highlights

• CIRT is a large, European-wide multicentre prospective observational study.
• Partition model dosimetry is associated with improved overall survival compared to the standard body surface area model.
• ALBI grades, ECOG status, ascites, portal vein thrombosis and tumour location predict survival outcomes after TARE.
• TARE is a safe treatment associated with a low occurence of adverse events.

## Graphical abstract

**Figure:**
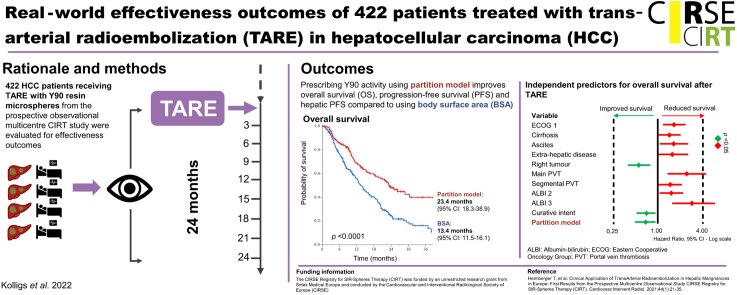

## Introduction

Hepatocellular carcinoma (HCC) is the most frequent primary liver cancer and represents the third most common cause of cancer-related deaths worldwide.^1^^,^^2^ The most critical risk factor for the development of HCC is cirrhosis. Overall, the prognosis of HCC is poor, with a life expectancy of 6-38 months, depending on the Barcelona Clinic Liver Cancer (BCLC) stage.^3^ Only a minority of patients are eligible for curative-intent treatments, including surgical resection, liver transplantation, and ablative therapies.[4], [5], [6], [7], [8], [9] In intermediate stages, transcatheter arterial chemoembolization (TACE) is standard of care; systemic treatments such as sorafenib, lenvatinib, and a combination of atezolizumab and bevacizumab have been approved for the first-line medical treatment of advanced and metastatic HCC based on convincing phase III trials.[10], [11], [12], [13]

Guidelines for the treatment of HCC also propose transarterial radioembolization (TARE, also known as selective internal radiation therapy [SIRT]) as an optional treatment modality for patients with liver dominant disease not eligible for surgical or ablative therapies, or who experienced no response, significant side effects or intolerance when treated with systemic therapies.^4^^,^[6], [7], [8], [9] TARE is an interventional therapeutic procedure that involves the targeted delivery of high doses of radiation to liver tumours via the hepatic artery. Several studies have shown that TARE has a favourable safety profile and displays promising results in terms of local tumour control in patients with unresectable HCC limited to the liver in the intermediate and advanced stages.[14], [15], [16], [17], [18] Despite this, recent randomised controlled trials on TARE in HCC showed that compared to sorafenib alone, TARE or TARE plus sorafenib as a first-line treatment option for patients with unresectable HCC did not improve overall survival (OS) or progression-free survival (PFS) in these patient cohorts.^19^^,^^20^ While questions have been raised regarding discrepancies in patient inclusion and site experience in administering TARE, which may have influenced outcomes,^21^ recent research into dosimetry methods suggests that improving dose calculation and delivery could improve survival outcomes.^22^ The prospective randomised DOSISPHERE-01 trial showed significantly better overall survival results with a personalised dosimetry model than the standard dose calculation model using glass microspheres in patients with unresectable, locally advanced HCC.^23^^,^^24^ This suggests that further optimising selection of patients, treatment application and the dosimetry models may improve survival outcomes of patients with HCC.

The Cardiovascular and Interventional Radiological Society of Europe (CIRSE) initiated a European-wide observational study on the clinical application and outcomes of TARE with Y90 resin microspheres (SIR-Spheres® Y-90 resin microspheres, Sirtex Medical Pty Limited; St. Leonards, NSW, Australia). The study (NCT02305459) was open to all indications and recruited one of the largest cohorts on TARE in liver malignancies to date.^25^ The objective of the current subgroup analysis was to investigate factors influencing survival in patients with HCC treated with TARE, including the effect that methods to calculate the prescribed dose have on effectiveness outcomes. The primary endpoint was OS, while secondary endpoints were PFS, PFS in the liver only (hepatic PFS [hPFS]), safety, and identification of potential prognostic survival factors, including an evaluation of the impact of methods to calculate the prescribed activity on survival outcomes.

## Patients and methods

### Study design

We analysed 422 patients with HCC collected in the CIRSE Registry for SIR-Spheres Therapy (CIRT) study. CIRT is a prospective, single device, multi-centre observational study of patients with primary and metastatic hepatic malignancies treated with TARE using Y90 resin microspheres as the standard of care. The CIRT methodology was published by Helmberger *et al.*^26^ Sites were invited to participate if they had at least 40 TARE cases and 10 cases in 12 months prior to invitation. In total, 27 participating sites in eight countries were identified and enrolled from April 2014 until April 2017, of which 25 sites included patients with HCC.^25^

Data was collected using a customised electronic data capturing system and electronic case report form that was developed by ConexSys Inc (Lincoln, RI, United States) and hosted on a local secure server in Vienna, Austria maintained by ITEA (Vienna, Austria). Statistical analyses were performed in SAS 9.4 (SAS Institute, Cary, NC, USA) and RStudio under R4.0.0 (R Foundation, Vienna, Austria, Supplementary CTAT Table).

### Patient selection

Patients included in the analysis were adults diagnosed with HCC and scheduled to receive TARE with Y90 resin microspheres. There were no specific inclusion or exclusion criteria. The indication for TARE, the treatment design, the methods used for dose calculation and the follow-up regimen were based on the centres’ internal standards. Participating sites contractually agreed to include all eligible patients consecutively. All included patients signed an informed consent form. This research project was performed in accordance with the ethical standards of the applicable institutional and/or national ethics committees and with the 1964 Helsinki declaration and its later amendments or comparable ethical standards.

Patient recruitment took place between 1 January 2015 and 31 December 2017. Follow-up data were collected until 31 December 2019. Sites were requested to follow-up with the patient every 3 months up to 24 months after the first TARE treatment. In addition, sites were encouraged to obtain follow-up information from referring physicians if follow-up evaluations were not performed at the site of the TARE treatment.

### Assessments

At the time of first treatment, baseline data, demographics and treatment-related data were collected. Information concerning post-TARE treatments, safety data and time-to-event data were gathered at every follow-up visit. Time-to-event was defined from the date of the first TARE treatment until the date of the event. Liver function was described using the albumin-bilirubin (ALBI) formula developed by Johnson *et al.*: ALBI score = (log^10^ bilirubin [μmol/L] × 0.66) + (albumin [g/L] × −0.0852). ALBI score ≤−2.60 is grade 1, >−2.60 to ≤−1.39 is grade 2, and >−1.39 is grade 3.^27^ BCLC classifications were determined at the sites, but all classifications were evaluated according to the uniform BCLC staging standards set out by Reig *et al.* in the recent (2022) update.^28^ Where necessary, patients were re-classified. Information on whether portal vein thrombosis (PVT) was malignant was not collected, but lobar and main PVT were considered malignant, while segmental PVT was considered malignant if the site classified the patient as BCLC C. Safety outcomes are described as severe day-of-treatment complications and occurrences of any adverse events after treatment, according to the Common Terminology Criteria for Adverse Events, version 4.03. Pre-defined serious adverse events (grade 3 and 4) were abdominal pain, fatigue, fever, nausea, vomiting, gastrointestinal ulceration, gastritis, radiation cholecystitis, radiation pancreatitis and radioembolization-induced liver disease (REILD). An open text field allowed us to collect details on other serious adverse events.

### Statistical analysis

Data are presented as mean ± SD or median (IQR) for continuous variables and number (%) for categorical variables. Percentages are based on the whole cohort (N = 422) unless otherwise indicated. Patients who died during the study were categorized as having progression for the purpose of PFS and hPFS analysis. Patients alive and progression-free were censored on the day of last follow-up. The simultaneous occurrence of hepatic progression and extrahepatic progression was considered as hepatic progression.

Comparisons between groups were performed using the log-rank test (Mantel-Haenszel version). The median OS, PFS and hPFS times were calculated with their associated 95% CIs. The group effect was calculated with a Cox proportional-hazards model with hazard ratio (HR) and 95% CIs. The development over time of the ALBI, bilirubin, albumin and international normalized ratio (INR) values were explored using a linear mixed model.

A multivariable analysis for OS, PFS and hPFS was performed using a Cox proportional-hazards model whereby the selection of variables was determined following a univariable analysis and a stepwise variable selection procedure, with a significance level of 0.2 used to determine whether to enter a predictor into the stepwise model. The model with the lowest Akaike information criterion value was considered the final model. All available data were used, and no imputations of missing data were made.

Additional analyses were performed to evaluate the impact on OS, PFS and hPFS of the two main methods to calculate the prescribed Y90 activity: partition model and (modified) body surface area ([m]BSA) methodology. For the comparison of partition model dosimetry (n = 177) with BSA/mBSA (n = 245), we considered a locally modified version of the partition model (n = 3) and voxel-based dosimetry (n = 1) as following the partition model. To compare the two groups, a propensity score analysis was performed. The propensity score is the probability of treatment assignment conditional on measured baseline covariates. Two approaches were used for the propensity score:

1. Matching: Greedy nearest neighbour matching within a calliper of 0.2 of the propensity score was used. Using this approach, a patient treated with partition model dosimetry is selected. This treated patient is then matched with a patient treated based on the BSA activity calculation, whose propensity score is closest to that of the treated patient, subject to the constraint that the differences between their propensity scores are less than a specified maximum (the calliper distance). To estimate the marginal treatment effect for OS, PFS and hPFS, a Cox model with a robust variance estimator that accounts for clustering within matched pairs was used.
2. Inverse probability of treatment weighting (IPTW): IPTW using the propensity score uses weights based on the propensity score to create a synthetic sample in which the distribution of measured baseline covariates is independent of treatment assignment.

To obtain appropriate estimates of variance, stabilised weights were used. For each patient, the stabilised weight is calculated by multiplying his or her original weight by the proportion of patients who received the treatment that he or she received. A Cox model, adjusted for stabilised weights, was used to estimate the relative treatment effect for OS, PFS, and hPFS.

A standardised difference between the two groups for each patient characteristic of interest was calculated to assess whether the covariates are well balanced between the partition model and BSA/mBSA models. The balance between groups was achieved if the magnitude of the standardised difference was less than 0.25.

## Results

### Patient demographics

Four hundred and twenty-two patients with HCC from 25 centres in eight countries were included in this study (Table 1). The median follow-up time was 11.1 months, and 116/422 (27.5%) patients were censored before 24 months due to lack of follow-up information. The mean age of our cohort was 67 years and 341/422 (80.8%) patients were male. In general, the patient population was representative for TARE, with 115/422 (27.3%) patients in BCLC stage B and 247/422 (58.5%) in BCLC stage C; Eastern Cooperative Oncology Group (ECOG) performance status 0 (260/422, 61.6%) or 1 (131/422, 31.0%); and a preserved liver function with ALBI grade 1 (139/422, 32.9%) or 2 (219/422, 51.9%). Cirrhosis was found in 299/422 (70.9%) patients and ascites in 61/422 (14.5%). Thirty-two percent (136/422, 32.2%) had a single tumour nodule, 138/422 (32.7%) had two to five nodules and 72/422 (17.1%) had more than five tumour nodules. Data on exact tumour size was not collected.

**Table 1:** Baseline patient characteristics.

Category	Subcategory	HCC (N = 422)
Sex	n (%)	415 (98.3)
	Male	341 (80.8)
	Female	74 (17.5)
Age (years)	n (%)	422 (100)
	Mean ± SD (range)	67.0 ± 10.7 (22-92)
	Median (IQR)	68 (60–74)
ECOG performance status	n (%)	422 (100)
	0	260 (61.6)
	1	131 (31.0)
	2	31 (7.4)
Cirrhosis	n (%)	422 (100)
	Yes	299 (70.9)
	No	123 (29.1)
Ascites	n (%)	422 (100)
	Yes	61 (14.5)
	No	361 (85.5)
Number of nodules	n (%)	422 (100)
	1	136 (32.2)
	2–5	138 (32.7)
	>5	72 (17.1)
	Uncountable	76 (18.0)
Location of tumour	n (%)	422 (100)
	Bilobar	150 (35.5)
	Left only	51 (12.1)
	Right only	221 (52.4)
Extrahepatic metastases	n (%)	422 (100)
	Yes	36 (8.5)
	No	386 (91.5)
Portal vein thrombosis	n (%)	422 (100)
	Patent	284 (67.3)
	Segmental	81 (19.2)
	Lobar	38 (9.0)
	Main	19 (4.5)
BCLC stage	n (%)	422 (100)
	A	54 (12.8)
	B	115 (27.3)
	C	247 (58.5)
	D	6 (1.4)
ALBI grade	n (%)	373 (88.4)
	1	139 (32.9)
	2	219 (51.9)
	3	15 (3.6)
Prior locoregional procedures	n^a^ (%)	233 (55.2)
	Surgery	72 (17.1)
	Percutaneous ablation	60 (14.2)
	TACE	95 (22.5)
	Abdominal radiotherapy	7 (1.7)
	Vascular procedure	15 (3.6)
Prior systemic therapies	n (%)	41 (9.7)
	Sorafenib	37 (8.7)
	Other	4 (0.9)
Intention of TARE	n (%)	422 (100)
	Ablation	17 (4.0)
	Bridge to surgery or transplant	26 (6.2)
	Downsizing	137 (32.5)
	Palliative	242 (57.3)
Bilirubin (mg/dl)	n (%)	419 (99.3)
	Mean ± SD	0.90 ± 0.49
	Median (IQR)	0.82 (0.53–1.17)
	>1.5 mg/dl	42 (10)
Albumin (g/dl)	n (%)	373 (88.4)
	Mean ± SD	3.72 ± 0.54
	Median (IQR)	3.7 (3.4–4.1)
	<3.5 g/dl	104 (24.6)
INR	n (%)	340 (80.6)
	Mean ± SD	1.14 ± 0.21
	Median (IQR)	1.1 (1.04-1.19)
	>1.2	66 (15.6)
ALT (U/L)	n (%)	393 (93.1)
	Mean ± SD	43.48 (30.20)
	Median (IQR)	34.5 (23–55.5)
Creatinine (mg/dl)	n (%)	419 (99.3)
	Mean ± SD	0.99 (0.70)
	Median (IQR)	0.87 (0.74–1.06)

ALBI, albumin-bilirubin; ALT, alanine aminotransferase; BCLC, Barcelona Clinic Liver Cancer; ECOG, Eastern Cooperative Oncology Group; INR, international normalized ratio; TACE, transcatheter arterial chemoembolization; TARE, transarterial radioembolization.

a Patients can have multiple prior locoregional procedures.

Bilobar disease was observed in 35.5% (150/422) of patients, while unilobar disease was primarily right-sided (52.4%, 221/422). Extrahepatic disease was diagnosed in 36/422 (8.5%) patients and PVT was present in 138/422 (32.7%). Prior to TARE, 95/422 (22.5%) patients had been treated with TACE, 72/422 (17.1%) with surgery and 60/422 (14.2%) with percutaneous ablation. Prior systemic therapy was performed in 41/422 (9.7%) patients (sorafenib 37/41, 90.2%).

### Treatment and follow-up

BSA or mBSA were used to determine the prescribed Y90 activity (165/422 [39.1%] and 80/422 [19.0%], respectively) in most patients (Table 2). Partition model dosimetry was used in 177/422 (41.9%) cases. Whole-liver treatment was performed in 123/422 (29.1%) patients, compared to therapies directed to the right lobe, left lobe, or liver segment (177/422 [41.9%], 56/422 [13.3%], 66/422 [15.6%], respectively). The median prescribed activity was 1.40 GBq for whole-liver treatments (IQR 0.99–1.76), 1.20 GBq for right lobe treatments (IQR 1.00–1.49) and 0.73 (IQR 0.54–1.08) for left lobe treatments. Seventy (70/422 [16.5%]) patients had two or more treatment sessions. Treatments after TARE are listed in Table S1. An increase in ALBI, bilirubin and INR, and a decrease in albumin values was observed 3 months after treatment (*p* <0.0001, Fig. S1).

**Table 2:** Treatment-associated parameters.

Category	Subcategory	HCC (N = 422)
Activity administered (GBq)	n (%)	422 (100)
	Whole liver (median; IQR; range)	1.40; 0.99-1.76; 0.18-5
	Right lobe (median; IQR; range)	1.20; 1.00-1.49; 0.37-5.50
	Left lobe (median; IQR; range)	0.73; 0.54-1.08; 0.20-3.00
Target treatment	n (%)	422 (100)
	Whole liver	123 (29.1)
	Right lobe	177 (41.9)
	Left lobe	56 (13.3)
	Segmental	66 (15.6)
Target tumour volume (ml)	n (%)	393 (93.1)
	Median; IQR; range	142; 53-341; 3-3642
Target liver volume (ml)	n (%)	396 (93.8)
	Median; IQR; range	1,663; 1,356-2,100; 281-5,460
Number of treatments	n (%)	422 (100)
	1	354 (83.9)
	2	66 (15.6)
	3 or more	4 (0.9)
Dose methodology	n (%)	422 (100)
	BSA	165 (39.1)
	Modified BSA	80 (19.0)
	Partition model	177 (41.9)
Embolization before treatment	n (%)	355 (84.1)
	Yes	138 (32.7)
	No	217 (51.4)

BSA, body surface area; HCC, hepatocellular carcinoma.

### Effectiveness

**Fig. 1:**
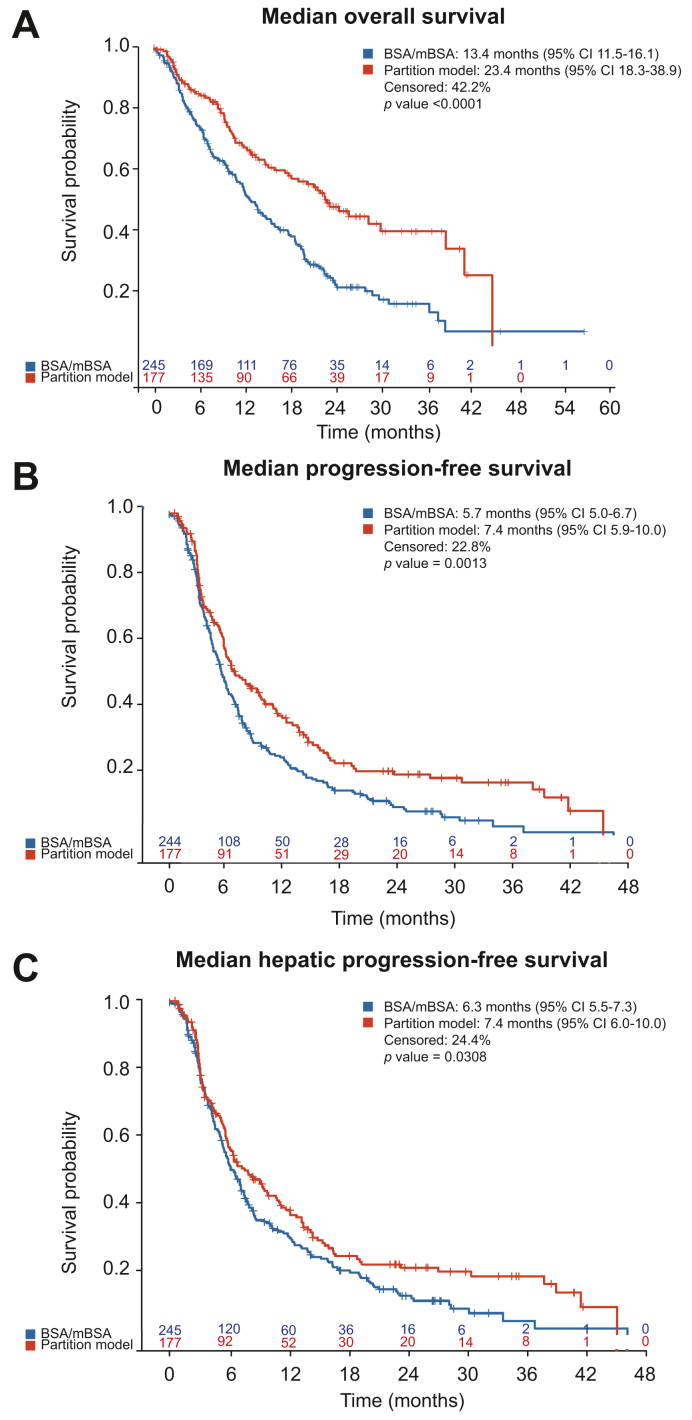
Kaplan–Meier curves comparing outcomes between partition model dosimetry and (modified) body surface area dose calculation. Comparison of (A) overall survival, (B) progression-free survival and (C) hepatic progression-free survival (log-rank test [Mantel-Haenszel version], unadjusted).

The median OS was 16.5 months (95% CI 14.2-19.3), median PFS was 6.1 months (95% CI 5.7-7.0), and median hPFS was 6.7 months for the entire population (95% CI 5.9-7.6). Survival was highest in patients with BCLC A (41.4 months; 95% CI 22.5-ND; *p* <0.0001) (Table 3). The subgroup of patients with ALBI grade 1 lived longer (21.1 months; 95% CI 19.2-28.8; *p <*0.0001) than those with higher ALBI grades (grade 2: 14.0 months; 95% CI 11.5-16.5; *p =* 0.0005; grade 3: 7.8 months; 95% CI 2.7-12.9; *p <*0.0001). The number of tumour nodules, tumour location, presence of extrahepatic metastases, and PVT were also associated with survival (Table 3), as well as dose methodology (Fig. 1). If the prescribed activity was determined by the BSA/mBSA method, median OS was 13.4 months (95% CI 11.5-16.1) as compared to the partition model with a median OS of 23.4 months (95% CI 18.3-38.9; *p <*0.0001, see below for the subgroup analysis). The analysis of PFS and hPFS according to covariates is shown in Tables S2 and S3.

**Table 3:** Univariable analysis for overall survival.

Variable	Threshold	Median (95% CI)	*p* value	HR (95% CI)	*p* value HR
Age (year)	<68	17.2 (14.0-20.3)	0.4356		
	≥68	15.2 (12.2-19.3)		1.11 (0.86-1.42)	0.4340
Sex	Female	18.7 (14.4-23.0)	0.3421	0.85 (0.60-1.20)	0.3444
	Male	15.4 (12.9-19.3)			
ECOG	0	20.7 (18.6-24.4)	**<0.0001**		
	1	12.6 (10.1-14.9)		1.95 (1.49-2.56)	**<0.0001**
	2 + 3	8.1 (6.2-11.4)		2.98 (1.87-4.76)	**<0.0001**
Cirrhosis	No	19.2 (15.0-22.9)	0.1078		
	Yes	14.9 (12.5-19.2)		1.27 (0.95-1.69)	0.1093
Cause of cirrhosis	Alcohol	10.4 (7.9-14.0)	**0.0012**		
	Hepatitis B	20.4 (12.2-ND)		0.51 (0.32-0.83)	**0.0060**
	Hepatitis C	19.7 (13.3-30.2)		0.51 (0.34-0.75)	**0.0007**
	NASH	11.6 (6.7-14.9)		0.97 (0.61-1.53)	0.8829
	Other	20.8 (11.2-23.0)		0.59 (0.36-0.96)	**0.0335**
Ascites	No	18.3 (15.3-20.4)	**0.0009**		
	Yes	9.9 (5.6-14.0)		1.75 (1.25-2.44)	**0.0010**
Number of nodules	1	20.8 (15.4-28.8)	**0.0009**		
	2-5	19.2 (14.0-22.9)		1.33 (0.96-1.84)	0.0851
	>5	13.1 (10.3-18.2)		1.66 (1.14-2.42)	**0.0081**
	Uncountable	10.7 (7.2-12.9)		2.05 (1.42-2.97)	**0.0001**
Location of tumour	Bilobar	11.6 (8.4-16.0)	**0.0001**		
	Left	14.4 (10.3-30.2)		0.65 (0.43-0.99)	**0.0422**
	Right	20.8 (16.5-23.4)		0.55 (0.42-0.73)	**<0.0001**
Extrahepatic disease prior to treatment	No	17.2 (14.4-20.3)	**0.0033**		
	Yes	10.4 (6.8-12.7)		1.81 (1.21-2.71)	**0.0037**
Portal vein thrombosis	Lobar	10.0 (6.1-16.1)	**0.0038**	1.77 (1.16-2.71)	**0.0083**
	Main	7.8 (3.9-14.3)		2.14 (1.24-3.71)	**0.0064**
	Patent	19.3 (15.3-20.8)			
	Segmental	15.2 (10.7-20.3)		1.13 (0.82-1.57)	0.4489
BCLC stage	A	41.4 (22.5-ND)	**<0.0001**		
	B	20.4 (14.9-24.9)		1.96 (1.17-3.28)	**0.011**
	C	12.6 (10.4-14.4)		3.35 (2.08-5.41)	**<0.0001**
	D	12.5 (4.0-.)		2.69 (0.79-9.10)	**0.1126**
Total bilirubin (mg/dl)	≤1.5	17.2 (14.4-20.0)	**0.0086**		
	>1.5	10.0 (4.7-14.4)		1.69 (1.14-2.51)	**0.0094**
Prior locoregional procedures	No	15.3 (12.6-19.2)	0.2126		
	Yes	17.9 (13.4-20.4)		0.85 (0.66-1.10)	0.2134
Prior surgery	No	15.3 (12.9-18.3)	**0.0248**		
	Yes	23.0 (13.4-36.8)		0.67 (0.47-0.95)	**0.0258**
Prior ablation	No	15.2 (12.9-18.7)	**0.0379**		
	Yes	22.4 (16.5-ND)		0.65 (0.43-0.98)	**0.0394**
Prior TACE	No	16.5 (14.0-19.6)	0.9520		
	Yes	16.0 (11.2-20.8)		1.01 (0.75-1.36)	0.9509
Prior abdominal radiotherapy	No	16.1 (14.0-19.2)	0.4204		
	Yes	29.3 (3.2-ND)		0.67 (0.25-1.80)	0.4229
Other prior embolotherapies	No	16.5 (14.0-19.3)	0.7285		
	Yes	14.4 (3.9-ND)		1.13 (0.58-2.19)	0.7288
Prior chemotherapy	No	17.0 (14.3-20.0)	0.0592		
	Yes	10.4 (7.1-19.2)		1.48 (0.98-2.22)	0.0609
Treatment intention	Curative∗	22.9 (18.6-30.2)	**<0.0001**	0.53 (0.40-0.69)	**<0.0001**
	Palliative	12.2 (10.4-14.9)			
Dose methodology	BSA/mBSA	13.4 (11.5-16.1)	**<0.0001**		
	Partition model	23.4 (18.3-38.9)		0.53 (0.41-0.70	**<0.0001**
ALBI grade	1	21.1 (19.2-28.8)	**<0.0001**		
	2	14.0 (11.5-16.5)		1.66 (1.25-2.22)	**0.0005**
	3	7.8 (2.7-12.9)		3.92 (2.11-7.26)	**<0.0001**

Levels of significance: *p* <0.05 (Log-rank test [Mantel-Haenszel version]). Values in bold denote statistical significance.

ALBI, albumin-bilirubin; BCLC, Barcelona Clinic Liver Cancer; BSA, body surface area; ECOG, Eastern Cooperative Oncology Group; HR, hazard ratio; NASH, non-alcoholic steatohepatitis; TACE, transcatheter arterial chemoembolization; TARE, transarterial radioembolization.

∗ Curative reflects treatments for which there is a potential pathway to cure, *e.g*. bridging or downsizing for surgery or transplantation.

The multivariable analysis showed that, after controlling for the other variables, statistically significant variables predicting overall survival were ECOG >0 (ECOG 1: HR 1.64; 95% CI 1.20-2.23; *p =* 0.0018), presence of ascites (HR 1.62; 95% CI 1.10-2.38; *p =* 0.0152), cirrhosis (HR 1.39; 95% CI 1.00-1.194), extrahepatic disease (HR 1.55; 95% CI 1.01-2.27) and PVT (segmental PVT: HR 1.46; 95% CI 1.02-2.10; *p =* 0.0378; and main PVT: HR 2.49; 95% CI 1.37-4.54; *p =* 0.0028), right-sided tumours (HR 0.56; 95% CI 0.41-0.76; *p =* 0.0002), ALBI grade >1 (grade 2: HR 1.56; 95% CI 1.15-2.12; *p =* 0.0043; and grade 3: HR 2.80; 95% CI 1.49-5.28; *p =* 0.0014), curative treatment intention (HR 0.66; 95% CI 0.49-0.89; *p =* 0.0071) and partition model (HR 0.65; 95% CI 0.46-0.92; *p =* 0.0144, Table 4). Variables predicting PFS outcomes were BCLC B and C (BCLC B: HR 1.58; 95% CI 1.06-2.34; *p =* 0.025; BCLC C: HR 1.99; 95% CI 1.38-2.86; *p =* 0.0002), presence of cirrhosis (HR 1.31; 95% CI 1.03-1.67; *p =* 0.0312), curative treatment intention (HR 0.55; 95% CI 0.43-0.70; *p <*0.0001) and right-sided liver tumour (HR 0.73; 95% CI 0.57-0.92; *p =* 0.0092) (Table S4). For hPFS, these variables were ECOG 1 (HR 1.32; 95% CI 1.03-1.70; *p =* 0.0274), presence of cirrhosis (HR 1.43; 95% CI 1.11-1.85; *p =* 0.0060), right-sided tumours (HR 0.65; 95% CI 0.51-0.83; *p =* 0.0005), curative treatment intention (HR 0.59; 95% CI 0.46-0.75; *p <*0.0001) and lobar PVT (HR 1.71; 95% CI 1.15-2.56; *p =* 0.0086) (Table S5).

**Table 4:** Multivariable analysis for overall survival.

Variable	Threshold	HR (95% CI)	*p* value
ECOG (*vs.* 0)	1	1.64 (1.20-2.23)	**0.0018**
	2+3	1.86 (1.09-3.16)	**0.0224**
Cirrhosis (*vs.* no)	Yes	1.39 (1.00-1.94)	**0.0480**
Ascites (*vs.* no)	Yes	1.62 (1.10-2.38)	**0.0152**
Location of tumour (*vs.* bilobar)	Left	0.64 (0.40-1.03)	0.0654
	Right	0.56 (0.41-0.76)	**0.0002**
Extrahepatic disease prior to treatment (*vs.* no)	Yes	1.55 (1.01-2.37)	**0.0455**
Portal vein thrombosis (*vs.* patent)	Lobar	1.40 (0.84-2.35)	0.2018
	Main	2.49 (1.37-4.54)	**0.0028**
	Segmental	1.46 (1.02-2.10)	**0.0378**
Treatment intention (*vs.* palliative)	Curative	0.66 (0.49-0.89)	**0.0071**
Dose methodology (*vs.* BSA/mBSA)	Partition model	0.65 (0.46-0.92)	**0.0144**
ALBI grade (*vs.* 1)	2	1.56 (1.15-2.12)	**0.0043**
	3	2.80 (1.49-5.28)	**0.0014**

Levels of significance: *p* <0.05 (Cox proportional-hazards model). Values in bold denote statistical significance. The proportional hazard function of the Cox model was verified. The following variables were considered in the multivariable model: Barcelona Clinic Liver Cancer stage; ECOG status; cirrhosis; ascites; tumour burden (nodules); location of tumour; extrahepatic disease prior to transarterial radioembolization; portal vein thrombosis; total bilirubin (mg/dl); prior surgery; prior ablation; prior chemotherapy; treatment intention; dose methodology; ALBI grade.

ALBI, albumin-bilirubin; BSA, body surface area; ECOG, Eastern Cooperative Oncology Group; HR, hazard ratio.

### Comparing outcomes between partition model dosimetry and BSA/mBSA

**Fig. 2:**
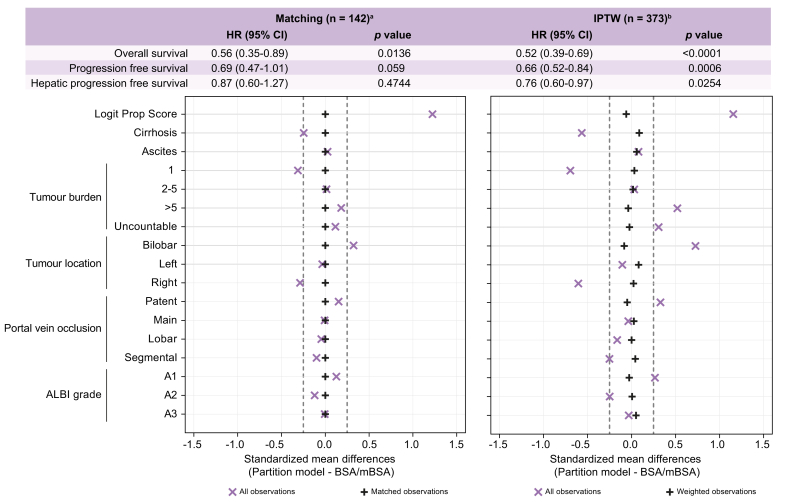
Survival analysis of the propensity score matching. Levels of significance: *p* <0.05 (Cox proportional-hazards model). ^a^Matching: Greedy nearest neighbour matching within a calliper of 0.2 of the propensity score. A total of 142 patients were matched (71 pairs were used), and 231 patients were excluded. The HR and 95% CI for the marginal treatment effect of OS, PFS and hPFS (Cox model with robust variance estimator that accounts for clustering within matched pairs) was presented as follow. The proportional hazard function of the Cox models was verified. The hazard ratio refers to the partition model. ^b^Inverse probability treatment weighting: The HR and 95% CI for the relative treatment effect of OS, PFS and hPFS (Cox model adjusted for stabilised weights) was presented as follow. The proportional hazard function of the Cox models was verified. The hazard ratio refers to the partition model. hPFS, hepatic progression-free survival; HR, hazard ratio; IPTW, inverse probability of treatment weighting; OS, overall survival; PFS, progression-free survival.

To further evaluate the differences in survival outcomes between partition model dosimetry and BSA/mBSA in the univariable analysis and the multivariable analysis for OS, we used propensity score matching to evaluate differences between the patient groups for whom the determination of the prescribed Y90 activity was performed either by BSA/mBSA or the partition model. The covariates considered in the model were based on the outcomes of the multivariable analysis: cirrhosis, ascites, number of tumour nodules, bilobar, right or left-sided tumours, PVT, and ALBI grade (Table S6, see Table S7 for a comparison of all baseline values in the two groups). In the exact matching model, 142 patients were matched (71 pairs were used), and 231 patients were excluded. The standardised mean differences are equal to zero, indicating a high degree of balance of patient characteristics across treatment groups for the application of matching. Compared to BSA/mBSA, the patients treated with the partition model experienced better outcomes in terms of OS (HR 0.56; 95% CI 0.35-0.89; *p =* 0.0136), but not PFS and hPFS (HR 0.69; 95% CI 0.47-1.01; *p =* 0.059 and HR 0.87; 95% CI 0.60-1.27, *p =* 0.4744, respectively) (Fig. 2). For the IPTW, 373 patients were included in the analysis. The smallest standardised difference magnitudes were observed for bilobar tumour location (d = −0.083) and cirrhosis (d = 0.088). All variables had standardised difference magnitudes of less than 0.09, indicating a good degree of balance of patient characteristics across treatment groups for the application of IPTW. The partition model was associated with better outcomes for OS (HR 0.52; 95% CI 0.39-0.69; *p <*0.0001), for PFS (HR 0.66; 95% CI 0.52-0.84; *p =* 0.0006) and for hPFS (HR 0.76; 95% CI 0.60-0.97; *p =* 0.0254), indicating that patients treated based on activity calculations with the partition model had better survival outcomes than patients treated based on BSA/mBSA activity calculations.

Furthermore, we did not find any difference in survival outcomes between patients in the BSA/mBSA cohort that were treated in hospitals that also contributed to the partition model cohort, and patients treated in hospitals that only used BSA/mBSA to calculate the prescribed activity (OS: HR 0.85; 95% CI 0.61-1.18; *p =* 0.3326; PFS: HR 0.94; 95% CI 0.69-1.27; *p =* 0.6684; hPFS: HR 0.98; 95% CI 0.72-1.33; *p =* 0.9) (Table S8).

Finally, comparing the prescribed activity between the partition model and BSA/mBSA revealed no significant differences when adjusted for tumour burden (Fig. S2) and the number of tumour nodules (Fig. S3).

### Safety

A total of 115/422 (36.7%) patients experienced one or more adverse events. Gastrointestinal ulcerations (3/422, 0.7%), gastritis (3/422, 0.7%) and REILD (6/422, 1.4%) were uncommon. Severe adverse events (grade 3-5) were abdominal pain 2.1% (9/422), fatigue 1.4% (6/422), fever 0.5% (2/422), nausea 0.7% (3/422), vomiting 0.5% (2/422), gastrointestinal ulceration 0.2% (1/422), and REILD 0.2% (1/422) (Table S9).

## Discussion

The HCC cohort collected in the CIRT study is one of the largest prospectively collected cohorts on the use of TARE in Europe. Despite the heterogeneous patient population, the multivariable analysis found that, compared with BSA, the partition model was a predictor of improved OS, but not PFS and hPFS. Additional propensity score matching, using the exact matching model and the IPTW model, found that patients whose prescribed activity was calculated with the partition model had better OS and PFS when compared with patients with similar baseline characteristics, but whose activity was prescribed based on BSA. Additionally, the IPTW model found an improved hPFS following the partition model. Other factors influencing OS outcomes were ECOG >0, right-sided tumours, presence of ascites and PVT, and ALBI >1.

The median overall survival of our entire cohort of 422 patients was 16.5 months. This is in the range of 12.8-20.5 months reported by prior studies in similar real-life settings.^15^^,^^17^^,^^18^^,^^29^ On the other hand, in the TARE arms of two prospective studies with randomisation against Sorafenib (SIRveNIB and SARAH), the median OS was only 8.0 and 8.8 months, respectively.^19^^,^^20^ An important reason for the differences in survival is the selection of patients, *e.g*. while only one-third of our patients were classified as BCLC C, the randomised studies recruited a much higher percentage of patients in this advanced group (68% in the SARAH study and 48.4% in the SIRveNIB study in the intention-to-treat population). Finally, our study used Y90 resin microspheres instead of glass microspheres, but a comparison of both spheres in consecutive patients in a single-centre study (resin, n = 41; glass, n = 36) revealed no difference in survival between groups.^22^

In line with previous study outcomes, our multivariable analyses showed that ECOG >0, presence of ascites and extrahepatic disease were negative prognostic factors for OS.[30], [31], [32] Our data also confirms previous findings that the extent of PVT influences OS,^31^^,^[33], [34], [35] although in our analysis, lobar PVT was found to only influence hPFS, while segmental PVT and main PVT were found to impact OS. Our study further establishes that the ALBI grade was a strong independent predictor of OS, mirroring previous findings^27^^,^^36^ and strengthening the justification for its inclusion in the recent BCLC strategy.^28^

Independent prognostic factors associated with OS identified in previous studies were albumin, alpha-fetoprotein, alkaline phosphatase and tumour size <5 cm.^22^^,^^29^^,^^31^^,^^32^^,^^37^ The BCLC stage has also been identified as a prognostic factor for survival outcomes in several prior studies.[37], [38], [39] In our cohort, BCLC staging was a significant predictor of OS, PFS, and hPFS in the univariable analysis, but it was only a predictor for PFS in the multivariable analysis. A possible explanation is that BCLC is a composite variable consisting of variables that were found to be independent predictors, such as ECOG, PVT, and extrahepatic disease, and may thus not be independent from these variables. Additionally, a recent comparison of prognostic scoring systems in a cohort of patients receiving TARE ranked BCLC lower than other prognostic factors for this treatment.^28^ The apparent difference in survival based on tumour location in the right *vs*. the left liver lobe found in our cohort may reflect the complexity and variation of blood supply to the liver, as recently described by Choi *et al.*^40^ The variations in the blood supply of the left liver lobe may require a more meticulous positioning of suited microcatheters to ensure a consistent and robust dose distribution compared to the right liver lobe, explaining differences in outcome if not considered thoroughly.[41], [42], [43]

In terms of safety and toxicity, our cohort confirms previous reports on the favourable safety outcomes of TARE.^15^^,^^29^^,^^33^^,^^37^^,^^44^ We observed a worsening of the liver function after TARE in terms of INR, bilirubin and albumin values (and therefore ALBI score), which mirrors the results of the SORAMIC randomised controlled trial, where in the TARE + sorafenib group, poorer ALBI scores after 4 and 6 months were observed compared to the sorafenib alone group.^45^ Our study reported that 1.4% of patients experienced REILD, which was grade 3 or higher in half of affected patients. This occurrence of REILD is on the lower end of the studies used in the systematic review by Braat *et al.*, who identified that the incidence of symptomatic REILD varied between 0 and 31%, although, in most reports, the incidence was 0–8%.^46^

In our cohort, the multivariable analysis and the propensity score analyses showed that the partition model activity calculation led to better OS outcomes compared to BSA and mBSA, and improved PFS and hPFS in the propensity score analysis. The BSA and mBSA methods rely on an assumed correlation between BSA and the tumour burden to estimate Y90 activity. Ignoring the variability of the tumour-to-normal-liver ratio in individual patients, it sacrifices accuracy for simplicity,^47^ and may result in wide variations of radiation dose absorbed by both the tumour and the surrounding non-tumoural liver parenchyma.^48^^,^^49^ On the other hand, personalised dosimetry such as partition model relies on differential tumour-to-non-tumour perfusion evaluated on pre-treatment Technetium-99m-macroaggregated albumin single-photon emission computer tomography combined with computer tomography to predict dose distributions between the “partitions” tumoural liver, non-tumoural liver, and lung. It has been demonstrated that personalised dosimetry models can increase the tumour-absorbed dose while keeping the dose in the non-tumoural liver and the lung low.^22^ However, compared to the BSA model, these models are very resource-intensive and require a good collaboration between nuclear medicine physicians and interventional radiologists. Nevertheless, the data presented in this study strongly suggests that partition model dosimetry improves OS, PFS and hPFS outcomes in patients with unresectable HCC. These outcomes reflect recent studies examining the relationship between tumour-absorbed dose and survival outcomes in patients with HCC.

The randomised phase II DOSISPHERE-01 trial comparing patients with unresectable locally advanced HCC receiving personalised dosimetry with standard dosimetry showed that objective response was achieved in 20/28 patients (71%; 95% CI 51-87) in the personalised dosimetry group *vs.* 10/28 (36%; 95% CI 19-56) in the standard dosimetry group (*p =* 0.0074). This translated into a median OS of 26.6 months (95% CI 11.7-NR) in the personalised dosimetry group compared to 10.7 months (95% CI 6.0-16.8) in the standard dosimetry group. Furthermore, patients who received a tumour dose of 205 Gy or higher had an OS of 26.6 months (95% CI 13.5-NR) compared to 7.1 months (95% CI 4.6-14.8) in those that received a tumour dose of less than 205 Gy (HR 0.33; 95% CI 0.15-0.71; *p =* 0.0029).^23^ Of note is that to achieve a high tumour-absorbed dose without increasing the dose absorbed by the non-tumoural liver, the DOSISPHERE-01 trial included only patients with tumours showing arterial phase hyperenhancement. Additionally, a secondary analysis of 120 patients from the SARAH study showed that participants who received at least 100 Gy (n = 67) had longer OS than those who received less than 100 Gy (median, 14.1 months [95% CI 9.6-18.6] *vs.* 6.1 months [95% CI 4.9-6.8], respectively; *p =* 0.001). In the patient group that was available for response analysis (n = 109), tumour radiation-absorbed dose was higher in patients with disease control *vs.* those with progressive disease (median, 121 Gy [IQR 86–190 Gy] *vs*. 85 Gy [IQR 58–164 Gy]; *p =* 0.02).^50^ Unfortunately, the present study did not collect any data on the tumour target dose or radiation-absorbed dose and thus cannot provide any suggestions on optimal dosage. Analysis of differences in prescribed activity between BSA/mBSA and the partition model found no significant differences, when adjusted for tumour volume, percentage of tumour activity and number of tumour nodules. Nevertheless, the multivariable analysis and the propensity score matching suggest that patients whose dosages were calculated with the partition model generally performed better than patients whose dosage was calculated with BSA or mBSA, which is in line with the aforementioned studies. Our study adds to the findings from DOSISPHERE-01 and SARAH, by showing that personalised dosimetry methods also improve effectiveness outcomes in a real-life clinical context with Y90 resin microspheres, compared to activity calculation methods based on BSA, irrespective of a site’s experience.

A limitation of the study is the observational study design, whereby important confounding factors may not have been accounted for. The heterogeneity of the patient population reflects the real-life clinical practice in participating centres and thus its diversity in patient selection and clinical outcomes. We used the propensity score method and multivariable analysis to alleviate, to some degree, the effect of this heterogeneity and multiple methods of analysis were used to show the similarity of outcomes despite the differences in analysis. Of course, certain confounding factors which were not considered in these methods could have contributed to the outcomes and should be considered when interpreting the results.

The study was designed to explore the clinical outcomes of TARE and therefore focused less on dosimetry-specific data. This means that retrospectively important data points such as precise administered activity and tumour-absorbed dose were not included in the evaluation at the time of study design. Furthermore, as an observational study, the design was non-prescriptive for tumour response assessment, which was performed with various criteria (*e.g.*, RECIST, modified RECIST or PET Response Criteria in Solid Tumours) according to local practice and expertise of centres. This prevented us from including tumour response in the analysis.

We attempted to collect quality-of-life data from patients on a voluntary basis at the time of treatment and at every follow-up visit until study exit. The relevance of the collected dataset is currently being evaluated. The relatively high number of patients lost to follow-up can introduce bias regarding the interpretation of OS, and imprecise follow-up imaging intervals should be considered when interpreting PFS and hPFS. A potential explanation might be that TARE requires a comprehensive infrastructure with patients being referred to specialised centres for the treatment while being followed up by their local physician. In those cases, sites were encouraged to obtain follow-up information by contacting the referring physician. If this was not possible, the patient was considered as lost to follow-up. Selection bias can be expected in an observational study and regular remote monitoring was performed to verify that all eligible patients were included. Remote monitoring was done to improve data quality; however, no source data verification was performed.

This large prospective observational data set suggests that TARE with resin Y90 microspheres has a favourable toxicity profile and that patients with good liver function and no extrahepatic disease are ideal candidates for this therapy. Furthermore, our data revealed that optimising the application of the therapy by using the partition model instead of BSA models, can significantly improve survival outcomes. It is thus recommended that activity calculations with the partition model are considered when designing future randomised controlled trials on TARE.

## Financial support

The CIRT study was funded by an independent investigator-initiated research grant from SIRTEX Medical Europe GmbH (Bonn, Germany). CIRSE, the Cardiovascular and Interventional Radiological Society of Europe, is responsible for the independent execution of the CIRT study and has sole ownership of the data.

## Authors’ contributions

FK, DA, BP, BS, NS, GMa, GMu, TH and NdJ contributed to the study concept, set up, and design. DA, RG, MP, TP, MR, BS, GMa and TH acquired patient data. FK, DA, RG, MR, BS, HP, NS, BZ and NdJ analysed and interpreted the data. FK, MR, BS, NS, HP, BZ, NdJ and TH drafted the manuscript. TH supervised the study. FK supervised the manuscript drafting and data interpretation. All authors contributed to critical revisions and approved the final version of the manuscript.

## Data availability statement

Data access is limited by ethical and regulatory considerations.

## Conflict of interest

Frank Kolligs participated on a data safety monitoring or advisory board of Bayer, MSD, and Roche. Dirk Arnold received consulting fees and honoraria for presentations and lectures and travel support from Boston Scientific and Terumo, is on the guidelines committee of the European Society for Medical Oncology, and supported oncopolicy manuscripts for the European Cancer Organisation. Rita Golfieri participated on a Data Safety Monitoring Board or Advisory Board and received payment or honoraria for lectures, presentations, speakers’ bureaus, manuscript writing or educational events from Roche, Guerbet and Sirtex. Maciej Pech received grants or contracts and honoraria from lectures from Sirtex and Bayer. Maxime Ronot received honoraria for lectures from GE Healthcare, Ipsen, Canon-Toshiba, Alexion Pharmaceuticals, Guerbet, and Sirtex. Bruno Sangro received grants or contracts from Sirtex and BMS, consulting fees from Adaptimmune, Astra Zeneca, Bayer, BMS, Boston Scientific, Eisai, Eli Lilly, Incyte, Ipsen, Roche, Sirtex Medical, Terumo; Payment or honoraria for lectures, presentations, speakers bureaus, manuscript writing or educational events from Astra Zeneca, Bayer, BMS, Eisai, Incyte, Ipsen, Roche, Sirtex Medical; Participation on a data safety monitoring board or advisory board from Adaptimmune, Astra Zeneca, Bayer, BMS, Boston Scientific, Eisai, Eli Lilly, Incyte, Ipsen, Roche, Sirtex Medical, Terumo, and has a leadership or fiduciary role in the International Liver Cancer Association. Geert Maleux received honoraria for speaker’s bureau from Sirtex Medical and operated as proctor for Sirtex. Bora Peynircioglu, Thomas Pfammatter, Niklaus Schaefer, Graham Munneke, Helena Pereira, Bleranda Zeka, Niels de Jong and Thomas Helmberger had nothing to declare.

Please refer to the accompanying ICMJE disclosure forms for further details.
