# Supplementary material for: Factors impacting survival after transarterial radioembolization in patients with hepatocellular carcinoma: Results from the prospective CIRT study
Source: JHEP Rep. 2022 Nov 25;5(2):100633. doi: 10.1016/j.jhepr.2022.100633 (PMC9804139; doi:10.1016/j.jhepr.2022.100633)
Supplement: Multimedia component 1 [file mmc1.pdf]

Journal of Hepatology, Volume *5*

Supplemental information

Factors impacting survival after transarterial radioembolization in patients with hepatocellular carcinoma: Results from the prospective CIRT study

Frank Kolligs, Dirk Arnold, Rita Golfieri, Maciej Pech, Bora Peynircioglu, Thomas Pfammatter, Maxime Ronot, Bruno Sangro, Niklaus Schaefer, Geert Maleux, Graham Munneke, Helena Pereira, Bleranda Zeka, Niels de Jong, Thomas Helmberger, and on behalf of the CIRT Principal Investigators

Factors impacting survival after transarterial radioembolization in patients with hepatocellular carcinoma: Results from the prospective CIRT study

Frank Kolligs, Dirk Arnold, Rita Golfieri, Maciej Pech, Bora Peynircioglu, Thomas Pfammatter, Maxime Ronot, Bruno Sangro, Niklaus Schaefer, Geert Maleux, Graham Munneke, Helena Pereira, Bleranda Zeka, Niels de Jong, Thomas Helmberger, on behalf of the CIRT principal investigators

Table of contents

CIRT principal investigators..2

Supplementary figures..4

Supplementary tables...7

CIRT principal investigators

Thomas Albrecht, Vivantes Klinikum Neukölln, Department for Radiology and Interventional Therapy, Rudower Str. 48, 12351, Berlin, Germany.

Olivier D'Archambeau, University Hospital Antwerp, Department of Radiology, Wilrijkstraat 10, 2650, Antwerp, Belgium.

Tugsan Balli, Cukurova University, Radiology Department, Balcalı Hospital, 01330, Adana, Turkey.

Sadik Bilgic, Ankara University, Department of Radiology, Medical Faculty, Cebeci, 06590, Ankara, Turkey.

Allan Bloom, Hadassah-Hebrew University Medical Center, Department of Radiology, Jerusalem, Israel.

Roberto Cioni, University of Pisa, Diagnostic and Interventional Radiology, Department of Translational Research and New Technologies in Medicine, Via U. Foscolo 5, 50059, Vinci, Pisa, Italy.

Roman Fischbach, Asklepios Klinik Altona, Department of Radiology and Neuroradiology, Paul-EhrlichStraße 1, 22763, Hamburg, Germany.

Patrick Flamen, Institute Jules Bordet, Université Libre de Bruxelles, Nuclear Medicine Department, 121 Boulevard de Waterloo, 1000, Brussels, Belgium.

Laurent Gerard, University Hospital of Liege, Division of Radiology, domaine du Sart-Tilman B35, 4000, Liège, Belgium.

Gerd Grözinger, Eberhard Karls University, Department of Diagnostic and Interventional Radiology, Hoppe-Seyler-Str. 3, D-72076, Tübingen, Germany.

Marcus Katoh, Helios Hospital Krefeld, Department of Diagnostic and Interventional Radiology, Lutherplatz 40, 47805, Krefeld, Germany.

Michael Koehler, University Hospital Muenster, Department of Clinical Radiology, Albert-Schweitzer-Strasse 33, 48129, Muenster, Germany.

Jan Robert Kröger, Johannes Wesling Klinik Minden, Universitätsinstitut für Radiologie, Neuroradiologie und Nuklearmedizin der Mühlenkreiskliniken, Hans-Nolte-Straße 1, 32429 Minden, Germany.

Christiane Kuhl, University Hospital Aachen, Department of Radiology, Pauwelsstr. 30, 52074, Aachen, Germany.

Franco Orsi, European Institute of Oncology, Interventional Radiology Division, Via Ripamonti 435, 20100, Milan, Italy.

Murat Özgün, St. Franziskus Hospital, Department of Radiology, Hohenzollernring 70, 48145, Muenster, Germany.

Peter Reimer, Academic Teaching Hospital the University of Freiburg, Städtisches Klinikum Karlsruhe, Institute for Diagnostic and Interventional Radiology, Moltkestrasse 90, 76133, Karlsruhe, Germany.

Maxime Ronot, APHP, University Hospitals Paris Nord Val de Seine, Department of Radiology, 100 bd général Leclerc, 100 bd general Leclerc, Beaujon, Clichy, Hauts-de-Seine, France.

Axel Schmid, University Hospital Erlangen, Department of Radiology, Maximiliansplatz 1, 91054, Erlangen, Germany.

Alessandro Vit, Azienda Ospedaliero Universitaria, SOC Diagnostica Angiografica e Radiologia Interventistica, via Grazzano 150/C, 33100, Udine, Italy.

Supplementary figures

Fig. S1. Mean (SD) evolution of ALBI, bilirubin, albumin and INR

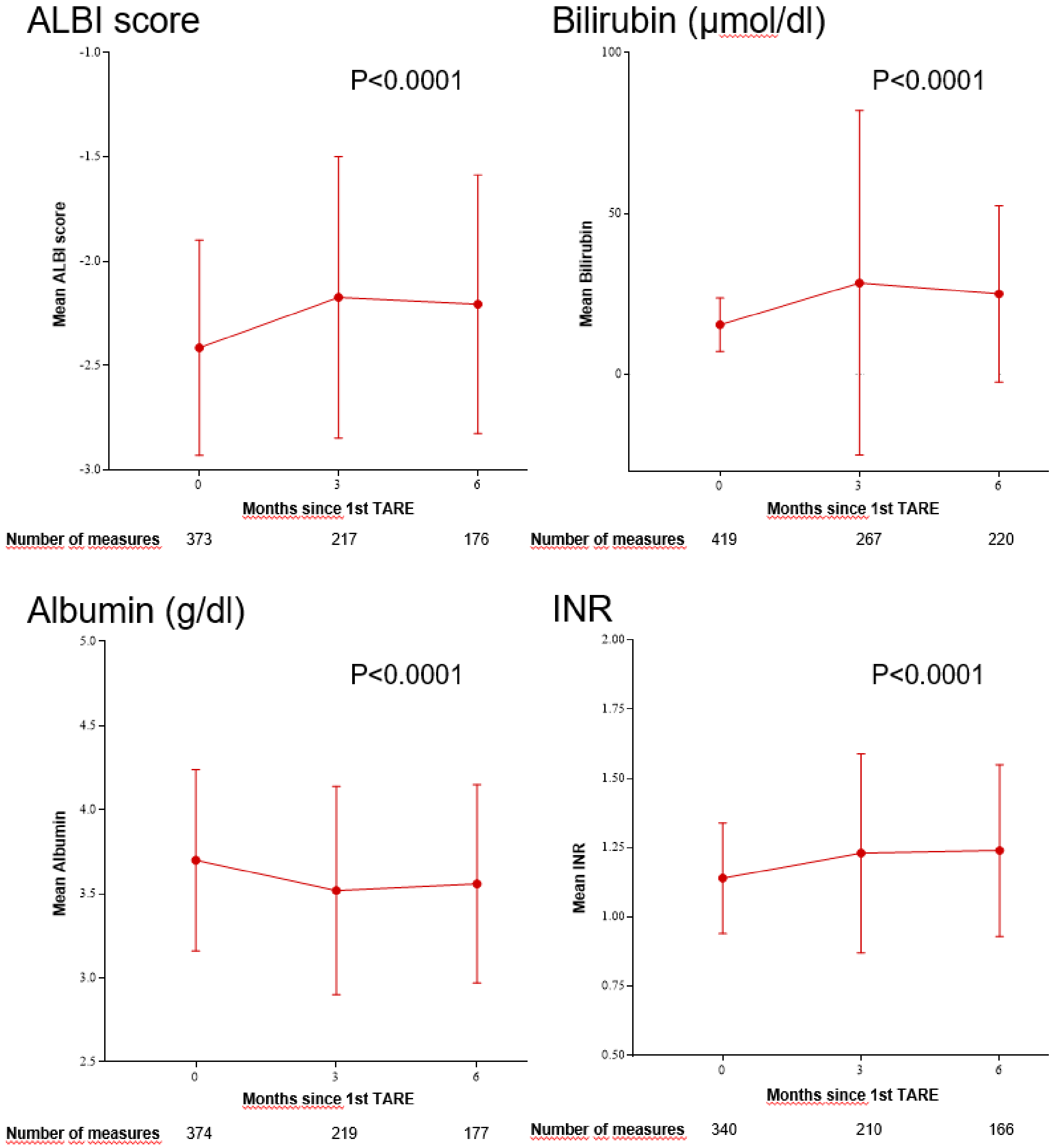

Levels of significance: $p < 0.05$ (Linear mixed model)

A linear mixed model was used to explore time effect for ALBI, bilirubin, albumin and INR with patient's effect as random variable (to take into account between-patient differences).

ALBI: Albumin-Bilirubin; INR: International Normalised Ratio; TARE: Transarterial Radioembolization.

Fig. S2. Comparing the prescribed activity between partition model and BSA/mBSA, adjusted for tumour burden.

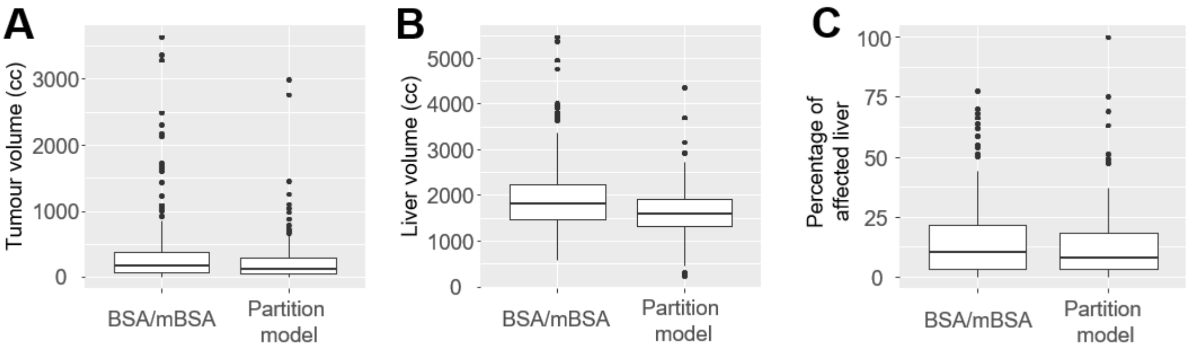

A

Median (IQR)	174 (59-377)	119 (51-294)
n (%)	219 (52)	174 (41)
Missing, n (%)	26 (6)	3 (<1)
P value	0.03	

B

Median (IQR)	1798 (1446-2224)	1587 (1309-1903)
n (%)	219 (52)	177 (42)
Missing, n (%)	26 (6)	0
P value	<0.001	

C

Median (IQR)	9.1 (3.4-20.4)	7.9 (3.3-18.3)
n (%)	216 (51)	174 (41)
Missing, n (%)	29 (7)	3 (<1)
P value	0.16	

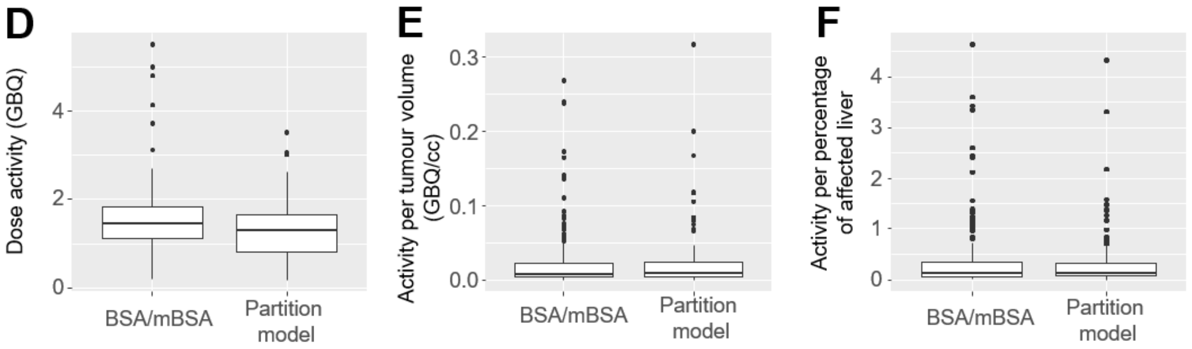

D

Median (IQR)	1.4 (1.0-1.8)	1.3 (0.8-1.7)
n (%)	245 (58)	177 (42)
Missing, n (%)	0	0
P value	0.003	

E

Median (IQR)	0.008 (0.004-0.02)	0.009 (0.005-0.024)
n (%)	216 (51)	174 (41)
Missing, n (%)	29 (7)	3 (<1)
P value	0.24	

F

Median (IQR)	0.13 (0.07-0.34)	0.13 (0.08-0.33)
n (%)	216 (51)	174 (41)
Missing, n (%)	29 (7)	3 (<1)
P value	0.81	

Levels of significance: $p < 0.05$ (Wilcoxon-Mann-Whitney-Test).

We compared the prescribed activity (GBq) between patients whose prescribed activity was calculated with BSA or mBSA and partition model. Patients treated with the partition model generally had smaller tumours (**A**, in cc) and livers (**B**) but the percentage of the liver affected by tumour (**C**) was similar as patients treated with BSA/mBSA. The prescribed activity using BSA/mBSA was significantly higher than using partition model (**D**, p=0.003), but adjusted for tumour volume (**E**) or percentage of affected liver (**F**), no significant difference in prescribed dose was found.

Fig. S3. Comparing the prescribed activity between the partition model and BSA/mBSA, adjusted for number of tumour nodules.

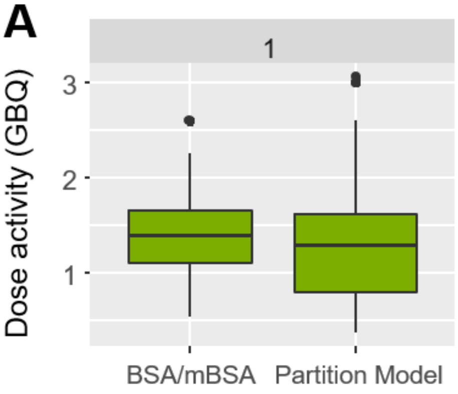

Median (IQR)	1.4 (1.1-1.6)	1.3 (0.8-1.6)
n (%)	47 (11)	89 (21)
Missing, n (%)	0	0
P value	0.22	

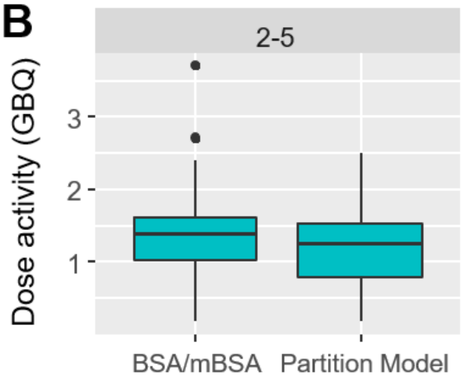

Median (IQR)	1.4 (1.0-1.6)	1.3 (0.8-1.5)
n (%)	81 (19)	57 (14)
Missing, n (%)	0	0
P value	0.12	

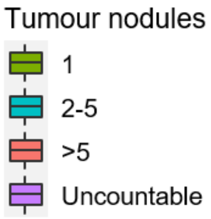

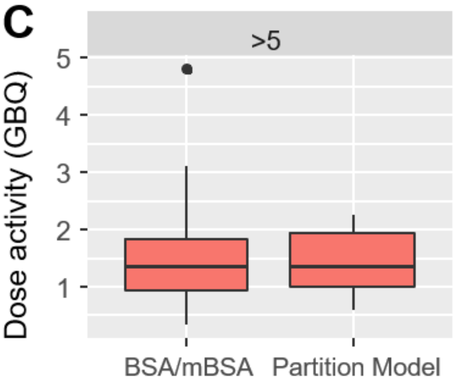

Median (IQR)	1.4 (0.9-1.8)	1.4 (1.0-1.9)
n (%)	61 (15)	11 (3)
Missing, n (%)	0	0
P value	0.75	

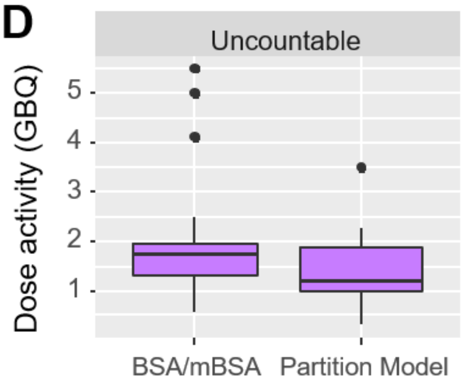

Median (IQR)	1.8 (1.3-2.0)	1.2 (1.0-1.9)
n (%)	56 (13)	20 (5)
Missing, n (%)	0	0
P value	0.06	

Levels of significance: $p < 0.05$ (Wilcoxon-Mann-Whitney-Test).

We compared the prescribed activity (GBq) between patients whose prescribed activity was calculated with BSA or mBSA and partition model. Adjusting for number of tumour nodules, we found no significant differences between the prescribed activity using BSA/mBSA or the partition model for number of nodules 1 (**A**), 2-5 (**B**), >5 (**C**), or uncountable (**D**).

Supplementary tables

Table S1. Post-TARE chemotherapies

Category	Subcategory	HCC (n=422)
Post-TARE systemic chemotherapy	Missing	35 (8.3%)
	No	262 (62.1%)
	Yes	125 (29.6%)
Post-TARE systemic chemotherapy: number of lines	Missing	298 (70.6%)
	One line	38 (9.0%)
	Two lines	19 (4.5%)
	Three lines	21 (5.0%)
	Four lines	9 (2.1%)
	Five lines	10 (2.4%)
	Six lines	6 (1.4%)
	More than six lines	21 (5.0%)
Single systemic therapy	Yes	99 (23.5%)
Therapy	SORAFENIB	53 (53.5%)
	REGORAFENIB	21 (21.2%)
	CAPECITABINE	8 (8.1%)
	5-FLUOROURACIL	
	NIVOLUMAB	7 (7.1%)
	GEMCITABINE	4 (4.0%)
	CABOZANTINIB	3 (3.0%)
	LENVATINIB	3 (3.0%)
Multiple systemic therapies	Yes	3 (0.7%)
Number of systemic regimens [a]	1 regimen	2 (66.7%)
	2 regimens	
	3-5 regimens	1 (33.3%)
	6-10 regimens	3 (0.7%)
Type of therapy	GEMOX	3 (100.0%)
	Not categorized	3 (100.0%)

[a] regimen is understood as a combination of several chemotherapeutic agents
GEMOX: Gemictabin and oxaliplatin; TARE: Transarterial radioembolization

Table S2. Univariable analysis for progression-free survival

Variable	Threshold	Median (95% CI)	p value	HR (95% CI)	p value HR
Age (years)	<68	5.6 (4.7-7.0)	0.4267		
	>=68	6.7 (5.9-7.9)		0.92 (0.74-1.14)	0.4264
Gender	Female	7.0 (4.7-10.3)	0.3161	0.86 (0.65-1.15)	0.3176
	Male	6.0 (5.5-6.8)			
ECOG	0	6.9 (6.0-7.9)	**0.0004**		
	1	5.4 (4.4-6.0)		1.55 (1.23-1.95)	**0.0002**
	2+3	5.7 (3.5-8.5)		1.57 (1.02-2.41)	**0.0388**
Cirrhosis	No	7.0 (5.6-9.5)	0.1285		
	Yes	6.0 (5.5-6.7)		1.21 (0.95-1.54)	0.1293
Cause of cirrhosis	Alcohol	5.7 (4.5-7.2)	**0.0383**		
	Hepatitis-B	4.1 (3.4-6.5)		0.94 (0.64-1.40)	0.7695
	Hepatitis-C	7.7 (5.7-12.2)		0.62 (0.44-0.87)	**0.0051**
	NASH	5.5 (3.5-7.6)		0.97 (0.64-1.48)	0.8903
	Other	7.1 (4.3-9.7)		0.88 (0.58-1.33)	0.5335
Ascites	No	6.3 (5.7-7.3)	0.1915		
	Yes	5.5 (3.5-6.7)		1.23 (0.90-1.67)	0.1907
Number of nodules	1	7.7 (6.3-9.5)	**0.0019**		
	2-5	6.2 (5.6-7.7)		1.25 (0.95-1.63)	0.1070
	>5	5.3 (4.0-7.3)		1.56 (1.13-2.16)	**0.0066**
	Uncountable	4.7 (3.7-5.8)		1.76 (1.28-2.42)	**0.0005**
Location of tumour	Bilobar	5.3 (4.4-6.1)	**0.0031**		
	Left	6.6 (3.6-8.6)		0.78 (0.55-1.11)	0.1704
	Right	7.0 (5.9-8.6)		0.67 (0.53-0.85)	**0.0007**
Extra-hepatic disease prior to treatment	No	6.3 (5.7-7.2)	0.1314		
	Yes	4.7 (3.3-7.2)		1.33 (0.92-1.92)	0.1318
Portal vein thrombosis	Lobar	5.5 (3.5-6.5)	0.3132	1.34 (0.91-1.95)	0.1346
	Main	4.7 (3.0-8.0)		1.30 (0.79-2.13)	0.2989
	Patent	6.7 (5.7-7.9)			
	Segmental	5.8 (5.0-7.2)		1.16 (0.88-1.52)	0.2957
BCLC stage	A	12.5 (7.7-17.2)	**0.0002**		
	B	6.0 (5.3-7.6)		1.74 (1.17-2.57)	**0.0058**
	C	5.6 (4.7-6.1)		2.17 (1.51-3.11)	**<0.0001**
	D	11.2 (4.0-12.7)		1.37 (0.48-3.86)	0.5558
Total bilirubin (mg/dl)	≤1.5	6.2 (5.7-7.2)	0.3282		
	>1.5	4.6 (3.3-8.4)		1.20 (0.83-1.74)	0.3293
Prior procedures	No	6.3 (5.5-7.2)	0.8554		
	Yes	6.0 (4.9-7.6)		0.98 (0.79-1.22)	0.8575
Surgery	No	6.3 (5.7-7.0)	0.7168		
	Yes	5.8 (4.3-7.6)		0.95 (0.72-1.26)	0.7189
Ablation	No	5.8 (5.5-6.6)	**0.0468**		
	Yes	9.2 (6.0-14.0)		0.72 (0.52-1.00)	**0.0478**
TACE	No	6.3 (5.7-7.2)	0.2557		
	Yes	5.8 (3.7-8.6)		1.16 (0.90-1.49)	0.2555
Abdominal radiotherapy	No	6.0 (5.6-7.0)	0.2298		
	Yes	11.6 (3.0-ND)		0.59 (0.24-1.42)	0.2351

Other prior embolotherapies	No	6.3 (5.7-7.2)	0.3602		
	Yes	4.0 (3.0-5.9)		1.30 (0.75-2.26)	0.3575
Prior chemotherapy	No	6.3 (5.7-7.3)	0.1889		
	Yes	5.2 (3.5-6.5)		1.27 (0.89-1.81)	0.1897
Treatment intention	Ablation	13.6 (5.8-30.4)	**<0.0001**	0.43 (0.23-0.79)	**0.0068**
	Bridge to surgery or transplant	7.6 (4.4-ND)		0.44 (0.26-0.74)	**0.0021**
	Down-sizing/down-staging	9.5 (6.7-13.6)		0.55 (0.43-0.70)	**<0.0001**
	Palliative	5.3 (4.5-5.8)			
Treatment intention	Curative	9.5 (7.2-13.6)	**<0.0001**	0.52 (0.41-0.65)	**<0.0001**
	Palliative	5.3 (4.5-5.8)			
Dose methodology	BSA/mBSA	5.7 (5.0-6.7)	**0.0013**		
	Partition model	7.4 (5.9-10.0)		0.70 (0.56-0.87)	**0.0014**
ALBI grade	1	6.8 (5.7-8.1)	**0.0209**		
	2	5.9 (5.3-6.7)		1.09 (0.86-1.38)	0.4665
	3	4.6 (2.6-6.6)		2.18 (1.24-3.80)	**0.0064**

Levels of significance: p <0.05 (Log-rank test [Mantel-Haenszel version]).
ALBI: Albumin Bilirubin; BCLC Barcelona Clinic Liver Cancer; BSA: Body Surface Area; ECOG: Eastern Cooperative Oncology Group; NASH: Non-alcoholic steatohepatitis; TACE: Transcatheter Arterial Chemoembolization; TARE: Transarterial Radioembolization.

Table S3. Univariable analysis for hepatic progression free survival

**Variable**	**Threshold**	**Median (95% CI)**	**p value**	**HR (95% CI)**	**p value HR**
Age (years)	<68	6.0 (5.3-7.6)	0.3446		
	>=68	7.2 (6.2-8.5)		0.90 (0.73-1.12)	0.3445
Gender	Female	7.7 (5.4-12.3)	0.3924	0.88 (0.66-1.18)	0.3935
	Male	6.6 (5.8-7.4)			
ECOG	0	7.3 (6.3-8.7)	**0.0016**		
	1	5.7 (5.2-7.4)		1.45 (1.14-1.84)	**0.0021**
	2+3	5.7 (3.5-8.5)		1.70 (1.11-2.61)	**0.0152**
Cirrhosis	No	7.9 (6.3-12.3)	**0.0292**		
	Yes	6.2 (5.7-7.2)		1.32 (1.03-1.69)	**0.0297**
Cause of cirrhosis	Alcohol	5.9 (4.6-7.7)	0.0854		
	Hepatitis-B	4.1 (3.4-6.6)		0.99 (0.67-1.47)	0.9642
	Hepatitis-C	7.7 (5.7-12.2)		0.64 (0.46-0.90)	**0.0111**
	NASH	5.7 (4.3-8.7)		0.89 (0.58-1.35)	0.5722
	Other	8.2 (5.2-12.0)		0.83 (0.54-1.26)	0.3811
Ascites	No	7.0 (6.0-8.0)	0.0911		
	Yes	5.5 (3.7-7.2)		1.31 (0.96-1.78)	0.0907
Number of nodules	1	8.0 (6.6-9.7)	**0.0028**		
	2-5	6.8 (5.8-9.5)		1.17 (0.89-1.54)	0.2588
	>5	6.1 (4.2-8.7)		1.47 (1.06-2.03)	**0.0218**
	Uncountable	4.7 (3.7-5.8)		1.76 (1.28-2.42)	**0.0005**
Location of tumour	Bilobar	5.6 (4.7-6.7)	**0.0021**		
	Left	6.7 (4.0-9.2)		0.81 (0.57-1.16)	0.2596
	Right	8.0 (6.5-10.3)		0.66 (0.52-0.83)	**0.0005**
Extra-hepatic disease prior to treatment	No	6.9 (6.0-7.9)	0.1827		
	Yes	5.2 (3.3-8.0)		1.29 (0.89-1.88)	0.1827
Portal vein thrombosis	Lobar	5.5 (3.5-6.5)	0.1533	1.45 (1.00-2.13)	0.0530
	Main	4.7 (3.0-8.0)		1.41 (0.86-2.31)	0.1716
	Patent	7.4 (6.3-8.8)			
	Segmental	6.2 (5.4-8.1)		1.11 (0.84-1.46)	0.4731
BCLC stage	A	12.5 (7.7-21.1)	**0.0013**		
	B	6.6 (5.5-7.9)		1.68 (1.13-2.50)	**0.0105**
	C	5.9 (5.3-7.2)		2.04 (1.41-2.94)	**0.0001**
	D	11.2 (4.0-12.7)		1.44 (0.51-4.08)	0.4892
Total bilirubin (mg/dl)	≤1.5	7.0 (6.0-7.9)	0.1432		
	>1.5	4.6 (3.3-8.4)		1.32 (0.91-1.91)	0.1444
Prior procedures	No	6.6 (5.7-7.7)	0.5326		
	Yes	7.2 (5.8-9.2)		0.93 (0.75-1.16)	0.5349
Surgery	No	6.6 (5.8-7.7)	0.3336		
	Yes	7.6 (4.7-12.0)		0.87 (0.65-1.16)	0.3347
Ablation	No	6.3 (5.7-7.3)	**0.0312**		
	Yes	10.0 (6.7-16.1)		0.69 (0.50-0.97)	**0.0323**
TACE	No	7.0 (6.0-7.7)	0.1999		
	Yes	5.9 (3.7-8.7)		1.18 (0.92-1.52)	0.1999
Abdominal radiotherapy	No	6.7 (5.9-7.6)	0.1239		
	Yes	11.6 (3.0-ND)		0.47 (0.18-1.26)	0.1330

Other prior embolotherapies	No	6.8 (6.0-7.7)	0.4387		
	Yes	4.3 (3.3-13.4)		1.25 (0.72-2.17)	0.4360
Prior chemotherapy	No	7.0 (6.0-8.0)	0.1905		
	Yes	5.7 (3.7-6.8)		1.27 (0.89-1.82)	0.1908
Treatment intention	Ablation	13.6 (7.7-33.6)	**<0.0001**	0.42 (0.22-0.79)	**0.0075**
	Bridge to surgery or transplant	7.6 (4.4-.)		0.49 (0.29-0.84)	**0.0088**
	Down-sizing/down-staging	9.7 (7.2-14.0)		0.59 (0.46-0.76)	**<0.0001**
	Palliative	5.6 (4.7-6.3)			
Treatment intention	Curative	10.4 (7.6-14.0)	**<0.0001**	0.56 (0.45-0.71)	**<0.0001**
	Palliative	5.6 (4.7-6.3)			
Dose methodology	BSA/mBSA	6.3 (5.5-7.3)	**0.0308**		
	Partition model	7.4 (6.0-10.0)		0.78 (0.63-0.98)	**0.0314**
ALBI grade	1	7.9 (6.5-10.4)	**0.0162**		
	2	6.0 (5.5-7.2)		1.22 (0.96-1.55)	0.1057
	3	4.6 (2.6-8.6)		2.20 (1.23-3.92)	**0.0078**

Levels of significance: $p < 0.05$ (Log-rank test [Mantel-Haenszel version]).
ALBI: Albumin Bilirubin; BCLC Barcelona Clinic Liver Cancer; BSA: Body Surface Area; ECOG: Eastern Cooperative Oncology Group; NASH: Non-alcoholic steatohepatitis; TACE: Transcatheter Arterial Chemoembolization; TARE: Transarterial Radioembolization.

Table S4. Multivariable analysis for progression-free survival

Variable	Threshold	HR (95% CI)	p value
BCLC (vs A)	B	1.58 (1.06-2.34)	**0.025**
	C	1.99 (1.38-2.86)	**0.0002**
	D	1.34 (0.47-3.78)	0.5851
Cirrhosis (vs No)	Yes	1.31 (1.03-1.67)	**0.0312**
Tumour location (vs bilobar)	Left	0.78 (0.54-1.12)	0.1713
	Right	0.73 (0.57-0.92)	**0.0092**
Treatment intention (vs palliative)	Curative	0.55 (0.43-0.70)	**<.0001**
Levels of significance: p <0.05 (Cox proportional-hazards model). The proportional hazard function of the Cox model was verified. The following variables were considered in the multivariable model: Barcelona Clinic Liver Cancer; Eastern Cooperative Oncology Group; cirrhosis; ascites; tumour burden (nodules); location of tumour; extra-hepatic disease prior to transarterial radioembolization; prior ablation; prior chemotherapy; treatment intention; dose methodology; albumin-bilirubin grade. ALBI: Albumin-Bilirubin; BCLC Barcelona Clinic Liver Cancer; BSA: Body Surface Area; ECOG: Eastern Cooperative Oncology Group; TARE: Transarterial Radioembolization.			

Table S5. Multivariable analysis for hepatic progression free survival

Variable	Threshold	HR (95% CI)	p value
ECOG (vs 0)	1	1.32 (1.03-1.70)	**0.0274**
	2+3	1.22 (0.77-1.92)	0.4011
Cirrhosis (vs No)	Yes	1.43 (1.11-1.85)	**0.0060**
Location of tumour (vs Bilobar)	Left	0.80 (0.56-1.16)	0.2368
	Right	0.65 (0.51-0.83)	**0.0005**
Portal vein thrombosis (vs Patent)	Lobar	1.71 (1.15-2.56)	**0.0086**
	Main	1.53 (0.92-2.55)	0.1055
	Segmental	1.13 (0.85-1.50)	0.3865
Ablation (vs No)	Yes	0.78 (0.55-1.10)	0.1585
Abdominal radiotherapy (vs No)	Yes	0.50 (0.18-1.38)	0.1817
Treatment intention Curative (vs Palliative)	Curative	0.59 (0.46-0.75)	**<0.0001**

Levels of significance: $p < 0.05$ (Cox proportional-hazards model). The proportional hazard function of the Cox model was verified. The following variables were considered in the multivariable model: Barcelona Clinic Liver Cancer; Eastern Cooperative Oncology Group; cirrhosis; ascites; tumour burden (nodules); location of tumour; extra-hepatic disease prior to transarterial radioembolization; portal vein thrombosis; total bilirubin (mg/dl); prior ablation; prior transcatheter arterial chemoembolization; prior chemotherapy; treatment intention; dose methodology; albumin-bilirubin grade. ALBI: Albumin-Bilirubin; BCLC Barcelona Clinic Liver Cancer; BSA: Body Surface Area; ECOG: Eastern Cooperative Oncology Group; TARE: Transarterial Radioembolization.

Table S6. Propensity score matching

Variable	Type	BSA/mBSA (n=213)	Compartment Model (n=160)	All (n=373)*	P value
Cirrhosis	No	87 (40.8%)	26 (16.3%)	113 (30.3%)	**<.0001**
	Yes	126 (59.2%)	134 (83.8%)	260 (69.7%)	
Ascites	No	182 (85.4%)	141 (88.1%)	323 (86.6%)	0.4523
	Yes	31 (14.6%)	19 (11.9%)	50 (13.4%)	
Number of nodules	1	40 (18.8%)	80 (50.0%)	120 (32.2%)	**<.0001**
	2-5	71 (33.3%)	51 (31.9%)	122 (32.7%)	
	>5	52 (24.4%)	10 (6.3%)	62 (16.6%)	
	Uncountable	50 (23.5%)	19 (11.9%)	69 (18.5%)	
Location of tumour	Bilobar	106 (49.8%)	28 (17.5%)	134 (35.9%)	**<.0001**
	Left	22 (10.3%)	22 (13.8%)	44 (11.8%)	
	Right	85 (39.9%)	110 (68.8%)	195 (52.3%)	
Portal vein thrombosis	Patent	159 (74.6%)	95 (59.4%)	254 (68.1%)	**0.0163**
	Main	9 (4.2%)	8 (5.0%)	17 (4.6%)	
	Lobar	13 (6.1%)	17 (10.6%)	30 (8.0%)	
	Segmental	32 (15.0%)	40 (25.0%)	72 (19.3%)	
ALBI grade	1	91 (42.7%)	48 (30.0%)	139 (37.3%)	**0.0421**
	2	114 (53.5%)	105 (65.6%)	219 (58.7%)	
	3	8 (3.8%)	7 (4.4%)	15 (4.0%)	

Levels of significance: p <0.05 (Log-rank test [Mantel-Haenszel version]).
*Due to the presence of missing values for the ALBI grade, a total of 373 patients was considered for the analysis. ALBI: Albumin-Bilirubin; BSA: Body Surface Area;

Table S7. Comparing baseline characteristics

Variable	Type	BSA/mBSA	Partition model	All	P value
Age (year)	N :	245	177	422	0.1142
	Mean ± SD :	67.7 ± 10.9	66.0 ± 10.3	67.0 ± 10.7	
	(Min;Max) :	(22.0 ; 92.0)	(36.0 ; 91.0)	(22.0 ; 92.0)	
	Median [IQR] :	68.0 [62.0;74.0]	67.0 [59.0;75.0]	68.0 [60.0;74.0]	
Age (year)	<68	120 (49.0%)	90 (50.8%)	210 (49.8%)	0.7049
	≥68	125 (51.0%)	87 (49.2%)	212 (50.2%)	
Gender	Female	40 (16.3%)	34 (19.2%)	74 (17.5%)	0.7601
	Male	201 (82.0%)	140 (79.1%)	341 (80.8%)	
	Unknown	4 (1.6%)	3 (1.7%)	7 (1.7%)	
ECOG	0	123 (50.2%)	137 (77.4%)	260 (61.6%)	**<.0001**
	1	95 (38.8%)	36 (20.3%)	131 (31.0%)	
	2+3	27 (11.0%)	4 (2.3%)	31 (7.3%)	
Cirrhosis	No	94 (38.4%)	29 (16.4%)	123 (29.1%)	**<.0001**
	Yes	151 (61.6%)	148 (83.6%)	299 (70.9%)	
Cause of cirrhosis	Alcohol	51 (33.8%)	24 (16.2%)	75 (25.1%)	**<.0001**
	Hepatitis-B	17 (11.3%)	33 (22.3%)	50 (16.7%)	
	Hepatitis-C	36 (23.8%)	64 (43.2%)	100 (33.4%)	
	NASH	29 (19.2%)	9 (6.1%)	38 (12.7%)	
	Other	18 (11.9%)	18 (12.2%)	36 (12.0%)	
Ascites	No	205 (83.7%)	156 (88.1%)	361 (85.5%)	0.1983
	Yes	40 (16.3%)	21 (11.9%)	61 (14.5%)	
Number of nodules	1	47 (19.2%)	89 (50.3%)	136 (32.2%)	**<.0001**
	2-5	81 (33.1%)	57 (32.2%)	138 (32.7%)	
	>5	61 (24.9%)	11 (6.2%)	72 (17.1%)	
	Uncountable	56 (22.9%)	20 (11.3%)	76 (18.0%)	
Location of tumor	Bilobar	120 (49.0%)	30 (16.9%)	150 (35.5%)	**<.0001**
	Left	27 (11.0%)	24 (13.6%)	51 (12.1%)	
	Right	98 (40.0%)	123 (69.5%)	221 (52.4%)	
Extra-hepatic disease prior to treatment	No	219 (89.4%)	167 (94.4%)	386 (91.5%)	0.0717
	Yes	26 (10.6%)	10 (5.6%)	36 (8.5%)	
Portal vein thrombosis	Patent	178 (72.7%)	106 (59.9%)	284 (67.3%)	**0.0351**
	Main	11 (4.5%)	8 (4.5%)	19 (4.5%)	
	Lobar	19 (7.8%)	19 (10.7%)	38 (9.0%)	
	Segmental	37 (15.1%)	44 (24.9%)	81 (19.2%)	
BCLC stage	A	23 (9.4%)	36 (20.3%)	59 (14.0%)	**0.0011**
	B	140 (57.1%)	77 (43.5%)	217 (51.4%)	
	C	78 (31.8%)	64 (36.2%)	142 (33.6%)	
	D	4 (1.6%)	0 (0.0%)	4 (0.9%)	
Total bilirubin (mg/dl)	N :	243	176	419	**0.0148**
	Mean ± SD :	0.9 ± 0.5	1.0 ± 0.5	0.9 ± 0.5	
	(Min;Max) :	(0.1 ; 2.7)	(0.2 ; 3.0)	(0.1 ; 3.0)	
	Median [IQR] :	0.8 [0.5;1.1]	0.9 [0.6;1.2]	0.8 [0.5;1.2]	
Total bilirubin (mg/dl)	≤1.5	224 (92.2%)	153 (86.9%)	377 (90.0%)	0.0774
	>1.5	19 (7.8%)	23 (13.1%)	42 (10.0%)	
Albumin (g/dL)	N :	213	161	374	0.3003
	Mean ± SD :	3.7 ± 0.6	3.7 ± 0.5	3.7 ± 0.5	
	(Min;Max) :	(1.8 ; 5.1)	(2.0 ; 4.7)	(1.8 ; 5.1)	
	Median [IQR] :	3.8 [3.4;4.1]	3.7 [3.4;4.0]	3.7 [3.4;4.1]	

Bilirubin (µmol/L)	N :	243	176	419	**0.0117**
	Mean ± SD :	14.6 ± 7.9	16.6 ± 8.6	15.4 ± 8.3	
	(Min;Max) :	(2.0 ; 46.0)	(3.4 ; 52.0)	(2.0 ; 52.0)	
	Median [IQR] :	13.0 [8.6;18.8]	15.1 [10.1;21.0]	14.0 [9.1;20.0]	
ALBI grade	DM	32 (13.1%)	17 (9.6%)	49 (11.6%)	**0.0421**
	A1	91 (37.1%)	48 (27.1%)	139 (32.9%)	
	A2	114 (46.5%)	105 (59.3%)	219 (51.9%)	
	A3	8 (3.3%)	7 (4.0%)	15 (3.6%)	
Prior locoregional procedures	No	133 (54.3%)	100 (56.5%)	233 (55.2%)	0.6521
	Yes	112 (45.7%)	77 (43.5%)	189 (44.8%)	
Prior surgery	No	193 (78.8%)	157 (88.7%)	350 (82.9%)	**0.0075**
	Yes	52 (21.2%)	20 (11.3%)	72 (17.1%)	
Type of prior surgery	Liver surgery	50 (20.4%)	20 (11.3%)	70 (16.6%)	0.1112
	Liver transplant	2 (0.8%)	0 (0.0%)	2 (0.5%)	
Prior ablation	No	213 (86.9%)	149 (84.2%)	362 (85.8%)	0.4234
	Yes	32 (13.1%)	28 (15.8%)	60 (14.2%)	
Prior TACE	No	196 (80.0%)	131 (74.0%)	327 (77.5%)	0.1461
	Yes	49 (20.0%)	46 (26.0%)	95 (22.5%)	
Prior TACE	Conventional TACE	27 (11.0%)	38 (21.5%)	65 (15.4%)	**0.0099**
	Drug-Eluting TACE	19 (7.8%)	7 (4.0%)	26 (6.2%)	
	Other	3 (1.2%)	1 (0.6%)	4 (0.9%)	
Prior abdominal radiotherapy	No	239 (97.6%)	176 (99.4%)	415 (98.3%)	0.2469
	Yes	6 (2.4%)	1 (0.6%)	7 (1.7%)	
Other prior embolotherapies	No	242 (98.8%)	165 (93.2%)	407 (96.4%)	**0.0029**
	Yes	3 (1.2%)	12 (6.8%)	15 (3.6%)	
Prior chemotherapy	No	216 (88.2%)	165 (93.2%)	381 (90.3%)	0.0835
	Yes	29 (11.8%)	12 (6.8%)	41 (9.7%)	
Chemotherapy	Missing	2 (6.9%)	0 (0.0%)	2 (4.9%)	0.0891
	Other	0 (0.0%)	2 (16.7%)	2 (4.9%)	
	Sorafenib	27 (93.1%)	10 (83.3%)	37 (90.2%)	
Treatment intention	Ablation	3 (1.2%)	14 (7.9%)	17 (4.0%)	**<.0001**
	Bridge to surgery or transplant	17 (6.9%)	9 (5.1%)	26 (6.2%)	
	Down-sizing/down-staging	60 (24.5%)	77 (43.5%)	137 (32.5%)	
	Palliative	165 (67.3%)	77 (43.5%)	242 (57.3%)	
Treatment intention	Curative	80 (32.7%)	100 (56.5%)	180 (42.7%)	**<.0001**
	Palliative	165 (67.3%)	77 (43.5%)	242 (57.3%)	
Prescribed activity whole liver treatment (GBq)	N :	71	114	185	**0.0102**
	Mean ± SD :	1.5 ± 0.7	1.3 ± 0.6	1.4 ± 0.6	
	(Min;Max) :	(0.7 ; 5.0)	(0.2 ; 3.0)	(0.2 ; 5.0)	
	Median [IQR] :	1.5 [1.2;1.8]	1.3 [0.8;1.7]	1.4 [1.0;1.8]	
Prescribed activity whole liver treatment (GBq)	≤0.993	12 (4.9%)	35 (19.8%)	47 (11.1%)	0.1048
	0.993-1.4	19 (7.8%)	33 (18.6%)	52 (12.3%)	
	1.4-1.76	20 (8.2%)	20 (11.3%)	40 (9.5%)	
	>1.76	20 (8.2%)	26 (14.7%)	46 (10.9%)	
	N :	174	63	237	**0.0089**

Prescribed activity left lobe treatment (GBq)	Mean ± SD :	0.4 ± 0.5	0.3 ± 0.6	0.4 ± 0.5	
	(Min;Max) :	(0.0 ; 2.0)	(0.0 ; 3.0)	(0.0 ; 3.0)	
	Median [IQR] :	0.3 [0.0;0.7]	0.0 [0.0;0.4]	0.0 [0.0;0.7]	
Prescribed activity left lobe treatment (GBq)	≤0	85 (34.7%)	47 (26.6%)	132 (31.3%)	**0.0002**
	0-0.67	44 (18.0%)	3 (1.7%)	47 (11.1%)	
	>0.67	45 (18.4%)	13 (7.3%)	58 (13.7%)	
Prescribed activity right lobe treatment (GBq)	N :	174	63	237	0.4999
	Mean ± SD :	1.1 ± 0.7	1.0 ± 0.9	1.1 ± 0.8	
	(Min;Max) :	(0.0 ; 5.5)	(0.0 ; 3.5)	(0.0 ; 5.5)	
	Median [IQR] :	1.1 [0.8;1.4]	1.0 [0.4;1.5]	1.1 [0.7;1.4]	
Prescribed activity right lobe treatment (GBq)	≤0.666	36 (14.7%)	25 (14.1%)	61 (14.5%)	**0.0031**
	0.666-1.1	50 (20.4%)	10 (5.6%)	60 (14.2%)	
	1.1-1.43	48 (19.6%)	9 (5.1%)	57 (13.5%)	
	>1.43	40 (16.3%)	19 (10.7%)	59 (14.0%)	
APRI	N :	203	172	375	0.7277
	Mean ± SD :	1.1 ± 1.2	1.2 ± 1.4	1.1 ± 1.3	
	(Min;Max) :	(0.1 ; 10.1)	(0.1 ; 14.7)	(0.1 ; 14.7)	
	Median [IQR] :	0.8 [0.4;1.4]	0.8 [0.4;1.4]	0.8 [0.4;1.4]	
APRI	Missing	42 (17.1%)	5 (2.8%)	47 (11.1%)	0.8163
	≤0.40	44 (18.0%)	39 (22.0%)	83 (19.7%)	
	>0.40	159 (64.9%)	133 (75.1%)	292 (69.2%)	

Levels of significance: p <0.05 (Log-rank test [Mantel-Haenszel version]).
ALBI: Albumin-Bilirubin; APRI: Aspartate aminotransferase-to-platelet ratio index; BCLC Barcelona Clinic Liver Cancer; (m)BSA: (modified) Body Surface Area; ECOG: Eastern Cooperative Oncology Group; GBQ: Giga-becquerel; INR: International Normalized Ratio; IQR: Interquartile Range; NASH: Non-alcoholic steatohepatitis; TACE: Transcatheter Arterial Chemoembolization;

Table S8. The effect of centre expertise in survival outcomes between the BSA/mBSA and the partition model cohorts.

The hypothesis is that centres that perform partition model dosimetry are generally better-performing hospitals, because partition model dosimetry requires a certain level of expertise and infrastructure dedicated to TARE. If this hypothesis would be true, we would expect better OS/PFS/hPFS outcomes of the BSA/mBSA patients that are treated in those hospitals.

In the below table we compared the survival outcomes of the following three groups: 1) partition model dosimetry patients; 2) dose calculation with BSA/mBSA for patients that were treated in sites that also performed partition model dosimetry; and 3) dose calculation with BSA/mBSA for patients that were treated in sites that did not perform partition model dosimetry.

Outcome	Group	No. of patients	Median (95% CI)	p value	HR (95% CI)	p value HR
**OS**	1	177	23.4 (18.3-38.9)	<0.0001	0.50 (0.38-0.67)	0
	2	73	15.3 (8.7-20.3)		0.84 (0.60-1.18)	0.3111
	3	172	13.3 (11.5-15.0)			
**PFS**	1	177	7.4 (5.9-10.0)	0.005	0.68 (0.54-0.87)	0.0016
	2	73	5.5 (3.5-6.9)		0.93 (0.69-1.25)	0.6247
	3	171	5.7 (5.1-7.0)			
**hPFS**	1	177	7.4 (6.0-10.0)	0.0955	0.78 (0.61-0.99)	0.0397
	2	73	5.9 (4.0-7.7)		0.97 (0.72-1.32)	0.855
	3	172	6.8 (5.6-7.7)			

Levels of significance: p <0.05 (Log-rank test [Mantel-Haenszel version]).
1. Patients with partition model
2. Patients with BSA/mBSA in sites that also performed partition model
3. Patients with BSA/mBSA in sites that did not perform partition model

This table shows that BSA group 2 did not benefit from being treated at a site that also performed partition model dosimetry. In fact, when we compare only the 2 groups with BSA/mBSA we see that there are no significant differences between the outcomes of the groups, suggesting that dose calculation with BSA leads to similar outcomes, regardless of the expertise and infrastructure of the centre.

Outcome	HR (95% CI)	p value HR
OS	0.85 (0.61-1.18)	0.3326
PFS	0.94 (0.69-1.27)	0.6684
hPFS	0.98 (0.72-1.33)	0.9

Levels of significance: p <0.05 (Log-rank test [Mantel-Haenszel version]).

Table S9. Toxicities.

A: Short-term safety data

Category	Subcategory	HCC (n=422)
Longest hospital stay	n[a]	323
	24 to <36 hours	57 (13.5%)
	36 to <48 hours	42 (10.0%)
	48 to <72 hours	186 (44.1%)
	> or = 72 hours	38 (9.0%)
**Severe day of treatment complications**		
Severe abdominal pain	Yes	3 (0.7%)
Severe abdominal pain	Yes: Grade 3	3 (0.7%)
	Yes: Grade 4	
Severe vomiting	Yes	2 (0.5%)
Severe vomiting	Yes: Grade 3	2 (0.5%)
Vascular	Yes	3 (0.7%)
Vascular	Yes: Major	1 (0.2%)
	Yes: Minor	2 (0.5%)
Severe other	Yes	4 (0.9%)
**Time till death**		
n[a]	422	n[a]
Death within 30 days	3 (0.7%)	Within 30 days
Death between 31 and 60 days	7 (1.7%)	Between 31 and 60 days
Death between 61 and 90 days	15 (3.6%)	Between 61 and 90 days
Lost to follow-up within 90 days	47 (11.1%)	Lost to follow-up within 90 days

B: Adverse events

Category	Subcategory	HCC (n=422)
Patients with adverse events	Number of patients with at least one adverse event	155 (36.7%)
Adverse events (all)	Abdominal pain	60 (14.2%)
	Fatigue	65 (15.4%)
	Fever	25 (5.9%)
	Nausea	37 (8.8%)
	Vomiting	22 (5.2%)
	Gastrointestinal ulceration	3 (0.7%)
	Gastritis	3 (0.7%)
	Radiation cholecystitis	
	Radiation pancreatitis	
	Radioembolisation-induced liver disease	6 (1.4%)
	Other	90 (21.3%)
Adverse events grade 3-4	Abdominal pain	9 (2.1%)
	Fatigue	6 (1.4%)
	Fever	2 (0.5%)
	Nausea	3 (0.7%)
	Vomiting	2 (0.5%)
	Gastrointestinal ulceration	1 (0.2%)
	Gastritis	

	Radiation cholecystitis	
	Radioembolisation-induced liver disease	1 (0.2%)
	Other	14 (3.3%)
Adverse events grade 5	Radioembolisation-induced liver disease	2 (0.5%)
	Other	3 (0.7%)
All adverse events	Number of adverse events	646
	Person years	197.05
	Incidence (95% CI)	3.28 (3.278 - 3.279)
Adverse events of special interest	Number of adverse events	399
	Person years	126.89
	Incidence (95% CI)	3.14 (3.144 - 3.145)
Time without toxicity	Time without toxicity (95% CI)	13.8 months (11.1 - 24.0)

C: Abnormal lab values

**Category**	**Subcategory**	**HCC (n=422)**
Patients with abnormal lab values	No. of patients with at least one abnormal lab	254 (60.2%)
Abnormal lab values	Hypoalbuminemia	126 (29.9%)
	Hyperbilirubinemia	124 (29.4%)
	ALT increased	109 (25.8%)
	AST increased	154 (36.5%)
	INR increased	89 (21.1%)
	Neutrophil count decreased	16 (3.8%)
	Platelet count decreased	137 (32.5%)
	Other	101 (23.9%)
ALT: Alanine Aminotransferase; AST: Aspartate Transaminase; INR: International Normalized Ratio		
