# Supplementary material for: Factors impacting survival after transarterial radioembolization in patients with hepatocellular carcinoma: Results from the prospective CIRT study
Source: JHEP Rep. 2022 Nov 25;5(2):100633. doi: 10.1016/j.jhepr.2022.100633 (PMC9804139; doi:10.1016/j.jhepr.2022.100633)
Supplement: Multimedia component 4 [file mmc4.pdf]

Research article JHEP|Reports

Factors impacting survival after transarterial radioembolization in patients with hepatocellular carcinoma: Results from the prospective CIRT study

Frank Kolligs,[1] Dirk Arnold,[2] Rita Golfieri,[3] Maciej Pech,[4] Bora Peynircioglu,[5] Thomas Pfammatter,[6] Maxime Ronot,[7] Bruno Sangro,[8] Niklaus Schaefer,[9] Geert Maleux,[10] Graham Munneke,[11] Helena Pereira,[12,15] Bleranda Zeka,[13] Niels de Jong,[13,*] Thomas Helmberger [14], on behalf of the CIRT Principal Investigators

[1]*Department of Internal Medicine and Gastroenterology, Helios Klinikum Berlin-Buch, Berlin, Germany;* [2]*Oncology and Hematology, Asklepios Tumorzentrum Hamburg, AK Altona, Hamburg, Germany;* [3]*Department of Radiology, IRCCS Azienda Ospedaliero-Universitaria di Bologna, Bologna, Italy;* [4]*Department of Radiology and Nuclear Medicine, University of Magdeburg, Magdeburg, Germany;* [5]*Department of Radiology, School of Medicine, Hacettepe University, Sihhiye Campus, Ankara, Turkey;* [6]*Institute of Diagnostic and Interventionals Radiology, Universitätsspital Zürich, Zürich, Switzerland;* [7]*Université Paris Cité, Paris & Service de Radiologie, APHP Nord, Hôpital Beaujon, Clichy, France;* [8]*Liver Unit and HPB Oncology Area, Clínica Universidad de Navarra and CIBEREHD, Pamplona, Spain;* [9]*Service de médecine nucléaire et imagerie moléculaire, CHUV, Centre Hospitalier Universitaire Vaudois, Lausanne, Switzerland;* [10]*Radiology, Universitair Ziekenhuis Leuven, Leuven, Belgium;* [11]*Interventional Oncology, University College London Hospitals NHS Foundation Trust, London, United Kingdom;* [12]*Assistance Publique-Hôpitaux de Paris, Hôpital Européen Georges-Pompidou, Unité de Recherche Clinique, Paris, France;* [13]*Clinical Research Department, Cardiovascular and Interventional Radiological Society of Europe, Vienna, Austria;* [14]*Department of Radiology, Neuroradiology and Minimal-Invasive Therapy, Klinikum Bogenhausen, Munich, Germany;* [15]*INSERM, Centre d'Investigation Clinique 1418 (CIC1418), Paris, France*

JHEP Reports **2023.** https://doi.org/10.1016/j.jhepr.2022.100633

Background & Aims: Transarterial radioembolization (TARE) with Yttrium-90 resin microspheres is an established treatment option for patients with hepatocellular carcinoma (HCC). However, optimising treatment application and patient selection remains challenging. We report here on the effectiveness, safety and prognostic factors, including dosing methods, associated with TARE for HCC in the prospective observational CIRT study.
Methods: We analysed 422 patients with HCC enrolled between Jan 2015 and Dec 2017, with follow-up visits every 3 months for up to 24 months after first TARE. Patient characteristics and treatment-related data were collected at baseline; adverse events and time-to-event data (overall survival [OS], progression-free survival [PFS] and hepatic PFS) were collected at every 3-month follow-up visit. We used the multivariable Cox proportional hazard model and propensity score matching to identify independent prognostic factors for effectiveness outcomes.
Results: The median OS was 16.5 months, the median PFS was 6.1 months, and the median hepatic PFS was 6.7 months. Partition model dosimetry resulted in improved OS compared to body surface area calculations on multivariable analysis (hazard ratio 0.65; 95% CI 0.46-0.92; p = 0.0144), which was confirmed in the exact matching propensity score analysis (hazard ratio 0.56; 95% CI 0.35-0.89; p = 0.0136). Other independent prognostic factors for OS were ECOG-performance status >0 (p = 0.0018), presence of ascites (p = 0.0152), right-sided tumours (p = 0.0002), the presence of portal vein thrombosis (p = 0.0378) and main portal vein thrombosis (p = 0.0028), ALBI grade 2 (p = 0.0043) and 3 (p = 0.0014). Adverse events were recorded in 36.7% of patients, with 9.7% of patients experiencing grade 3 or higher adverse events.
Conclusions: This large prospective observational dataset shows that TARE is an effective and safe treatment in patients with HCC. Using partition model dosimetry was associated with a significant improvement in survival outcomes.
Impact and implications: Transarterial radioembolization (TARE) is a form of localised radiation therapy and is a potential treatment option for primary liver cancer. We observed how TARE was used in real-life clinical practice in various European countries and if any factors predict how well the treatment performs. We found that when a more complex but personalised method to calculate the applied radiation activity was used, the patient responded better than when a more generic method was used. Furthermore, we identified that general patient health, ascites and liver function can predict outcomes after TARE.
Clinical trial number: NCT02305459.
© 2022 The Author(s). Published by Elsevier B.V. on behalf of European Association for the Study of the Liver (EASL). This is an open access article under the CC BY license (http://creativecommons.org/licenses/by/4.0/).

Keywords: SIRT; observational; liver; radioembolization; dosimetry; registry.
Received 11 May 2022; received in revised form 14 November 2022; accepted 16 November 2022; available online 25 November 2022
* Corresponding author. Address: Clinical Research Department, Cardiovascular and Interventional Radiological Society of Europe, Neutorgasse 9, 1010, Vienna Austria; Tel.: +43 1904200347.
E-mail address: dejong@cirse.org (N. de Jong).

Introduction

Hepatocellular carcinoma (HCC) is the most frequent primary liver cancer and represents the third most common cause of cancer-related deaths worldwide.[1,2] The most critical risk factor for the development of HCC is cirrhosis. Overall, the prognosis of

ELSEVIER

EASL The Home of Hepatology

HCC is poor, with a life expectancy of 6-38 months, depending on the Barcelona Clinic Liver Cancer (BCLC) stage.[3] Only a minority of patients are eligible for curative-intent treatments, including surgical resection, liver transplantation, and ablative therapies.[4–9] In intermediate stages, transcatheter arterial chemoembolization (TACE) is standard of care; systemic treatments such as sorafenib, lenvatinib, and a combination of atezolizumab and bevacizumab have been approved for the first-line medical treatment of advanced and metastatic HCC based on convincing phase III trials.[10–13]

Guidelines for the treatment of HCC also propose transarterial radioembolization (TARE, also known as selective internal radiation therapy [SIRT]) as an optional treatment modality for patients with liver dominant disease not eligible for surgical or ablative therapies, or who experienced no response, significant side effects or intolerance when treated with systemic therapies.[4,6–9] TARE is an interventional therapeutic procedure that involves the targeted delivery of high doses of radiation to liver tumours via the hepatic artery. Several studies have shown that TARE has a favourable safety profile and displays promising results in terms of local tumour control in patients with unresectable HCC limited to the liver in the intermediate and advanced stages.[14–18] Despite this, recent randomised controlled trials on TARE in HCC showed that compared to sorafenib alone, TARE or TARE plus sorafenib as a first-line treatment option for patients with unresectable HCC did not improve overall survival (OS) or progression-free survival (PFS) in these patient cohorts.[19,20] While questions have been raised regarding discrepancies in patient inclusion and site experience in administering TARE, which may have influenced outcomes,[21] recent research into dosimetry methods suggests that improving dose calculation and delivery could improve survival outcomes.[22] The prospective randomised DOSISPHERE-01 trial showed significantly better overall survival results with a personalised dosimetry model than the standard dose calculation model using glass microspheres in patients with unresectable, locally advanced HCC.[23,24] This suggests that further optimising selection of patients, treatment application and the dosimetry models may improve survival outcomes of patients with HCC.

The Cardiovascular and Interventional Radiological Society of Europe (CIRSE) initiated a European-wide observational study on the clinical application and outcomes of TARE with Y90 resin microspheres (SIR-Spheres® Y-90 resin microspheres, Sirtex Medical Pty Limited; St. Leonards, NSW, Australia). The study (NCT02305459) was open to all indications and recruited one of the largest cohorts on TARE in liver malignancies to date.[25] The objective of the current subgroup analysis was to investigate factors influencing survival in patients with HCC treated with TARE, including the effect that methods to calculate the prescribed dose have on effectiveness outcomes. The primary endpoint was OS, while secondary endpoints were PFS, PFS in the liver only (hepatic PFS [hPFS]), safety, and identification of potential prognostic survival factors, including an evaluation of the impact of methods to calculate the prescribed activity on survival outcomes.

Patients and methods

Study design

We analysed 422 patients with HCC collected in the CIRSE Registry for SIR-Spheres Therapy (CIRT) study. CIRT is a prospective, single device, multi-centre observational study of patients with primary and metastatic hepatic malignancies treated with TARE using Y90 resin microspheres as the standard of care. The CIRT methodology was published by Helmberger *et al.*[26] Sites were invited to participate if they had at least 40 TARE cases and 10 cases in 12 months prior to invitation. In total, 27 participating sites in eight countries were identified and enrolled from April 2014 until April 2017, of which 25 sites included patients with HCC.[25]

Data was collected using a customised electronic data capturing system and electronic case report form that was developed by ConexSys Inc (Lincoln, RI, United States) and hosted on a local secure server in Vienna, Austria maintained by ITEA (Vienna, Austria). Statistical analyses were performed in SAS 9.4 (SAS Institute, Cary, NC, USA) and RStudio under R4.0.0 (R Foundation, Vienna, Austria, Supplementary CTAT Table).

Patient selection

Patients included in the analysis were adults diagnosed with HCC and scheduled to receive TARE with Y90 resin microspheres. There were no specific inclusion or exclusion criteria. The indication for TARE, the treatment design, the methods used for dose calculation and the follow-up regimen were based on the centres' internal standards. Participating sites contractually agreed to include all eligible patients consecutively. All included patients signed an informed consent form. This research project was performed in accordance with the ethical standards of the applicable institutional and/or national ethics committees and with the 1964 Helsinki declaration and its later amendments or comparable ethical standards.

Patient recruitment took place between 1 January 2015 and 31 December 2017. Follow-up data were collected until 31 December 2019. Sites were requested to follow-up with the patient every 3 months up to 24 months after the first TARE treatment. In addition, sites were encouraged to obtain follow-up information from referring physicians if follow-up evaluations were not performed at the site of the TARE treatment.

Assessments

At the time of first treatment, baseline data, demographics and treatment-related data were collected. Information concerning post-TARE treatments, safety data and time-to-event data were gathered at every follow-up visit. Time-to-event was defined from the date of the first TARE treatment until the date of the event. Liver function was described using the albumin-bilirubin (ALBI) formula developed by Johnson *et al.*: ALBI score = ($\log^{10}$ bilirubin [μmol/L] × 0.66) + (albumin [g/L] × −0.0852). ALBI score ≤−2.60 is grade 1, >−2.60 to ≤−1.39 is grade 2, and >−1.39 is grade 3.[27] BCLC classifications were determined at the sites, but all classifications were evaluated according to the uniform BCLC staging standards set out by Reig *et al.* in the recent (2022) update.[28] Where necessary, patients were re-classified. Information on whether portal vein thrombosis (PVT) was malignant was not collected, but lobar and main PVT were considered malignant, while segmental PVT was considered malignant if the site classified the patient as BCLC C. Safety outcomes are described as severe day-of-treatment complications and occurrences of any adverse events after treatment, according to the Common Terminology Criteria for Adverse Events, version 4.03. Pre-defined serious adverse events (grade 3 and 4) were abdominal pain, fatigue, fever, nausea, vomiting, gastrointestinal ulceration,

gastritis, radiation cholecystitis, radiation pancreatitis and radioembolization-induced liver disease (REILD). An open text field allowed us to collect details on other serious adverse events.

Statistical analysis

Data are presented as mean ± SD or median (IQR) for continuous variables and number (%) for categorical variables. Percentages are based on the whole cohort (N = 422) unless otherwise indicated. Patients who died during the study were categorized as having progression for the purpose of PFS and hPFS analysis. Patients alive and progression-free were censored on the day of last follow-up. The simultaneous occurrence of hepatic progression and extrahepatic progression was considered as hepatic progression.

Comparisons between groups were performed using the log-rank test (Mantel-Haenszel version). The median OS, PFS and hPFS times were calculated with their associated 95% CIs. The group effect was calculated with a Cox proportional-hazards model with hazard ratio (HR) and 95% CIs. The development over time of the ALBI, bilirubin, albumin and international normalized ratio (INR) values were explored using a linear mixed model.

A multivariable analysis for OS, PFS and hPFS was performed using a Cox proportional-hazards model whereby the selection of variables was determined following a univariable analysis and a stepwise variable selection procedure, with a significance level of 0.2 used to determine whether to enter a predictor into the stepwise model. The model with the lowest Akaike information criterion value was considered the final model. All available data were used, and no imputations of missing data were made.

Additional analyses were performed to evaluate the impact on OS, PFS and hPFS of the two main methods to calculate the prescribed Y90 activity: partition model and (modified) body surface area ([m]BSA) methodology. For the comparison of partition model dosimetry (n = 177) with BSA/mBSA (n = 245), we considered a locally modified version of the partition model (n = 3) and voxel-based dosimetry (n = 1) as following the partition model. To compare the two groups, a propensity score analysis was performed. The propensity score is the probability of treatment assignment conditional on measured baseline covariates. Two approaches were used for the propensity score:

1. Matching: Greedy nearest neighbour matching within a calliper of 0.2 of the propensity score was used. Using this approach, a patient treated with partition model dosimetry is selected. This treated patient is then matched with a patient treated based on the BSA activity calculation, whose propensity score is closest to that of the treated patient, subject to the constraint that the differences between their propensity scores are less than a specified maximum (the calliper distance). To estimate the marginal treatment effect for OS, PFS and hPFS, a Cox model with a robust variance estimator that accounts for clustering within matched pairs was used.
2. Inverse probability of treatment weighting (IPTW): IPTW using the propensity score uses weights based on the propensity score to create a synthetic sample in which the distribution of measured baseline covariates is independent of treatment assignment.

To obtain appropriate estimates of variance, stabilised weights were used. For each patient, the stabilised weight is calculated by multiplying his or her original weight by the proportion of patients who received the treatment that he or she received. A Cox model, adjusted for stabilised weights, was used to estimate the relative treatment effect for OS, PFS, and hPFS.

A standardised difference between the two groups for each patient characteristic of interest was calculated to assess whether the covariates are well balanced between the partition model and BSA/mBSA models. The balance between groups was achieved if the magnitude of the standardised difference was less than 0.25.

Results

Patient demographics

Four hundred and twenty-two patients with HCC from 25 centres in eight countries were included in this study (Table 1). The median follow-up time was 11.1 months, and 116/422 (27.5%) patients were censored before 24 months due to lack of follow-up information. The mean age of our cohort was 67 years and 341/422 (80.8%) patients were male. In general, the patient population was representative for TARE, with 115/422 (27.3%) patients in BCLC stage B and 247/422 (58.5%) in BCLC stage C; Eastern Cooperative Oncology Group (ECOG) performance status 0 (260/422, 61.6%) or 1 (131/422, 31.0%); and a preserved liver function with ALBI grade 1 (139/422, 32.9%) or 2 (219/422, 51.9%). Cirrhosis was found in 299/422 (70.9%) patients and ascites in 61/422 (14.5%). Thirty-two percent (136/422, 32.2%) had a single tumour nodule, 138/422 (32.7%) had two to five nodules and 72/422 (17.1%) had more than five tumour nodules. Data on exact tumour size was not collected.

Bilobar disease was observed in 35.5% (150/422) of patients, while unilobar disease was primarily right-sided (52.4%, 221/422). Extrahepatic disease was diagnosed in 36/422 (8.5%) patients and PVT was present in 138/422 (32.7%). Prior to TARE, 95/422 (22.5%) patients had been treated with TACE, 72/422 (17.1%) with surgery and 60/422 (14.2%) with percutaneous ablation. Prior systemic therapy was performed in 41/422 (9.7%) patients (sorafenib 37/41, 90.2%).

Treatment and follow-up

BSA or mBSA were used to determine the prescribed Y90 activity (165/422 [39.1%] and 80/422 [19.0%], respectively) in most patients (Table 2). Partition model dosimetry was used in 177/422 (41.9%) cases. Whole-liver treatment was performed in 123/422 (29.1%) patients, compared to therapies directed to the right lobe, left lobe, or liver segment (177/422 [41.9%], 56/422 [13.3%], 66/422 [15.6%], respectively). The median prescribed activity was 1.40 GBq for whole-liver treatments (IQR 0.99–1.76), 1.20 GBq for right lobe treatments (IQR 1.00–1.49) and 0.73 (IQR 0.54–1.08) for left lobe treatments. Seventy (70/422 [16.5%]) patients had two or more treatment sessions. Treatments after TARE are listed in Table S1. An increase in ALBI, bilirubin and INR, and a decrease in albumin values was observed 3 months after treatment (p <0.0001, Fig. S1).

Effectiveness

The median OS was 16.5 months (95% CI 14.2-19.3), median PFS was 6.1 months (95% CI 5.7-7.0), and median hPFS was 6.7 months for the entire population (95% CI 5.9-7.6). Survival was highest in patients with BCLC A (41.4 months; 95% CI 22.5-ND; p <0.0001) (Table 3). The subgroup of patients with ALBI grade 1 lived longer (21.1 months; 95% CI 19.2-28.8; p <0.0001) than those with higher ALBI grades (grade 2: 14.0 months; 95% CI 11.5-16.5; p = 0.0005; grade 3: 7.8 months; 95% CI 2.7-12.9; p

Table 1. Baseline patient characteristics.

Category	Subcategory	HCC (N = 422)
Sex	n (%)	415 (98.3)
	Male	341 (80.8)
	Female	74 (17.5)
Age (years)	n (%)	422 (100)
	Mean ± SD (range)	67.0 ± 10.7 (22-92)
	Median (IQR)	68 (60–74)
ECOG performance status	n (%)	422 (100)
	0	260 (61.6)
	1	131 (31.0)
	2	31 (7.4)
Cirrhosis	n (%)	422 (100)
	Yes	299 (70.9)
	No	123 (29.1)
Ascites	n (%)	422 (100)
	Yes	61 (14.5)
	No	361 (85.5)
Number of nodules	n (%)	422 (100)
	1	136 (32.2)
	2–5	138 (32.7)
	>5	72 (17.1)
	Uncountable	76 (18.0)
Location of tumour	n (%)	422 (100)
	Bilobar	150 (35.5)
	Left only	51 (12.1)
	Right only	221 (52.4)
Extrahepatic metastases	n (%)	422 (100)
	Yes	36 (8.5)
	No	386 (91.5)
Portal vein thrombosis	n (%)	422 (100)
	Patent	284 (67.3)
	Segmental	81 (19.2)
	Lobar	38 (9.0)
	Main	19 (4.5)
BCLC stage	n (%)	422 (100)
	A	54 (12.8)
	B	115 (27.3)
	C	247 (58.5)
	D	6 (1.4)
ALBI grade	n (%)	373 (88.4)
	1	139 (32.9)
	2	219 (51.9)
	3	15 (3.6)
Prior locoregional procedures	n[a] (%)	233 (55.2)
	Surgery	72 (17.1)
	Percutaneous ablation	60 (14.2)
	TACE	95 (22.5)
	Abdominal radiotherapy	7 (1.7)
	Vascular procedure	15 (3.6)
Prior systemic therapies	n (%)	41 (9.7)
	Sorafenib	37 (8.7)
	Other	4 (0.9)
Intention of TARE	n (%)	422 (100)
	Ablation	17 (4.0)
	Bridge to surgery or transplant	26 (6.2)
	Downsizing	137 (32.5)
	Palliative	242 (57.3)
Bilirubin (mg/dl)	n (%)	419 (99.3)
	Mean ± SD	0.90 ± 0.49
	Median (IQR)	0.82 (0.53–1.17)
	>1.5 mg/dl	42 (10)
Albumin (g/dl)	n (%)	373 (88.4)
	Mean ± SD	3.72 ± 0.54
	Median (IQR)	3.7 (3.4–4.1)
	<3.5 g/dl	104 (24.6)
INR	n (%)	340 (80.6)
	Mean ± SD	1.14 ± 0.21
	Median (IQR)	1.1 (1.04-1.19)
	>1.2	66 (15.6)

(*continued on next page*)

Table 1 (*continued*)

Category	Subcategory	HCC (N = 422)
ALT (U/L)	n (%)	393 (93.1)
	Mean ± SD	43.48 (30.20)
	Median (IQR)	34.5 (23–55.5)
Creatinine (mg/dl)	n (%)	419 (99.3)
	Mean ± SD	0.99 (0.70)
	Median (IQR)	0.87 (0.74–1.06)

ALBI, albumin-bilirubin; ALT, alanine aminotransferase; BCLC, Barcelona Clinic Liver Cancer; ECOG, Eastern Cooperative Oncology Group; INR, international normalized ratio; TACE, transcatheter arterial chemoembolization; TARE, transarterial radioembolization.
[a] Patients can have multiple prior locoregional procedures.

Table 2. Treatment-associated parameters.

Category	Subcategory	HCC (N = 422)
Activity administered (GBq)	n (%)	422 (100)
	Whole liver (median; IQR; range)	1.40; 0.99-1.76; 0.18-5
	Right lobe (median; IQR; range)	1.20; 1.00-1.49; 0.37-5.50
	Left lobe (median; IQR; range)	0.73; 0.54-1.08; 0.20-3.00
Target treatment	n (%)	422 (100)
	Whole liver	123 (29.1)
	Right lobe	177 (41.9)
	Left lobe	56 (13.3)
	Segmental	66 (15.6)
Target tumour volume (ml)	n (%)	393 (93.1)
	Median; IQR; range	142; 53-341; 3-3642
Target liver volume (ml)	n (%)	396 (93.8)
	Median; IQR; range	1,663; 1,356-2,100; 281-5,460
Number of treatments	n (%)	422 (100)
	1	354 (83.9)
	2	66 (15.6)
	3 or more	4 (0.9)
Dose methodology	n (%)	422 (100)
	BSA	165 (39.1)
	Modified BSA	80 (19.0)
	Partition model	177 (41.9)
Embolization before treatment	n (%)	355 (84.1)
	Yes	138 (32.7)
	No	217 (51.4)

BSA, body surface area; HCC, hepatocellular carcinoma.

<0.0001). The number of tumour nodules, tumour location, presence of extrahepatic metastases, and PVT were also associated with survival (Table 3), as well as dose methodology (Fig. 1). If the prescribed activity was determined by the BSA/mBSA method, median OS was 13.4 months (95% CI 11.5-16.1) as compared to the partition model with a median OS of 23.4 months (95% CI 18.3-38.9; p <0.0001, see below for the subgroup analysis). The analysis of PFS and hPFS according to covariates is shown in Tables S2 and S3.

The multivariable analysis showed that, after controlling for the other variables, statistically significant variables predicting overall survival were ECOG >0 (ECOG 1: HR 1.64; 95% CI 1.20-2.23; p = 0.0018), presence of ascites (HR 1.62; 95% CI 1.10-2.38; p = 0.0152), cirrhosis (HR 1.39; 95% CI 1.00-1.194), extrahepatic disease (HR 1.55; 95% CI 1.01-2.27) and PVT (segmental PVT: HR 1.46; 95% CI 1.02-2.10; p = 0.0378; and main PVT: HR 2.49; 95% CI 1.37-4.54; p = 0.0028), right-sided tumours (HR 0.56; 95% CI 0.41-0.76; p = 0.0002), ALBI grade >1 (grade 2: HR 1.56; 95% CI 1.15-2.12;

Table 3. Univariable analysis for overall survival.

Variable	Threshold	Median (95% CI)	*p* value	HR (95% CI)	*p* value HR
Age (year)	<68	17.2 (14.0-20.3)	0.4356		
	≥68	15.2 (12.2-19.3)		1.11 (0.86-1.42)	0.4340
Sex	Female	18.7 (14.4-23.0)	0.3421	0.85 (0.60-1.20)	0.3444
	Male	15.4 (12.9-19.3)			
ECOG	0	20.7 (18.6-24.4)	**<0.0001**		
	1	12.6 (10.1-14.9)		1.95 (1.49-2.56)	**<0.0001**
	2 + 3	8.1 (6.2-11.4)		2.98 (1.87-4.76)	**<0.0001**
Cirrhosis	No	19.2 (15.0-22.9)	0.1078		
	Yes	14.9 (12.5-19.2)		1.27 (0.95-1.69)	0.1093
Cause of cirrhosis	Alcohol	10.4 (7.9-14.0)	**0.0012**		
	Hepatitis B	20.4 (12.2-ND)		0.51 (0.32-0.83)	**0.0060**
	Hepatitis C	19.7 (13.3-30.2)		0.51 (0.34-0.75)	**0.0007**
	NASH	11.6 (6.7-14.9)		0.97 (0.61-1.53)	0.8829
	Other	20.8 (11.2-23.0)		0.59 (0.36-0.96)	**0.0335**
Ascites	No	18.3 (15.3-20.4)	**0.0009**		
	Yes	9.9 (5.6-14.0)		1.75 (1.25-2.44)	**0.0010**
Number of nodules	1	20.8 (15.4-28.8)	**0.0009**		
	2-5	19.2 (14.0-22.9)		1.33 (0.96-1.84)	0.0851
	>5	13.1 (10.3-18.2)		1.66 (1.14-2.42)	**0.0081**
	Uncountable	10.7 (7.2-12.9)		2.05 (1.42-2.97)	**0.0001**
Location of tumour	Bilobar	11.6 (8.4-16.0)	**0.0001**		
	Left	14.4 (10.3-30.2)		0.65 (0.43-0.99)	**0.0422**
	Right	20.8 (16.5-23.4)		0.55 (0.42-0.73)	**<0.0001**
Extrahepatic disease prior to treatment	No	17.2 (14.4-20.3)	**0.0033**		
	Yes	10.4 (6.8-12.7)		1.81 (1.21-2.71)	**0.0037**
Portal vein thrombosis	Lobar	10.0 (6.1-16.1)	**0.0038**	1.77 (1.16-2.71)	**0.0083**
	Main	7.8 (3.9-14.3)		2.14 (1.24-3.71)	**0.0064**
	Patent	19.3 (15.3-20.8)			
	Segmental	15.2 (10.7-20.3)		1.13 (0.82-1.57)	0.4489
BCLC stage	A	41.4 (22.5-ND)	**<0.0001**		
	B	20.4 (14.9-24.9)		1.96 (1.17-3.28)	**0.011**
	C	12.6 (10.4-14.4)		3.35 (2.08-5.41)	**<0.0001**
	D	12.5 (4.0-.)		2.69 (0.79-9.10)	**0.1126**
Total bilirubin (mg/dl)	≤1.5	17.2 (14.4-20.0)	**0.0086**		
	>1.5	10.0 (4.7-14.4)		1.69 (1.14-2.51)	**0.0094**
Prior locoregional procedures	No	15.3 (12.6-19.2)	0.2126		
	Yes	17.9 (13.4-20.4)		0.85 (0.66-1.10)	0.2134
Prior surgery	No	15.3 (12.9-18.3)	**0.0248**		
	Yes	23.0 (13.4-36.8)		0.67 (0.47-0.95)	**0.0258**
Prior ablation	No	15.2 (12.9-18.7)	**0.0379**		
	Yes	22.4 (16.5-ND)		0.65 (0.43-0.98)	**0.0394**
Prior TACE	No	16.5 (14.0-19.6)	0.9520		
	Yes	16.0 (11.2-20.8)		1.01 (0.75-1.36)	0.9509
Prior abdominal radiotherapy	No	16.1 (14.0-19.2)	0.4204		
	Yes	29.3 (3.2-ND)		0.67 (0.25-1.80)	0.4229
Other prior embolotherapies	No	16.5 (14.0-19.3)	0.7285		
	Yes	14.4 (3.9-ND)		1.13 (0.58-2.19)	0.7288
Prior chemotherapy	No	17.0 (14.3-20.0)	0.0592		
	Yes	10.4 (7.1-19.2)		1.48 (0.98-2.22)	0.0609
Treatment intention	Curative*	22.9 (18.6-30.2)	**<0.0001**	0.53 (0.40-0.69)	**<0.0001**
	Palliative	12.2 (10.4-14.9)			
Dose methodology	BSA/mBSA	13.4 (11.5-16.1)	**<0.0001**		
	Partition model	23.4 (18.3-38.9)		0.53 (0.41-0.70	**<0.0001**
ALBI grade	1	21.1 (19.2-28.8)	**<0.0001**		
	2	14.0 (11.5-16.5)		1.66 (1.25-2.22)	**0.0005**
	3	7.8 (2.7-12.9)		3.92 (2.11-7.26)	**<0.0001**

Levels of significance: *p* <0.05 (Log-rank test [Mantel-Haenszel version]). Values in bold denote statistical significance.
ALBI, albumin-bilirubin; BCLC, Barcelona Clinic Liver Cancer; BSA, body surface area; ECOG, Eastern Cooperative Oncology Group; HR, hazard ratio; NASH, non-alcoholic steatohepatitis; TACE, transcatheter arterial chemoembolization; TARE, transarterial radioembolization.
* Curative reflects treatments for which there is a potential pathway to cure, *e.g.* bridging or downsizing for surgery or transplantation.

p = 0.0043; and grade 3: HR 2.80; 95% CI 1.49-5.28; *p* = 0.0014), curative treatment intention (HR 0.66; 95% CI 0.49-0.89; *p* = 0.0071) and partition model (HR 0.65; 95% CI 0.46-0.92; *p* = 0.0144, Table 4). Variables predicting PFS outcomes were BCLC B and C (BCLC B: HR 1.58; 95% CI 1.06-2.34; *p* = 0.025; BCLC C: HR 1.99; 95% CI 1.38-2.86; *p* = 0.0002), presence of cirrhosis (HR 1.31; 95% CI 1.03-1.67; *p* = 0.0312), curative treatment intention (HR 0.55; 95% CI 0.43-0.70; *p* <0.0001) and right-sided liver tumour (HR 0.73; 95% CI 0.57-0.92; *p* = 0.0092) (Table S4). For hPFS, these variables were ECOG 1 (HR 1.32; 95% CI 1.03-1.70; *p* = 0.0274), presence of cirrhosis (HR 1.43; 95% CI 1.11-1.85; *p* = 0.0060), right-sided tumours (HR 0.65; 95% CI 0.51-0.83; *p* = 0.0005), curative treatment intention (HR 0.59; 95% CI 0.46-0.75; *p* <0.0001) and lobar PVT (HR 1.71; 95% CI 1.15-2.56; *p* = 0.0086) (Table S5).

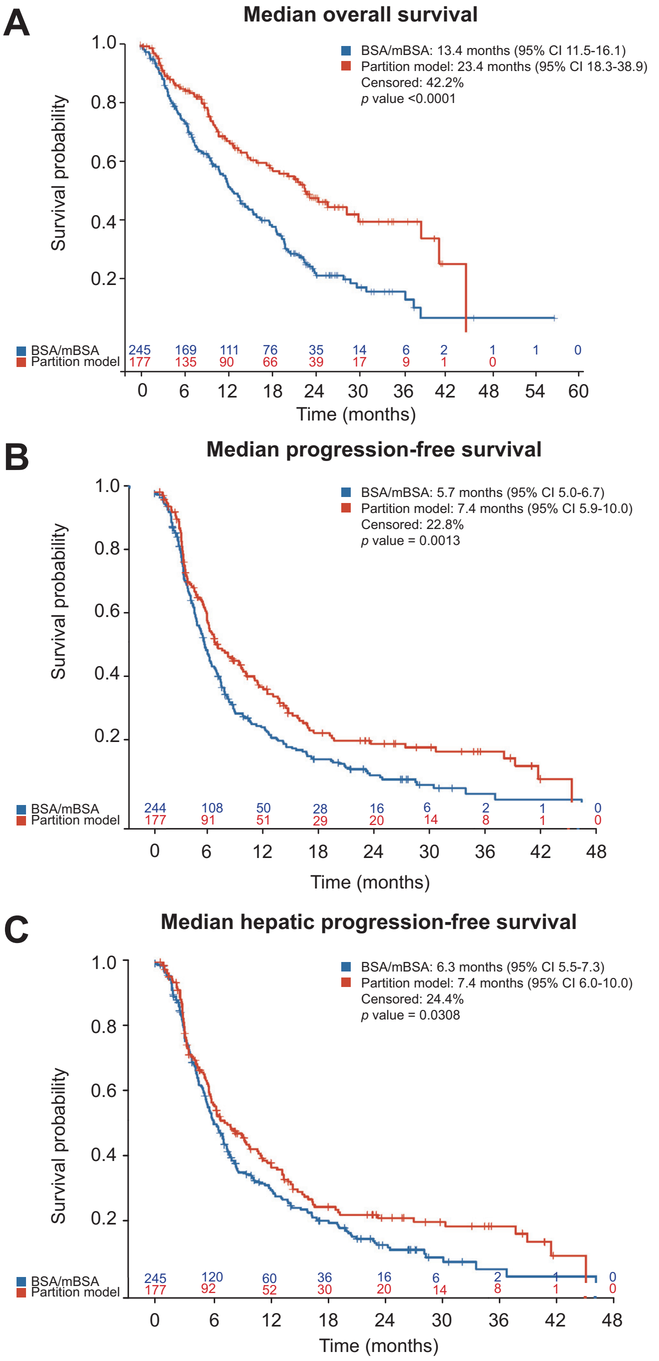

Fig. 1. Kaplan–Meier curves comparing outcomes between partition model dosimetry and (modified) body surface area dose calculation. Comparison of (A) overall survival, (B) progression-free survival and (C) hepatic progression-free survival (log-rank test [Mantel-Haenszel version], unadjusted).

Comparing outcomes between partition model dosimetry and BSA/mBSA

To further evaluate the differences in survival outcomes between partition model dosimetry and BSA/mBSA in the univariable analysis and the multivariable analysis for OS, we used propensity score matching to evaluate differences between the patient groups for whom the determination of the prescribed Y90 activity was performed either by BSA/mBSA or the partition model. The covariates considered in the model were based on the outcomes of the multivariable analysis: cirrhosis, ascites, number of tumour nodules, bilobar, right or left-sided tumours, PVT, and ALBI grade (Table S6, see Table S7 for a comparison of all baseline values in the two groups). In the exact matching model, 142 patients were matched (71 pairs were used), and 231 patients were excluded. The standardised mean differences are equal to zero, indicating a high degree of balance of patient characteristics across treatment groups for the application of matching. Compared to BSA/mBSA, the patients treated with the partition model experienced better outcomes in terms of OS (HR 0.56; 95% CI 0.35-0.89; p = 0.0136), but not PFS and hPFS (HR 0.69; 95% CI 0.47-1.01; p = 0.059 and HR 0.87; 95% CI 0.60-1.27, p = 0.4744, respectively) (Fig. 2). For the IPTW, 373 patients were included in the analysis. The smallest standardised difference magnitudes were observed for bilobar tumour location (d = −0.083) and cirrhosis (d = 0.088). All variables had standardised difference magnitudes of less than 0.09, indicating a good degree of balance of patient characteristics across treatment groups for the application of IPTW. The partition model was associated with better outcomes for OS (HR 0.52; 95% CI 0.39-0.69; p <0.0001), for PFS (HR 0.66; 95% CI 0.52-0.84; p = 0.0006) and for hPFS (HR 0.76; 95% CI 0.60-0.97; p = 0.0254), indicating that patients treated based on activity calculations with the partition model had better survival outcomes than patients treated based on BSA/mBSA activity calculations.

Furthermore, we did not find any difference in survival outcomes between patients in the BSA/mBSA cohort that were treated in hospitals that also contributed to the partition model cohort, and patients treated in hospitals that only used BSA/mBSA to calculate the prescribed activity (OS: HR 0.85; 95% CI 0.61-1.18; p = 0.3326; PFS: HR 0.94; 95% CI 0.69-1.27; p = 0.6684; hPFS: HR 0.98; 95% CI 0.72-1.33; p = 0.9) (Table S8).

Finally, comparing the prescribed activity between the partition model and BSA/mBSA revealed no significant differences when adjusted for tumour burden (Fig. S2) and the number of tumour nodules (Fig. S3).

Safety

A total of 115/422 (36.7%) patients experienced one or more adverse events. Gastrointestinal ulcerations (3/422, 0.7%), gastritis (3/422, 0.7%) and REILD (6/422, 1.4%) were uncommon. Severe adverse events (grade 3-5) were abdominal pain 2.1% (9/422), fatigue 1.4% (6/422), fever 0.5% (2/422), nausea 0.7% (3/422), vomiting 0.5% (2/422), gastrointestinal ulceration 0.2% (1/422), and REILD 0.2% (1/422) (Table S9).

Discussion

The HCC cohort collected in the CIRT study is one of the largest prospectively collected cohorts on the use of TARE in Europe. Despite the heterogeneous patient population, the multivariable analysis found that, compared with BSA, the partition model was a predictor of improved OS, but not PFS and hPFS. Additional propensity score matching, using the exact matching model and the IPTW model, found that patients whose prescribed activity was calculated with the partition model had better OS and PFS when compared with patients with similar baseline characteristics, but whose activity was prescribed based on BSA. Additionally, the IPTW model found an improved hPFS following the partition model. Other factors influencing OS outcomes were

Table 4. Multivariable analysis for overall survival.

Variable	Threshold	HR (95% CI)	*p* value
ECOG (*vs.* 0)	1	1.64 (1.20-2.23)	**0.0018**
	2+3	1.86 (1.09-3.16)	**0.0224**
Cirrhosis (*vs.* no)	Yes	1.39 (1.00-1.94)	**0.0480**
Ascites (*vs.* no)	Yes	1.62 (1.10-2.38)	**0.0152**
Location of tumour (*vs.* bilobar)	Left	0.64 (0.40-1.03)	0.0654
	Right	0.56 (0.41-0.76)	**0.0002**
Extrahepatic disease prior to treatment (*vs.* no)	Yes	1.55 (1.01-2.37)	**0.0455**
Portal vein thrombosis (*vs.* patent)	Lobar	1.40 (0.84-2.35)	0.2018
	Main	2.49 (1.37-4.54)	**0.0028**
	Segmental	1.46 (1.02-2.10)	**0.0378**
Treatment intention (*vs.* palliative)	Curative	0.66 (0.49-0.89)	**0.0071**
Dose methodology (*vs.* BSA/mBSA)	Partition model	0.65 (0.46-0.92)	**0.0144**
ALBI grade (*vs.* 1)	2	1.56 (1.15-2.12)	**0.0043**
	3	2.80 (1.49-5.28)	**0.0014**

Levels of significance: p <0.05 (Cox proportional-hazards model). Values in bold denote statistical significance. The proportional hazard function of the Cox model was verified. The following variables were considered in the multivariable model: Barcelona Clinic Liver Cancer stage; ECOG status; cirrhosis; ascites; tumour burden (nodules); location of tumour; extrahepatic disease prior to transarterial radioembolization; portal vein thrombosis; total bilirubin (mg/dl); prior surgery; prior ablation; prior chemotherapy; treatment intention; dose methodology; ALBI grade.
ALBI, albumin-bilirubin; BSA, body surface area; ECOG, Eastern Cooperative Oncology Group; HR, hazard ratio.

	Matching (n = 142)[a]		IPTW (n = 373)[b]	
	HR (95% CI)	*p* value	HR (95% CI)	*p* value
Overall survival	0.56 (0.35-0.89)	0.0136	0.52 (0.39-0.69)	<0.0001
Progression free survival	0.69 (0.47-1.01)	0.059	0.66 (0.52-0.84)	0.0006
Hepatic progression free survival	0.87 (0.60-1.27)	0.4744	0.76 (0.60-0.97)	0.0254

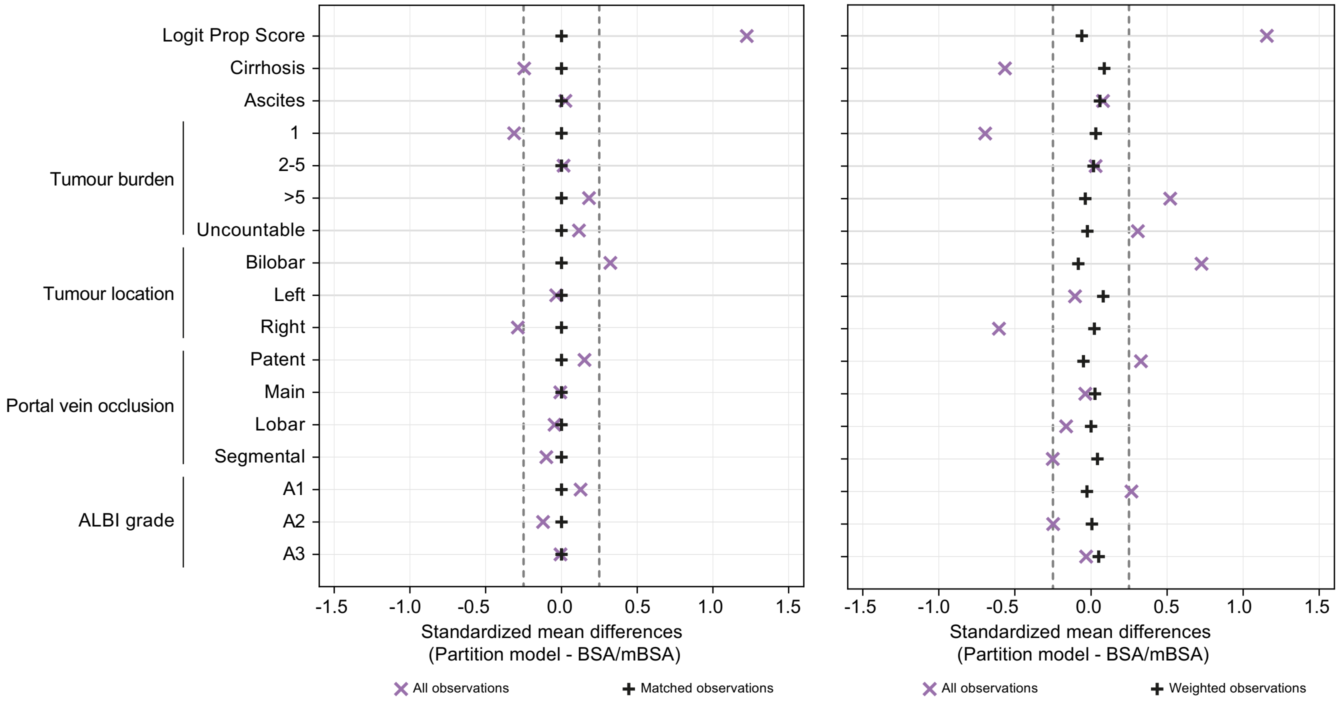

Fig. 2. Survival analysis of the propensity score matching. Levels of significance: p <0.05 (Cox proportional-hazards model). [a]Matching: Greedy nearest neighbour matching within a calliper of 0.2 of the propensity score. A total of 142 patients were matched (71 pairs were used), and 231 patients were excluded. The HR and 95% CI for the marginal treatment effect of OS, PFS and hPFS (Cox model with robust variance estimator that accounts for clustering within matched pairs) was presented as follow. The proportional hazard function of the Cox models was verified. The hazard ratio refers to the partition model. [b]Inverse probability treatment weighting: The HR and 95% CI for the relative treatment effect of OS, PFS and hPFS (Cox model adjusted for stabilised weights) was presented as follow. The proportional hazard function of the Cox models was verified. The hazard ratio refers to the partition model. hPFS, hepatic progression-free survival; HR, hazard ratio; IPTW, inverse probability of treatment weighting; OS, overall survival; PFS, progression-free survival.

ECOG >0, right-sided tumours, presence of ascites and PVT, and ALBI >1.

The median overall survival of our entire cohort of 422 patients was 16.5 months. This is in the range of 12.8-20.5 months reported by prior studies in similar real-life settings.[15,17,18,29] On the other hand, in the TARE arms of two prospective studies with randomisation against Sorafenib (SIRveNIB and SARAH), the median OS was only 8.0 and 8.8 months, respectively.[19,20] An important reason for the differences in survival is the selection of patients, *e.g.* while only one-third of our patients were classified as BCLC C, the randomised studies recruited a much higher percentage of patients in this advanced group (68% in the SARAH study and 48.4% in the SIRveNIB study in the intention-to-treat population). Finally, our study used Y90 resin microspheres instead of glass microspheres, but a comparison of both spheres in consecutive patients in a single-centre study (resin, n = 41; glass, n = 36) revealed no difference in survival between groups.[22]

In line with previous study outcomes, our multivariable analyses showed that ECOG >0, presence of ascites and extrahepatic disease were negative prognostic factors for OS.[30–32] Our data also confirms previous findings that the extent of PVT influences OS,[31,33–35] although in our analysis, lobar PVT was found to only influence hPFS, while segmental PVT and main PVT were found to impact OS. Our study further establishes that the ALBI grade was a strong independent predictor of OS, mirroring previous findings[27,36] and strengthening the justification for its inclusion in the recent BCLC strategy.[28]

Independent prognostic factors associated with OS identified in previous studies were albumin, alpha-fetoprotein, alkaline phosphatase and tumour size <5 cm.[22,29,31,32,37] The BCLC stage has also been identified as a prognostic factor for survival outcomes in several prior studies.[37–39] In our cohort, BCLC staging was a significant predictor of OS, PFS, and hPFS in the univariable analysis, but it was only a predictor for PFS in the multivariable analysis. A possible explanation is that BCLC is a composite variable consisting of variables that were found to be independent predictors, such as ECOG, PVT, and extrahepatic disease, and may thus not be independent from these variables. Additionally, a recent comparison of prognostic scoring systems in a cohort of patients receiving TARE ranked BCLC lower than other prognostic factors for this treatment.[28] The apparent difference in survival based on tumour location in the right *vs.* the left liver lobe found in our cohort may reflect the complexity and variation of blood supply to the liver, as recently described by Choi *et al.*[40] The variations in the blood supply of the left liver lobe may require a more meticulous positioning of suited microcatheters to ensure a consistent and robust dose distribution compared to the right liver lobe, explaining differences in outcome if not considered thoroughly.[41–43]

In terms of safety and toxicity, our cohort confirms previous reports on the favourable safety outcomes of TARE.[15,29,33,37,44] We observed a worsening of the liver function after TARE in terms of INR, bilirubin and albumin values (and therefore ALBI score), which mirrors the results of the SORAMIC randomised controlled trial, where in the TARE + sorafenib group, poorer ALBI scores after 4 and 6 months were observed compared to the sorafenib alone group.[45] Our study reported that 1.4% of patients experienced REILD, which was grade 3 or higher in half of affected patients. This occurrence of REILD is on the lower end of the studies used in the systematic review by Braat *et al.*, who identified that the incidence of symptomatic REILD varied between 0 and 31%, although, in most reports, the incidence was 0–8%.[46]

In our cohort, the multivariable analysis and the propensity score analyses showed that the partition model activity calculation led to better OS outcomes compared to BSA and mBSA, and improved PFS and hPFS in the propensity score analysis. The BSA and mBSA methods rely on an assumed correlation between BSA and the tumour burden to estimate Y90 activity. Ignoring the variability of the tumour-to-normal-liver ratio in individual patients, it sacrifices accuracy for simplicity,[47] and may result in wide variations of radiation dose absorbed by both the tumour and the surrounding non-tumoural liver parenchyma.[48,49] On the other hand, personalised dosimetry such as partition model relies on differential tumour-to-non-tumour perfusion evaluated on pretreatment Technetium-99m-macroaggregated albumin single-photon emission computer tomography combined with computer tomography to predict dose distributions between the "partitions" tumoural liver, non-tumoural liver, and lung. It has been demonstrated that personalised dosimetry models can increase the tumour-absorbed dose while keeping the dose in the non-tumoural liver and the lung low.[22] However, compared to the BSA model, these models are very resource-intensive and require a good collaboration between nuclear medicine physicians and interventional radiologists. Nevertheless, the data presented in this study strongly suggests that partition model dosimetry improves OS, PFS and hPFS outcomes in patients with unresectable HCC. These outcomes reflect recent studies examining the relationship between tumour-absorbed dose and survival outcomes in patients with HCC.

The randomised phase II DOSISPHERE-01 trial comparing patients with unresectable locally advanced HCC receiving personalised dosimetry with standard dosimetry showed that objective response was achieved in 20/28 patients (71%; 95% CI 51-87) in the personalised dosimetry group *vs.* 10/28 (36%; 95% CI 19-56) in the standard dosimetry group (p = 0.0074). This translated into a median OS of 26.6 months (95% CI 11.7-NR) in the personalised dosimetry group compared to 10.7 months (95% CI 6.0-16.8) in the standard dosimetry group. Furthermore, patients who received a tumour dose of 205 Gy or higher had an OS of 26.6 months (95% CI 13.5-NR) compared to 7.1 months (95% CI 4.6-14.8) in those that received a tumour dose of less than 205 Gy (HR 0.33; 95% CI 0.15-0.71; p = 0.0029).[23] Of note is that to achieve a high tumour-absorbed dose without increasing the dose absorbed by the non-tumoural liver, the DOSISPHERE-01 trial included only patients with tumours showing arterial phase hyperenhancement. Additionally, a secondary analysis of 120 patients from the SARAH study showed that participants who received at least 100 Gy (n = 67) had longer OS than those who received less than 100 Gy (median, 14.1 months [95% CI 9.6-18.6] *vs.* 6.1 months [95% CI 4.9-6.8], respectively; p = 0.001). In the patient group that was available for response analysis (n = 109), tumour radiation-absorbed dose was higher in patients with disease control *vs.* those with progressive disease (median, 121 Gy [IQR 86–190 Gy] *vs.* 85 Gy [IQR 58–164 Gy]; p = 0.02).[50] Unfortunately, the present study did not collect any data on the tumour target dose or radiation-absorbed dose and thus cannot provide any suggestions on optimal dosage. Analysis of differences in prescribed activity between BSA/mBSA and the partition model found no significant differences, when adjusted for tumour volume, percentage of tumour activity and number of tumour nodules. Nevertheless, the multivariable analysis and the propensity score matching suggest that patients whose dosages were calculated with the partition model generally performed better than patients whose dosage

was calculated with BSA or mBSA, which is in line with the aforementioned studies. Our study adds to the findings from DOSISPHERE-01 and SARAH, by showing that personalised dosimetry methods also improve effectiveness outcomes in a real-life clinical context with Y90 resin microspheres, compared to activity calculation methods based on BSA, irrespective of a site's experience.

A limitation of the study is the observational study design, whereby important confounding factors may not have been accounted for. The heterogeneity of the patient population reflects the real-life clinical practice in participating centres and thus its diversity in patient selection and clinical outcomes. We used the propensity score method and multivariable analysis to alleviate, to some degree, the effect of this heterogeneity and multiple methods of analysis were used to show the similarity of outcomes despite the differences in analysis. Of course, certain confounding factors which were not considered in these methods could have contributed to the outcomes and should be considered when interpreting the results.

The study was designed to explore the clinical outcomes of TARE and therefore focused less on dosimetry-specific data. This means that retrospectively important data points such as precise administered activity and tumour-absorbed dose were not included in the evaluation at the time of study design. Furthermore, as an observational study, the design was non-prescriptive for tumour response assessment, which was performed with various criteria (*e.g.*, RECIST, modified RECIST or PET Response Criteria in Solid Tumours) according to local practice and expertise of centres. This prevented us from including tumour response in the analysis.

We attempted to collect quality-of-life data from patients on a voluntary basis at the time of treatment and at every follow-up visit until study exit. The relevance of the collected dataset is currently being evaluated. The relatively high number of patients lost to follow-up can introduce bias regarding the interpretation of OS, and imprecise follow-up imaging intervals should be considered when interpreting PFS and hPFS. A potential explanation might be that TARE requires a comprehensive infrastructure with patients being referred to specialised centres for the treatment while being followed up by their local physician. In those cases, sites were encouraged to obtain follow-up information by contacting the referring physician. If this was not possible, the patient was considered as lost to follow-up. Selection bias can be expected in an observational study and regular remote monitoring was performed to verify that all eligible patients were included. Remote monitoring was done to improve data quality; however, no source data verification was performed.

This large prospective observational data set suggests that TARE with resin Y90 microspheres has a favourable toxicity profile and that patients with good liver function and no extra-hepatic disease are ideal candidates for this therapy. Furthermore, our data revealed that optimising the application of the therapy by using the partition model instead of BSA models, can significantly improve survival outcomes. It is thus recommended that activity calculations with the partition model are considered when designing future randomised controlled trials on TARE.

Abbreviations

ALBI, albumin-bilirubin; BCLC, Barcelona Clinic Liver Cancer; BSA, body surface area; CIRSE, Cardiovascular and Interventional Radiological Society of Europe; CIRT, CIRSE Registry for SIR-Spheres Therapy; ECOG, Eastern Cooperative Oncology Group; HCC, hepatocellular carcinoma; hPFS, hepatic progression-free survival; HR, hazard ratio; INR, international normalized ratio; IPTW, inverse probability of treatment weighting; mBSA, modified body surface area; OS, overall survival; PFS, progression-free survival; PVT, portal vein thrombosis; REILD, radioembolization-induced liver disease; TACE, transcatheter arterial chemoembolization; TARE, transarterial radioembolization; Y90, Yttrium-90.

Financial support

The CIRT study was funded by an independent investigator-initiated research grant from SIRTEX Medical Europe GmbH (Bonn, Germany). CIRSE, the Cardiovascular and Interventional Radiological Society of Europe, is responsible for the independent execution of the CIRT study and has sole ownership of the data.

Conflict of interest

Frank Kolligs participated on a data safety monitoring or advisory board of Bayer, MSD, and Roche. Dirk Arnold received consulting fees and honoraria for presentations and lectures and travel support from Boston Scientific and Terumo, is on the guidelines committee of the European Society for Medical Oncology, and supported oncopolicy manuscripts for the European Cancer Organisation. Rita Golfieri participated on a Data Safety Monitoring Board or Advisory Board and received payment or honoraria for lectures, presentations, speakers' bureaus, manuscript writing or educational events from Roche, Guerbet and Sirtex. Maciej Pech received grants or contracts and honoraria from lectures from Sirtex and Bayer. Maxime Ronot received honoraria for lectures from GE Healthcare, Ipsen, Canon-Toshiba, Alexion Pharmaceuticals, Guerbet, and Sirtex. Bruno Sangro received grants or contracts from Sirtex and BMS, consulting fees from Adaptimmune, Astra Zeneca, Bayer, BMS, Boston Scientific, Eisai, Eli Lilly, Incyte, Ipsen, Roche, Sirtex Medical, Terumo; Payment or honoraria for lectures, presentations, speakers bureaus, manuscript writing or educational events from Astra Zeneca, Bayer, BMS, Eisai, Incyte, Ipsen, Roche, Sirtex Medical; Participation on a data safety monitoring board or advisory board from Adaptimmune, Astra Zeneca, Bayer, BMS, Boston Scientific, Eisai, Eli Lilly, Incyte, Ipsen, Roche, Sirtex Medical, Terumo, and has a leadership or fiduciary role in the International Liver Cancer Association. Geert Maleux received honoraria for speaker's bureau from Sirtex Medical and operated as proctor for Sirtex. Bora Peynircioglu, Thomas Pfammatter, Niklaus Schaefer, Graham Munneke, Helena Pereira, Bleranda Zeka, Niels de Jong and Thomas Helmberger had nothing to declare.

Please refer to the accompanying ICMJE disclosure forms for further details.

Authors' contributions

FK, DA, BP, BS, NS, GMa, GMu, TH and NdJ contributed to the study concept, set up, and design. DA, RG, MP, TP, MR, BS, GMa and TH acquired patient data. FK, DA, RG, MR, BS, HP, NS, BZ and NdJ analysed and interpreted the data. FK, MR, BS, NS, HP, BZ, NdJ and TH drafted the manuscript. TH supervised the study. FK supervised the manuscript drafting and data interpretation. All authors contributed to critical revisions and approved the final version of the manuscript.

Data availability statement

Data access is limited by ethical and regulatory considerations.

Acknowledgements

The authors want to thank the patients, the CIRT investigators and site staff involved in the study, particularly the local study nurses who contributed significantly to the quality of the collected data through feedback and comments during the data collection phase. The authors are indebted to Prof. José Ignacio Bilbao, who initiated the study and was

chairperson of the CIRT Steering Committee between 2015 and 2018 and is now enjoying his well-deserved retirement. CIRSE, the Cardiovascular and Interventional Radiological Society of Europe, is responsible for the independent execution of the CIRT study and has sole ownership of the data. The electronic data capturing system was developed and supported by ConexSys Inc (Lincoln, RI, United States). ITEA GmbH (Vienna, Austria) developed and maintained the necessary infrastructure and a customised data management system was designed by Joaquin Padilla Montani, Vienna, Austria. Finally, the authors thank the CIRSE Central Office and the CIRSE Clinical Research department staff for their support during the design and setup of the study and drafting of the manuscript.

Supplementary data

Supplementary data to this article can be found online at https://doi.org/10.1016/j.jhepr.2022.100633.

References

[1] Rumgay H, Ferlay J, de Martel C, Georges D, Ibrahim AS, Zheng R, et al. Global, regional and national burden of primary liver cancer by subtype. Eur J Cancer 2022;161:108–118.
[2] Sung H, Ferlay J, Siegel RL, Laversanne M, Soerjomataram I, Jemal A, et al. Global cancer statistics 2020: GLOBOCAN estimates of incidence and mortality worldwide for 36 cancers in 185 countries. CA Cancer J Clin 2021;71(3):209–249.
[3] Giannini EG, Farinati F, Ciccarese F, Pecorelli A, Rapaccini GL, Di Marco M, et al. Prognosis of untreated hepatocellular carcinoma. Hepatology 2015;61(1):184–190.
[4] Benson AB, D'Angelica MI, Abbott DE, Anaya DA, Anders R, Are C, et al. Hepatobiliary cancers, version 2.2021, NCCN clinical practice guidelines in oncology. J Natl Compr Canc Netw 2021;19(5):541–565.
[5] European Association For The Study Of The L, European Organisation For R, Treatment Of C. EASL-EORTC clinical practice guidelines: management of hepatocellular carcinoma. J Hepatol 2012;56(4):908–943.
[6] European Association for the Study of the Liver, Electronic address eee, European Association for the Study of the L. EASL clinical practice guidelines: management of hepatocellular carcinoma. J Hepatol 2018;69(1):182–236.
[7] Marrero JA, Kulik LM, Sirlin CB, Zhu AX, Finn RS, Abecassis MM, et al. Diagnosis, staging, and management of hepatocellular carcinoma: 2018 practice guidance by the American Association for the Study of Liver Diseases. Hepatology 2018;68(2):723–750.
[8] Vogel A, Cervantes A, Chau I, Daniele B, Llovet JM, Meyer T, et al. Hepatocellular carcinoma: ESMO Clinical Practice Guidelines for diagnosis, treatment and follow-up. Ann Oncol 2019;30(5):871–873.
[9] Vogel A, Martinelli E. Updated treatment recommendations for hepatocellular carcinoma (HCC) from the ESMO Clinical Practice Guidelines. Ann Oncol 2021;32(6):801–805.
[10] Cheng AL, Kang YK, Chen Z, Tsao CJ, Qin S, Kim JS, et al. Efficacy and safety of sorafenib in patients in the Asia-Pacific region with advanced hepatocellular carcinoma: a phase III randomised, double-blind, placebo-controlled trial. Lancet Oncol 2009;10(1):25–34.
[11] Finn RS, Qin S, Ikeda M, Galle PR, Ducreux M, Kim TY, et al. Atezolizumab plus bevacizumab in unresectable hepatocellular carcinoma. N Engl J Med 2020;382(20):1894–1905.
[12] Kudo M, Finn RS, Qin S, Han KH, Ikeda K, Piscaglia F, et al. Lenvatinib versus sorafenib in first-line treatment of patients with unresectable hepatocellular carcinoma: a randomised phase 3 non-inferiority trial. Lancet 2018;391(10126):1163–1173.
[13] Llovet JM, Ricci S, Mazzaferro V, Hilgard P, Gane E, Blanc JF, et al. Sorafenib in advanced hepatocellular carcinoma. N Engl J Med 2008;359(4):378–390.
[14] Aaronson NK, Ahmedzai S, Bergman B, Bullinger M, Cull A, Duez NJ, et al. The European Organization for Research and Treatment of Cancer QLQ-C30: a quality-of-life instrument for use in international clinical trials in oncology. J Natl Cancer Inst 1993;85(5):365–376.
[15] Hilgard P, Hamami M, Fouly AE, Scherag A, Muller S, Ertle J, et al. Radioembolization with yttrium-90 glass microspheres in hepatocellular carcinoma: European experience on safety and long-term survival. Hepatology 2010;52(5):1741–1749.
[16] Salem R, Gilbertsen M, Butt Z, Memon K, Vouche M, Hickey R, et al. Increased quality of life among hepatocellular carcinoma patients treated with radioembolization, compared with chemoembolization. Clin Gastroenterol Hepatol 2013;11(10):1358–13565 e1.
[17] Salem R, Lewandowski RJ, Mulcahy MF, Riaz A, Ryu RK, Ibrahim S, et al. Radioembolization for hepatocellular carcinoma using Yttrium-90 microspheres: a comprehensive report of long-term outcomes. Gastroenterology 2010;138(1):52–64.
[18] Sangro B, Carpanese L, Cianni R, Golfieri R, Gasparini D, Ezziddin S, et al. Survival after yttrium-90 resin microsphere radioembolization of hepatocellular carcinoma across Barcelona clinic liver cancer stages: a European evaluation. Hepatology 2011;54(3):868–878.
[19] Chow PKH, Gandhi M, Tan SB, Khin MW, Khasbazar A, Ong J, et al. SIRveNIB: selective internal radiation therapy versus sorafenib in Asia-Pacific patients with hepatocellular carcinoma. J Clin Oncol 2018;36(19):1913–1921.
[20] Vilgrain V, Pereira H, Assenat E, Guiu B, Ilonca AD, Pageaux GP, et al. Efficacy and safety of selective internal radiotherapy with yttrium-90 resin microspheres compared with sorafenib in locally advanced and inoperable hepatocellular carcinoma (SARAH): an open-label randomised controlled phase 3 trial. Lancet Oncol 2017;18(12):1624–1636.
[21] Sposito C, Mazzaferro V. The SIRveNIB and SARAH trials, radioembolization vs. sorafenib in advanced HCC patients: reasons for a failure, and perspectives for the future. Hepatobiliary Surg Nutr 2018;7(6):487–489.
[22] Van Der Gucht A, Jreige M, Denys A, Blanc-Durand P, Boubaker A, Pomoni A, et al. Resin versus glass microspheres for (90)Y transarterial radioembolization: comparing survival in unresectable hepatocellular carcinoma using pretreatment partition model dosimetry. J Nucl Med 2017;58(8):1334–1340.
[23] Garin E, Tselikas L, Guiu B, Chalaye J, Edeline J, de Baere T, et al. Personalised versus standard dosimetry approach of selective internal radiation therapy in patients with locally advanced hepatocellular carcinoma (DOSISPHERE-01): a randomised, multicentre, open-label phase 2 trial. Lancet Gastroenterol Hepatol 2021;6(1):17–29.
[24] Garin E, Tzelikas L, Guiu B, Chalaye J, Edeline J, Baere TD, et al. Major impact of personalized dosimetry using 90Y loaded glass microspheres SIRT in HCC: final overall survival analysis of a multicenter randomized phase II study (DOSISPHERE-01). J Clin Oncol 2020;38(4_suppl):516.
[25] Helmberger T, Golfieri R, Pech M, Pfammatter T, Arnold D, Cianni R, et al. Clinical application of trans-arterial radioembolization in hepatic malignancies in Europe: first results from the prospective multicentre observational study CIRSE Registry for SIR-spheres therapy (CIRT). Cardiovasc Intervent Radiol 2021;44(1):21–35.
[26] Helmberger T, Arnold D, Bilbao JI, de Jong N, Maleux G, Nordlund A, et al. Clinical application of radioembolization in hepatic malignancies: protocol for a prospective multicenter observational study. JMIR Res Protoc 2020;9(4):e16296.
[27] Johnson PJ, Berhane S, Kagebayashi C, Satomura S, Teng M, Reeves HL, et al. Assessment of liver function in patients with hepatocellular carcinoma: a new evidence-based approach-the ALBI grade. J Clin Oncol 2015;33(6):550–558.
[28] Reig M, Forner A, Rimola J, Ferrer-Fabrega J, Burrel M, Garcia-Criado A, et al. BCLC strategy for prognosis prediction and treatment recommendation Barcelona Clinic Liver Cancer (BCLC) staging system. The 2022 update. J Hepatol 2021;76(3):681–693.
[29] Op den Winkel M, Nagel D, de la Torre Alaez MA, Hempe S, De Toni EN, Paprottka KJ, et al. A simple prognostic scoring system for hepatocellular carcinoma treated with selective internal radiation therapy. Dig Dis 2022;40(3):322–334.
[30] Abdallah MA, Wongjarupong N, Hassan MA, Taha W, Abdalla A, Bampoh S, et al. The efficacy, safety, and predictors of outcomes of transarterial radioembolization for hepatocellular carcinoma: a retrospective study. Expert Rev Gastroenterol Hepatol 2020;14(7):619–629.
[31] Abouchaleh N, Gabr A, Ali R, Al Asadi A, Mora RA, Kallini JR, et al. (90)Y radioembolization for locally advanced hepatocellular carcinoma with portal vein thrombosis: long-term outcomes in a 185-patient cohort. J Nucl Med 2018;59(7):1042–1048.
[32] Tohme S, Bou Samra P, Kaltenmeier C, Chidi AP, Varley PR, Tsung A. Radioembolization for hepatocellular carcinoma: a nationwide 10-year experience. J Vasc Interv Radiol 2018;29(7):912–919 e2.
[33] Garin E, Rolland Y, Edeline J, Icard N, Lenoir L, Laffont S, et al. Personalized dosimetry with intensification using 90Y-loaded glass microsphere radioembolization induces prolonged overall survival in hepatocellular carcinoma patients with portal vein thrombosis. J Nucl Med 2015;56(3):339–346.
[34] Kulik LM, Carr BI, Mulcahy MF, Lewandowski RJ, Atassi B, Ryu RK, et al. Safety and efficacy of 90Y radiotherapy for hepatocellular carcinoma with and without portal vein thrombosis. Hepatology 2008;47(1):71–81.
[35] Spreafico C, Sposito C, Vaiani M, Cascella T, Bhoori S, Morosi C, et al. Development of a prognostic score to predict response to Yttrium-90

radioembolization for hepatocellular carcinoma with portal vein invasion. J Hepatol 2018;68(4):724–732.
[36] Peng Y, Wei Q, He Y, Xie Q, Liang Y, Zhang L, et al. ALBI versus child-pugh in predicting outcome of patients with HCC: a systematic review. Expert Rev Gastroenterol Hepatol 2020;14(5):383–400.
[37] Gao R, Gabr A, Mouli S, Riaz A, Kulik L, Lewandowski RJ, et al. Toxicity and survival of hepatocellular carcinoma patients with hepatitis B infection treated with yttrium-90 radioembolization: an updated 15-year study. J Vasc Interv Radiol 2020;31(3):401–408 e1.
[38] Salem R, Gabr A, Riaz A, Mora R, Ali R, Abecassis M, et al. Institutional decision to adopt Y90 as primary treatment for hepatocellular carcinoma informed by a 1,000-patient 15-year experience. Hepatology 2018;68(4):1429–1440.
[39] Sangro B, Maini CL, Ettorre GM, Cianni R, Golfieri R, Gasparini D, et al. Radioembolisation in patients with hepatocellular carcinoma that have previously received liver-directed therapies. Eur J Nucl Med Mol Imaging 2018;45(10):1721–1730.
[40] Choi TW, Chung JW, Kim HC, Lee M, Choi JW, Jae HJ, et al. Anatomic variations of the hepatic artery in 5625 patients. Radiol Cardiothorac Imaging 2021;3(4):e210007.
[41] Aramburu J, Anton R, Rivas A, Ramos JC, Sangro B, Bilbao JI. Numerical investigation of liver radioembolization via computational particle-hemodynamics: the role of the microcatheter distal direction and microsphere injection point and velocity. J Biomech 2016;49(15):3714–3721.
[42] Borg P, Wong JJ, Lawrance N, Chander A, Tipping J, Bell J, et al. Vascular redistribution for SIRT—a quantitative assessment of treatment success and long-term analysis of recurrence and survival outcomes. J Clin Interv Radiol ISVIR 2019;3(2):89–97.
[43] Spina JC, Hume I, Pelaez A, Peralta O, Quadrelli M, Garcia Monaco R. Expected and unexpected imaging findings after (90)Y transarterial radioembolization for liver tumors. Radiographics 2019;39(2):578–595.
[44] Ricke J, Schinner R, Seidensticker M, Gasbarrini A, Van Delden OM, Amthauer H, et al. Liver function after combined selective internal radiation therapy or sorafenib monotherapy in advanced hepatocellular carcinoma. J Hepatol 2021;75(6):1387–1396.
[45] Loffroy R, Ronot M, Greget M, Bouvier A, Mastier C, Sengel C, et al. Short-term safety and quality of life outcomes following radioembolization in primary and secondary liver tumours: a multi-centre analysis of 200 patients in France. Cardiovasc Intervent Radiol 2021;44(1):36–49.
[46] Braat MN, van Erpecum KJ, Zonnenberg BA, van den Bosch MA, Lam MG. Radioembolization-induced liver disease: a systematic review. Eur J Gastroenterol Hepatol 2017;29(2):144–152.
[47] Kao YH, Tan EH, Ng CE, Goh SW. Clinical implications of the body surface area method versus partition model dosimetry for yttrium-90 radioembolization using resin microspheres: a technical review. Ann Nucl Med 2011;25(7):455–461.
[48] Gil-Alzugaray B, Chopitea A, Inarrairaegui M, Bilbao JI, Rodriguez-Fraile M, Rodriguez J, et al. Prognostic factors and prevention of radioembolization-induced liver disease. Hepatology 2013;57(3):1078–1087.
[49] Sangro B, Gil-Alzugaray B, Rodriguez J, Sola I, Martinez-Cuesta A, Viudez A, et al. Liver disease induced by radioembolization of liver tumors: description and possible risk factors. Cancer 2008;112(7):1538–1546.
[50] Hermann AL, Dieudonne A, Ronot M, Sanchez M, Pereira H, Chatellier G, et al. Relationship of tumor radiation-absorbed dose to survival and response in hepatocellular carcinoma treated with transarterial radioembolization with (90)Y in the SARAH study. Radiology 2020;296(3):673–684.

Journal of Hepatology, Volume *5*

Supplemental information

Factors impacting survival after transarterial radioembolization in patients with hepatocellular carcinoma: Results from the prospective CIRT study

Frank Kolligs, Dirk Arnold, Rita Golfieri, Maciej Pech, Bora Peynircioglu, Thomas Pfammatter, Maxime Ronot, Bruno Sangro, Niklaus Schaefer, Geert Maleux, Graham Munneke, Helena Pereira, Bleranda Zeka, Niels de Jong, Thomas Helmberger, and on behalf of the CIRT Principal Investigators

Factors impacting survival after transarterial radioembolization in patients with hepatocellular carcinoma: Results from the prospective CIRT study

Frank Kolligs, Dirk Arnold, Rita Golfieri, Maciej Pech, Bora Peynircioglu, Thomas Pfammatter, Maxime Ronot, Bruno Sangro, Niklaus Schaefer, Geert Maleux, Graham Munneke, Helena Pereira, Bleranda Zeka, Niels de Jong, Thomas Helmberger, on behalf of the CIRT principal investigators

Table of contents

CIRT principal investigators..........2

Supplementary figures..........4

Supplementary tables..........7

CIRT principal investigators

Thomas Albrecht, Vivantes Klinikum Neukölln, Department for Radiology and Interventional Therapy, Rudower Str. 48, 12351, Berlin, Germany.

Olivier D'Archambeau, University Hospital Antwerp, Department of Radiology, Wilrijkstraat 10, 2650, Antwerp, Belgium.

Tugsan Balli, Cukurova University, Radiology Department, Balcalı Hospital, 01330, Adana, Turkey.

Sadik Bilgic, Ankara University, Department of Radiology, Medical Faculty, Cebeci, 06590, Ankara, Turkey.

Allan Bloom, Hadassah-Hebrew University Medical Center, Department of Radiology, Jerusalem, Israel.

Roberto Cioni, University of Pisa, Diagnostic and Interventional Radiology, Department of Translational Research and New Technologies in Medicine, Via U. Foscolo 5, 50059, Vinci, Pisa, Italy.

Roman Fischbach, Asklepios Klinik Altona, Department of Radiology and Neuroradiology, Paul-EhrlichStraße 1, 22763, Hamburg, Germany.

Patrick Flamen, Institute Jules Bordet, Université Libre de Bruxelles, Nuclear Medicine Department, 121 Boulevard de Waterloo, 1000, Brussels, Belgium.

Laurent Gerard, University Hospital of Liege, Division of Radiology, domaine du Sart-Tilman B35, 4000, Liège, Belgium.

Gerd Grözinger, Eberhard Karls University, Department of Diagnostic and Interventional Radiology, Hoppe-Seyler-Str. 3, D-72076, Tübingen, Germany.

Marcus Katoh, Helios Hospital Krefeld, Department of Diagnostic and Interventional Radiology, Lutherplatz 40, 47805, Krefeld, Germany.

Michael Koehler, University Hospital Muenster, Department of Clinical Radiology, Albert-Schweitzer-Strasse 33, 48129, Muenster, Germany.

Jan Robert Kröger, Johannes Wesling Klinik Minden, Universitätsinstitut für Radiologie, Neuroradiologie und Nuklearmedizin der Mühlenkreiskliniken, Hans-Nolte-Straße 1, 32429 Minden, Germany.

Christiane Kuhl, University Hospital Aachen, Department of Radiology, Pauwelsstr. 30, 52074, Aachen, Germany.

Franco Orsi, European Institute of Oncology, Interventional Radiology Division, Via Ripamonti 435, 20100, Milan, Italy.

Murat Özgün, St. Franziskus Hospital, Department of Radiology, Hohenzollernring 70, 48145, Muenster, Germany.

Peter Reimer, Academic Teaching Hospital the University of Freiburg, Städtisches Klinikum Karlsruhe, Institute for Diagnostic and Interventional Radiology, Moltkestrasse 90, 76133, Karlsruhe, Germany.

Maxime Ronot, APHP, University Hospitals Paris Nord Val de Seine, Department of Radiology, 100 bd général Leclerc, 100 bd general Leclerc, Beaujon, Clichy, Hauts-de-Seine, France.

Axel Schmid, University Hospital Erlangen, Department of Radiology, Maximiliansplatz 1, 91054, Erlangen, Germany.

Alessandro Vit, Azienda Ospedaliero Universitaria, SOC Diagnostica Angiografica e Radiologia Interventistica, via Grazzano 150/C, 33100, Udine, Italy.

Supplementary figures

Fig. S1. Mean (SD) evolution of ALBI, bilirubin, albumin and INR

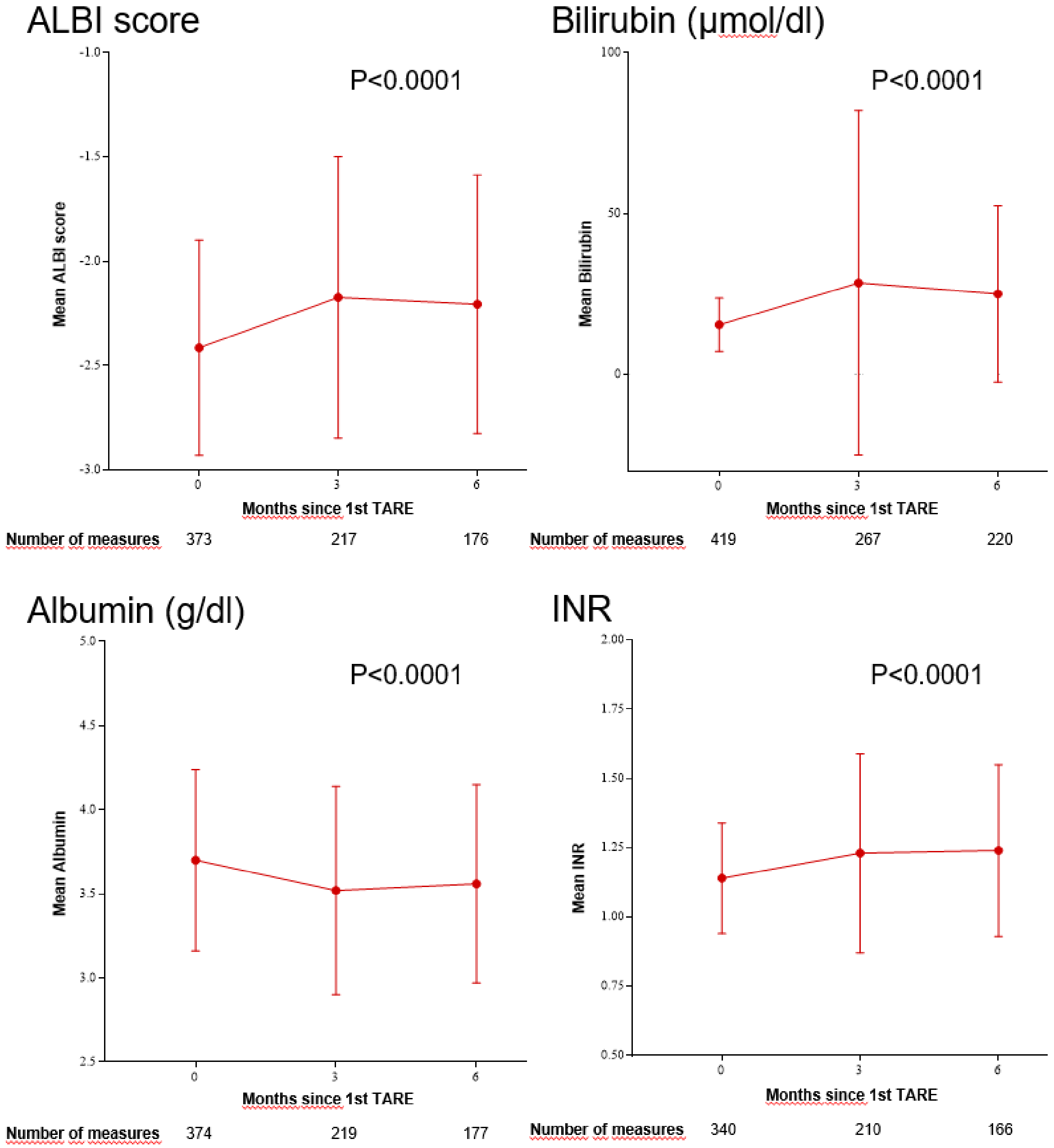

Levels of significance: $p < 0.05$ (Linear mixed model)
A linear mixed model was used to explore time effect for ALBI, bilirubin, albumin and INR with patient's effect as random variable (to take into account between-patient differences).
ALBI: Albumin-Bilirubin; INR: International Normalised Ratio; TARE: Transarterial Radioembolization.

Fig. S2. Comparing the prescribed activity between partition model and BSA/mBSA, adjusted for tumour burden.

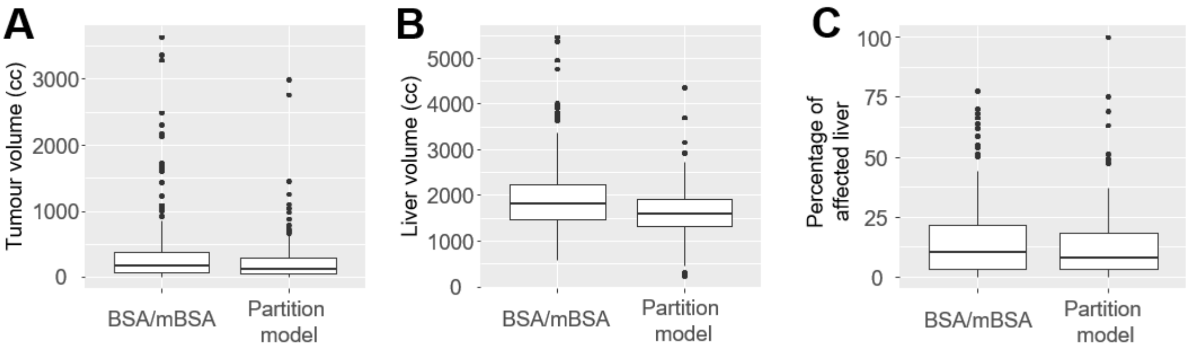

Median (IQR)	174 (59-377)	119 (51-294)
n (%)	219 (52)	174 (41)
Missing, n (%)	26 (6)	3 (<1)
P value	0.03	

Median (IQR)	1798 (1446-2224)	1587 (1309-1903)
n (%)	219 (52)	177 (42)
Missing, n (%)	26 (6)	0
P value	<0.001	

Median (IQR)	9.1 (3.4-20.4)	7.9 (3.3-18.3)
n (%)	216 (51)	174 (41)
Missing, n (%)	29 (7)	3 (<1)
P value	0.16	

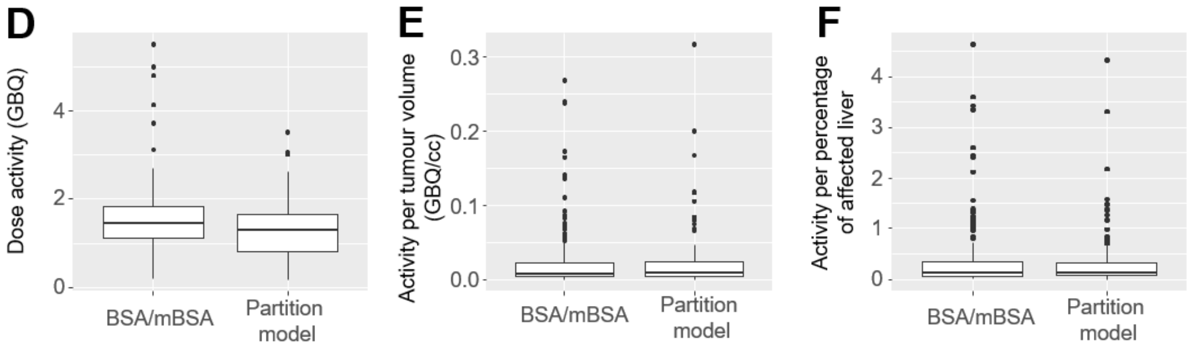

Median (IQR)	1.4 (1.0-1.8)	1.3 (0.8-1.7)
n (%)	245 (58)	177 (42)
Missing, n (%)	0	0
P value	0.003	

Median (IQR)	0.008 (0.004-0.02)	0.009 (0.005-0.024)
n (%)	216 (51)	174 (41)
Missing, n (%)	29 (7)	3 (<1)
P value	0.24	

Median (IQR)	0.13 (0.07-0.34)	0.13 (0.08-0.33)
n (%)	216 (51)	174 (41)
Missing, n (%)	29 (7)	3 (<1)
P value	0.81	

Levels of significance: $p < 0.05$ (Wilcoxon-Mann-Whitney-Test).

We compared the prescribed activity (GBq) between patients whose prescribed activity was calculated with BSA or mBSA and partition model. Patients treated with the partition model generally had smaller tumours (**A**, in cc) and livers (**B**) but the percentage of the liver affected by tumour (**C**) was similar as patients treated with BSA/mBSA. The prescribed activity using BSA/mBSA was significantly higher than using partition model (**D**, p=0.003), but adjusted for tumour volume (**E**) or percentage of affected liver (**F**), no significant difference in prescribed dose was found.

Fig. S3. Comparing the prescribed activity between the partition model and BSA/mBSA, adjusted for number of tumour nodules.

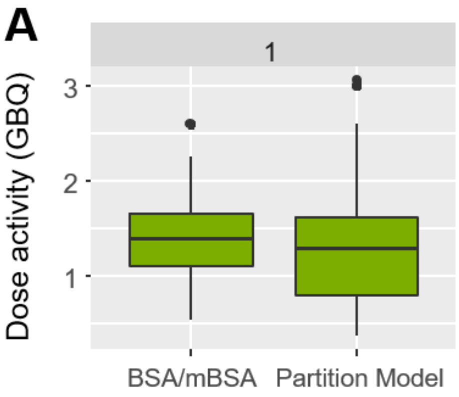

Median (IQR)	1.4 (1.1-1.6)	1.3 (0.8-1.6)
n (%)	47 (11)	89 (21)
Missing, n (%)	0	0
P value	0.22	

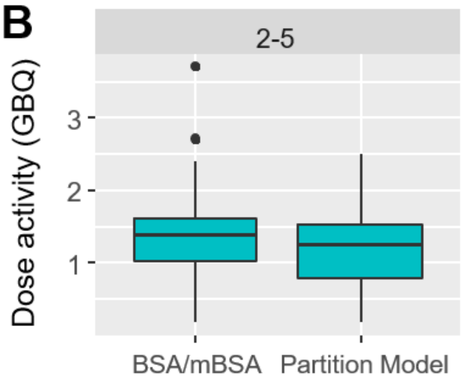

Median (IQR)	1.4 (1.0-1.6)	1.3 (0.8-1.5)
n (%)	81 (19)	57 (14)
Missing, n (%)	0	0
P value	0.12	

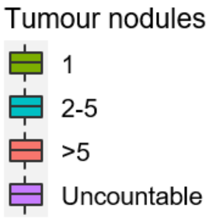

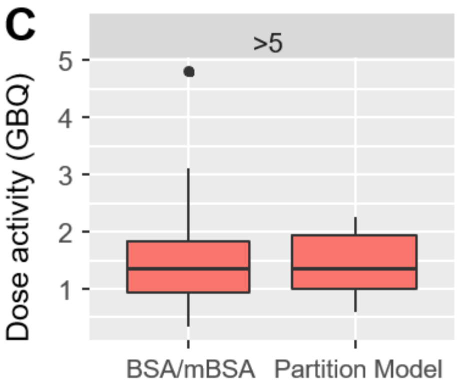

Median (IQR)	1.4 (0.9-1.8)	1.4 (1.0-1.9)
n (%)	61 (15)	11 (3)
Missing, n (%)	0	0
P value	0.75	

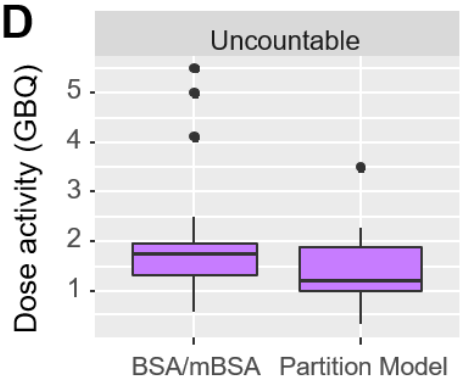

Median (IQR)	1.8 (1.3-2.0)	1.2 (1.0-1.9)
n (%)	56 (13)	20 (5)
Missing, n (%)	0	0
P value	0.06	

Levels of significance: $p < 0.05$ (Wilcoxon-Mann-Whitney-Test).
We compared the prescribed activity (GBq) between patients whose prescribed activity was calculated with BSA or mBSA and partition model. Adjusting for number of tumour nodules, we found no significant differences between the prescribed activity using BSA/mBSA or the partition model for number of nodules 1 (**A**), 2-5 (**B**), >5 (**C**), or uncountable (**D**).

Supplementary tables

Table S1. Post-TARE chemotherapies

Category	Subcategory	HCC (n=422)
Post-TARE systemic chemotherapy	Missing	35 (8.3%)
	No	262 (62.1%)
	Yes	125 (29.6%)
Post-TARE systemic chemotherapy: number of lines	Missing	298 (70.6%)
	One line	38 (9.0%)
	Two lines	19 (4.5%)
	Three lines	21 (5.0%)
	Four lines	9 (2.1%)
	Five lines	10 (2.4%)
	Six lines	6 (1.4%)
	More than six lines	21 (5.0%)
Single systemic therapy	Yes	99 (23.5%)
Therapy	SORAFENIB	53 (53.5%)
	REGORAFENIB	21 (21.2%)
	CAPECITABINE	8 (8.1%)
	5-FLUOROURACIL	
	NIVOLUMAB	7 (7.1%)
	GEMCITABINE	4 (4.0%)
	CABOZANTINIB	3 (3.0%)
	LENVATINIB	3 (3.0%)
Multiple systemic therapies	Yes	3 (0.7%)
Number of systemic regimens [a]	1 regimen	2 (66.7%)
	2 regimens	
	3-5 regimens	1 (33.3%)
	6-10 regimens	3 (0.7%)
Type of therapy	GEMOX	3 (100.0%)
	Not categorized	3 (100.0%)

[a] regimen is understood as a combination of several chemotherapeutic agents
GEMOX: Gemictabin and oxaliplatin; TARE: Transarterial radioembolization

Table S2. Univariable analysis for progression-free survival

**Variable**	**Threshold**	**Median (95% CI)**	**p value**	**HR (95% CI)**	**p value HR**
Age (years)	<68	5.6 (4.7-7.0)	0.4267		
	>=68	6.7 (5.9-7.9)		0.92 (0.74-1.14)	0.4264
Gender	Female	7.0 (4.7-10.3)	0.3161	0.86 (0.65-1.15)	0.3176
	Male	6.0 (5.5-6.8)			
ECOG	0	6.9 (6.0-7.9)	**0.0004**		
	1	5.4 (4.4-6.0)		1.55 (1.23-1.95)	**0.0002**
	2+3	5.7 (3.5-8.5)		1.57 (1.02-2.41)	**0.0388**
Cirrhosis	No	7.0 (5.6-9.5)	0.1285		
	Yes	6.0 (5.5-6.7)		1.21 (0.95-1.54)	0.1293
Cause of cirrhosis	Alcohol	5.7 (4.5-7.2)	**0.0383**		
	Hepatitis-B	4.1 (3.4-6.5)		0.94 (0.64-1.40)	0.7695
	Hepatitis-C	7.7 (5.7-12.2)		0.62 (0.44-0.87)	**0.0051**
	NASH	5.5 (3.5-7.6)		0.97 (0.64-1.48)	0.8903
	Other	7.1 (4.3-9.7)		0.88 (0.58-1.33)	0.5335
Ascites	No	6.3 (5.7-7.3)	0.1915		
	Yes	5.5 (3.5-6.7)		1.23 (0.90-1.67)	0.1907
Number of nodules	1	7.7 (6.3-9.5)	**0.0019**		
	2-5	6.2 (5.6-7.7)		1.25 (0.95-1.63)	0.1070
	>5	5.3 (4.0-7.3)		1.56 (1.13-2.16)	**0.0066**
	Uncountable	4.7 (3.7-5.8)		1.76 (1.28-2.42)	**0.0005**
Location of tumour	Bilobar	5.3 (4.4-6.1)	**0.0031**		
	Left	6.6 (3.6-8.6)		0.78 (0.55-1.11)	0.1704
	Right	7.0 (5.9-8.6)		0.67 (0.53-0.85)	**0.0007**
Extra-hepatic disease prior to treatment	No	6.3 (5.7-7.2)	0.1314		
	Yes	4.7 (3.3-7.2)		1.33 (0.92-1.92)	0.1318
Portal vein thrombosis	Lobar	5.5 (3.5-6.5)	0.3132	1.34 (0.91-1.95)	0.1346
	Main	4.7 (3.0-8.0)		1.30 (0.79-2.13)	0.2989
	Patent	6.7 (5.7-7.9)			
	Segmental	5.8 (5.0-7.2)		1.16 (0.88-1.52)	0.2957
BCLC stage	A	12.5 (7.7-17.2)	**0.0002**		
	B	6.0 (5.3-7.6)		1.74 (1.17-2.57)	**0.0058**
	C	5.6 (4.7-6.1)		2.17 (1.51-3.11)	**<0.0001**
	D	11.2 (4.0-12.7)		1.37 (0.48-3.86)	0.5558
Total bilirubin (mg/dl)	≤1.5	6.2 (5.7-7.2)	0.3282		
	>1.5	4.6 (3.3-8.4)		1.20 (0.83-1.74)	0.3293
Prior procedures	No	6.3 (5.5-7.2)	0.8554		
	Yes	6.0 (4.9-7.6)		0.98 (0.79-1.22)	0.8575
Surgery	No	6.3 (5.7-7.0)	0.7168		
	Yes	5.8 (4.3-7.6)		0.95 (0.72-1.26)	0.7189
Ablation	No	5.8 (5.5-6.6)	**0.0468**		
	Yes	9.2 (6.0-14.0)		0.72 (0.52-1.00)	**0.0478**
TACE	No	6.3 (5.7-7.2)	0.2557		
	Yes	5.8 (3.7-8.6)		1.16 (0.90-1.49)	0.2555
Abdominal radiotherapy	No	6.0 (5.6-7.0)	0.2298		
	Yes	11.6 (3.0-ND)		0.59 (0.24-1.42)	0.2351

Other prior embolotherapies	No	6.3 (5.7-7.2)	0.3602		
	Yes	4.0 (3.0-5.9)		1.30 (0.75-2.26)	0.3575
Prior chemotherapy	No	6.3 (5.7-7.3)	0.1889		
	Yes	5.2 (3.5-6.5)		1.27 (0.89-1.81)	0.1897
Treatment intention	Ablation	13.6 (5.8-30.4)	**<0.0001**	0.43 (0.23-0.79)	**0.0068**
	Bridge to surgery or transplant	7.6 (4.4-ND)		0.44 (0.26-0.74)	**0.0021**
	Down-sizing/down-staging	9.5 (6.7-13.6)		0.55 (0.43-0.70)	**<0.0001**
	Palliative	5.3 (4.5-5.8)			
Treatment intention	Curative	9.5 (7.2-13.6)	**<0.0001**	0.52 (0.41-0.65)	**<0.0001**
	Palliative	5.3 (4.5-5.8)			
Dose methodology	BSA/mBSA	5.7 (5.0-6.7)	**0.0013**		
	Partition model	7.4 (5.9-10.0)		0.70 (0.56-0.87)	**0.0014**
ALBI grade	1	6.8 (5.7-8.1)	**0.0209**		
	2	5.9 (5.3-6.7)		1.09 (0.86-1.38)	0.4665
	3	4.6 (2.6-6.6)		2.18 (1.24-3.80)	**0.0064**

Levels of significance: $p < 0.05$ (Log-rank test [Mantel-Haenszel version]).
ALBI: Albumin Bilirubin; BCLC Barcelona Clinic Liver Cancer; BSA: Body Surface Area; ECOG: Eastern Cooperative Oncology Group; NASH: Non-alcoholic steatohepatitis; TACE: Transcatheter Arterial Chemoembolization; TARE: Transarterial Radioembolization.

Table S3. Univariable analysis for hepatic progression free survival

**Variable**	**Threshold**	**Median (95% CI)**	**p value**	**HR (95% CI)**	**p value HR**
Age (years)	<68	6.0 (5.3-7.6)	0.3446		
	>=68	7.2 (6.2-8.5)		0.90 (0.73-1.12)	0.3445
Gender	Female	7.7 (5.4-12.3)	0.3924	0.88 (0.66-1.18)	0.3935
	Male	6.6 (5.8-7.4)			
ECOG	0	7.3 (6.3-8.7)	**0.0016**		
	1	5.7 (5.2-7.4)		1.45 (1.14-1.84)	**0.0021**
	2+3	5.7 (3.5-8.5)		1.70 (1.11-2.61)	**0.0152**
Cirrhosis	No	7.9 (6.3-12.3)	**0.0292**		
	Yes	6.2 (5.7-7.2)		1.32 (1.03-1.69)	**0.0297**
Cause of cirrhosis	Alcohol	5.9 (4.6-7.7)	0.0854		
	Hepatitis-B	4.1 (3.4-6.6)		0.99 (0.67-1.47)	0.9642
	Hepatitis-C	7.7 (5.7-12.2)		0.64 (0.46-0.90)	**0.0111**
	NASH	5.7 (4.3-8.7)		0.89 (0.58-1.35)	0.5722
	Other	8.2 (5.2-12.0)		0.83 (0.54-1.26)	0.3811
Ascites	No	7.0 (6.0-8.0)	0.0911		
	Yes	5.5 (3.7-7.2)		1.31 (0.96-1.78)	0.0907
Number of nodules	1	8.0 (6.6-9.7)	**0.0028**		
	2-5	6.8 (5.8-9.5)		1.17 (0.89-1.54)	0.2588
	>5	6.1 (4.2-8.7)		1.47 (1.06-2.03)	**0.0218**
	Uncountable	4.7 (3.7-5.8)		1.76 (1.28-2.42)	**0.0005**
Location of tumour	Bilobar	5.6 (4.7-6.7)	**0.0021**		
	Left	6.7 (4.0-9.2)		0.81 (0.57-1.16)	0.2596
	Right	8.0 (6.5-10.3)		0.66 (0.52-0.83)	**0.0005**
Extra-hepatic disease prior to treatment	No	6.9 (6.0-7.9)	0.1827		
	Yes	5.2 (3.3-8.0)		1.29 (0.89-1.88)	0.1827
Portal vein thrombosis	Lobar	5.5 (3.5-6.5)	0.1533	1.45 (1.00-2.13)	0.0530
	Main	4.7 (3.0-8.0)		1.41 (0.86-2.31)	0.1716
	Patent	7.4 (6.3-8.8)			
	Segmental	6.2 (5.4-8.1)		1.11 (0.84-1.46)	0.4731
BCLC stage	A	12.5 (7.7-21.1)	**0.0013**		
	B	6.6 (5.5-7.9)		1.68 (1.13-2.50)	**0.0105**
	C	5.9 (5.3-7.2)		2.04 (1.41-2.94)	**0.0001**
	D	11.2 (4.0-12.7)		1.44 (0.51-4.08)	0.4892
Total bilirubin (mg/dl)	≤1.5	7.0 (6.0-7.9)	0.1432		
	>1.5	4.6 (3.3-8.4)		1.32 (0.91-1.91)	0.1444
Prior procedures	No	6.6 (5.7-7.7)	0.5326		
	Yes	7.2 (5.8-9.2)		0.93 (0.75-1.16)	0.5349
Surgery	No	6.6 (5.8-7.7)	0.3336		
	Yes	7.6 (4.7-12.0)		0.87 (0.65-1.16)	0.3347
Ablation	No	6.3 (5.7-7.3)	**0.0312**		
	Yes	10.0 (6.7-16.1)		0.69 (0.50-0.97)	**0.0323**
TACE	No	7.0 (6.0-7.7)	0.1999		
	Yes	5.9 (3.7-8.7)		1.18 (0.92-1.52)	0.1999
Abdominal radiotherapy	No	6.7 (5.9-7.6)	0.1239		
	Yes	11.6 (3.0-ND)		0.47 (0.18-1.26)	0.1330

Other prior embolotherapies	No	6.8 (6.0-7.7)	0.4387		
	Yes	4.3 (3.3-13.4)		1.25 (0.72-2.17)	0.4360
Prior chemotherapy	No	7.0 (6.0-8.0)	0.1905		
	Yes	5.7 (3.7-6.8)		1.27 (0.89-1.82)	0.1908
Treatment intention	Ablation	13.6 (7.7-33.6)	**<0.0001**	0.42 (0.22-0.79)	**0.0075**
	Bridge to surgery or transplant	7.6 (4.4-.)		0.49 (0.29-0.84)	**0.0088**
	Down-sizing/down-staging	9.7 (7.2-14.0)		0.59 (0.46-0.76)	**<0.0001**
	Palliative	5.6 (4.7-6.3)			
Treatment intention	Curative	10.4 (7.6-14.0)	**<0.0001**	0.56 (0.45-0.71)	**<0.0001**
	Palliative	5.6 (4.7-6.3)			
Dose methodology	BSA/mBSA	6.3 (5.5-7.3)	**0.0308**		
	Partition model	7.4 (6.0-10.0)		0.78 (0.63-0.98)	**0.0314**
ALBI grade	1	7.9 (6.5-10.4)	**0.0162**		
	2	6.0 (5.5-7.2)		1.22 (0.96-1.55)	0.1057
	3	4.6 (2.6-8.6)		2.20 (1.23-3.92)	**0.0078**

Levels of significance: $p < 0.05$ (Log-rank test [Mantel-Haenszel version]).
ALBI: Albumin Bilirubin; BCLC Barcelona Clinic Liver Cancer; BSA: Body Surface Area; ECOG: Eastern Cooperative Oncology Group; NASH: Non-alcoholic steatohepatitis; TACE: Transcatheter Arterial Chemoembolization; TARE: Transarterial Radioembolization.

Table S4. Multivariable analysis for progression-free survival

Variable	Threshold	HR (95% CI)	p value
BCLC (vs A)	B	1.58 (1.06-2.34)	**0.025**
	C	1.99 (1.38-2.86)	**0.0002**
	D	1.34 (0.47-3.78)	0.5851
Cirrhosis (vs No)	Yes	1.31 (1.03-1.67)	**0.0312**
Tumour location (vs bilobar)	Left	0.78 (0.54-1.12)	0.1713
	Right	0.73 (0.57-0.92)	**0.0092**
Treatment intention (vs palliative)	Curative	0.55 (0.43-0.70)	**<.0001**

Levels of significance: p <0.05 (Cox proportional-hazards model). The proportional hazard function of the Cox model was verified. The following variables were considered in the multivariable model: Barcelona Clinic Liver Cancer; Eastern Cooperative Oncology Group; cirrhosis; ascites; tumour burden (nodules); location of tumour; extra-hepatic disease prior to transarterial radioembolization; prior ablation; prior chemotherapy; treatment intention; dose methodology; albumin-bilirubin grade. ALBI: Albumin-Bilirubin; BCLC Barcelona Clinic Liver Cancer; BSA: Body Surface Area; ECOG: Eastern Cooperative Oncology Group; TARE: Transarterial Radioembolization.

Table S5. Multivariable analysis for hepatic progression free survival

Variable	Threshold	HR (95% CI)	p value
ECOG (vs 0)	1	1.32 (1.03-1.70)	**0.0274**
	2+3	1.22 (0.77-1.92)	0.4011
Cirrhosis (vs No)	Yes	1.43 (1.11-1.85)	**0.0060**
Location of tumour (vs Bilobar)	Left	0.80 (0.56-1.16)	0.2368
	Right	0.65 (0.51-0.83)	**0.0005**
Portal vein thrombosis (vs Patent)	Lobar	1.71 (1.15-2.56)	**0.0086**
	Main	1.53 (0.92-2.55)	0.1055
	Segmental	1.13 (0.85-1.50)	0.3865
Ablation (vs No)	Yes	0.78 (0.55-1.10)	0.1585
Abdominal radiotherapy (vs No)	Yes	0.50 (0.18-1.38)	0.1817
Treatment intention Curative (vs Palliative)	Curative	0.59 (0.46-0.75)	**<0.0001**

Levels of significance: $p < 0.05$ (Cox proportional-hazards model). The proportional hazard function of the Cox model was verified. The following variables were considered in the multivariable model: Barcelona Clinic Liver Cancer; Eastern Cooperative Oncology Group; cirrhosis; ascites; tumour burden (nodules); location of tumour; extra-hepatic disease prior to transarterial radioembolization; portal vein thrombosis; total bilirubin (mg/dl); prior ablation; prior transcatheter arterial chemoembolization; prior chemotherapy; treatment intention; dose methodology; albumin-bilirubin grade. ALBI: Albumin-Bilirubin; BCLC Barcelona Clinic Liver Cancer; BSA: Body Surface Area; ECOG: Eastern Cooperative Oncology Group; TARE: Transarterial Radioembolization.

Table S6. Propensity score matching

**Variable**	**Type**	**BSA/mBSA (n=213)**	**Compartment Model (n=160)**	**All (n=373)***	**P value**
Cirrhosis	No	87 (40.8%)	26 (16.3%)	113 (30.3%)	**<.0001**
	Yes	126 (59.2%)	134 (83.8%)	260 (69.7%)	
Ascites	No	182 (85.4%)	141 (88.1%)	323 (86.6%)	0.4523
	Yes	31 (14.6%)	19 (11.9%)	50 (13.4%)	
Number of nodules	1	40 (18.8%)	80 (50.0%)	120 (32.2%)	**<.0001**
	2-5	71 (33.3%)	51 (31.9%)	122 (32.7%)	
	>5	52 (24.4%)	10 (6.3%)	62 (16.6%)	
	Uncountable	50 (23.5%)	19 (11.9%)	69 (18.5%)	
Location of tumour	Bilobar	106 (49.8%)	28 (17.5%)	134 (35.9%)	**<.0001**
	Left	22 (10.3%)	22 (13.8%)	44 (11.8%)	
	Right	85 (39.9%)	110 (68.8%)	195 (52.3%)	
Portal vein thrombosis	Patent	159 (74.6%)	95 (59.4%)	254 (68.1%)	**0.0163**
	Main	9 (4.2%)	8 (5.0%)	17 (4.6%)	
	Lobar	13 (6.1%)	17 (10.6%)	30 (8.0%)	
	Segmental	32 (15.0%)	40 (25.0%)	72 (19.3%)	
ALBI grade	1	91 (42.7%)	48 (30.0%)	139 (37.3%)	**0.0421**
	2	114 (53.5%)	105 (65.6%)	219 (58.7%)	
	3	8 (3.8%)	7 (4.4%)	15 (4.0%)	

Levels of significance: p <0.05 (Log-rank test [Mantel-Haenszel version]).
*Due to the presence of missing values for the ALBI grade, a total of 373 patients was considered for the analysis. ALBI: Albumin-Bilirubin; BSA: Body Surface Area;

Table S7. Comparing baseline characteristics

Variable	Type	BSA/mBSA	Partition model	All	P value
Age (year)	N :	245	177	422	0.1142
	Mean ± SD :	67.7 ± 10.9	66.0 ± 10.3	67.0 ± 10.7	
	(Min;Max) :	(22.0 ; 92.0)	(36.0 ; 91.0)	(22.0 ; 92.0)	
	Median [IQR] :	68.0 [62.0;74.0]	67.0 [59.0;75.0]	68.0 [60.0;74.0]	
Age (year)	<68	120 (49.0%)	90 (50.8%)	210 (49.8%)	0.7049
	≥68	125 (51.0%)	87 (49.2%)	212 (50.2%)	
Gender	Female	40 (16.3%)	34 (19.2%)	74 (17.5%)	0.7601
	Male	201 (82.0%)	140 (79.1%)	341 (80.8%)	
	Unknown	4 (1.6%)	3 (1.7%)	7 (1.7%)	
ECOG	0	123 (50.2%)	137 (77.4%)	260 (61.6%)	**<.0001**
	1	95 (38.8%)	36 (20.3%)	131 (31.0%)	
	2+3	27 (11.0%)	4 (2.3%)	31 (7.3%)	
Cirrhosis	No	94 (38.4%)	29 (16.4%)	123 (29.1%)	**<.0001**
	Yes	151 (61.6%)	148 (83.6%)	299 (70.9%)	
Cause of cirrhosis	Alcohol	51 (33.8%)	24 (16.2%)	75 (25.1%)	**<.0001**
	Hepatitis-B	17 (11.3%)	33 (22.3%)	50 (16.7%)	
	Hepatitis-C	36 (23.8%)	64 (43.2%)	100 (33.4%)	
	NASH	29 (19.2%)	9 (6.1%)	38 (12.7%)	
	Other	18 (11.9%)	18 (12.2%)	36 (12.0%)	
Ascites	No	205 (83.7%)	156 (88.1%)	361 (85.5%)	0.1983
	Yes	40 (16.3%)	21 (11.9%)	61 (14.5%)	
Number of nodules	1	47 (19.2%)	89 (50.3%)	136 (32.2%)	**<.0001**
	2-5	81 (33.1%)	57 (32.2%)	138 (32.7%)	
	>5	61 (24.9%)	11 (6.2%)	72 (17.1%)	
	Uncountable	56 (22.9%)	20 (11.3%)	76 (18.0%)	
Location of tumor	Bilobar	120 (49.0%)	30 (16.9%)	150 (35.5%)	**<.0001**
	Left	27 (11.0%)	24 (13.6%)	51 (12.1%)	
	Right	98 (40.0%)	123 (69.5%)	221 (52.4%)	
Extra-hepatic disease prior to treatment	No	219 (89.4%)	167 (94.4%)	386 (91.5%)	0.0717
	Yes	26 (10.6%)	10 (5.6%)	36 (8.5%)	
Portal vein thrombosis	Patent	178 (72.7%)	106 (59.9%)	284 (67.3%)	**0.0351**
	Main	11 (4.5%)	8 (4.5%)	19 (4.5%)	
	Lobar	19 (7.8%)	19 (10.7%)	38 (9.0%)	
	Segmental	37 (15.1%)	44 (24.9%)	81 (19.2%)	
BCLC stage	A	23 (9.4%)	36 (20.3%)	59 (14.0%)	**0.0011**
	B	140 (57.1%)	77 (43.5%)	217 (51.4%)	
	C	78 (31.8%)	64 (36.2%)	142 (33.6%)	
	D	4 (1.6%)	0 (0.0%)	4 (0.9%)	
Total bilirubin (mg/dl)	N :	243	176	419	**0.0148**
	Mean ± SD :	0.9 ± 0.5	1.0 ± 0.5	0.9 ± 0.5	
	(Min;Max) :	(0.1 ; 2.7)	(0.2 ; 3.0)	(0.1 ; 3.0)	
	Median [IQR] :	0.8 [0.5;1.1]	0.9 [0.6;1.2]	0.8 [0.5;1.2]	
Total bilirubin (mg/dl)	≤1.5	224 (92.2%)	153 (86.9%)	377 (90.0%)	0.0774
	>1.5	19 (7.8%)	23 (13.1%)	42 (10.0%)	
Albumin (g/dL)	N :	213	161	374	0.3003
	Mean ± SD :	3.7 ± 0.6	3.7 ± 0.5	3.7 ± 0.5	
	(Min;Max) :	(1.8 ; 5.1)	(2.0 ; 4.7)	(1.8 ; 5.1)	
	Median [IQR] :	3.8 [3.4;4.1]	3.7 [3.4;4.0]	3.7 [3.4;4.1]	

Bilirubin (µmol/L)	N :	243	176	419	**0.0117**
	Mean ± SD :	14.6 ± 7.9	16.6 ± 8.6	15.4 ± 8.3	
	(Min;Max) :	(2.0 ; 46.0)	(3.4 ; 52.0)	(2.0 ; 52.0)	
	Median [IQR] :	13.0 [8.6;18.8]	15.1 [10.1;21.0]	14.0 [9.1;20.0]	
ALBI grade	DM	32 (13.1%)	17 (9.6%)	49 (11.6%)	**0.0421**
	A1	91 (37.1%)	48 (27.1%)	139 (32.9%)	
	A2	114 (46.5%)	105 (59.3%)	219 (51.9%)	
	A3	8 (3.3%)	7 (4.0%)	15 (3.6%)	
Prior locoregional procedures	No	133 (54.3%)	100 (56.5%)	233 (55.2%)	0.6521
	Yes	112 (45.7%)	77 (43.5%)	189 (44.8%)	
Prior surgery	No	193 (78.8%)	157 (88.7%)	350 (82.9%)	**0.0075**
	Yes	52 (21.2%)	20 (11.3%)	72 (17.1%)	
Type of prior surgery	Liver surgery	50 (20.4%)	20 (11.3%)	70 (16.6%)	0.1112
	Liver transplant	2 (0.8%)	0 (0.0%)	2 (0.5%)	
Prior ablation	No	213 (86.9%)	149 (84.2%)	362 (85.8%)	0.4234
	Yes	32 (13.1%)	28 (15.8%)	60 (14.2%)	
Prior TACE	No	196 (80.0%)	131 (74.0%)	327 (77.5%)	0.1461
	Yes	49 (20.0%)	46 (26.0%)	95 (22.5%)	
Prior TACE	Conventional TACE	27 (11.0%)	38 (21.5%)	65 (15.4%)	**0.0099**
	Drug-Eluting TACE	19 (7.8%)	7 (4.0%)	26 (6.2%)	
	Other	3 (1.2%)	1 (0.6%)	4 (0.9%)	
Prior abdominal radiotherapy	No	239 (97.6%)	176 (99.4%)	415 (98.3%)	0.2469
	Yes	6 (2.4%)	1 (0.6%)	7 (1.7%)	
Other prior embolotherapies	No	242 (98.8%)	165 (93.2%)	407 (96.4%)	**0.0029**
	Yes	3 (1.2%)	12 (6.8%)	15 (3.6%)	
Prior chemotherapy	No	216 (88.2%)	165 (93.2%)	381 (90.3%)	0.0835
	Yes	29 (11.8%)	12 (6.8%)	41 (9.7%)	
Chemotherapy	Missing	2 (6.9%)	0 (0.0%)	2 (4.9%)	0.0891
	Other	0 (0.0%)	2 (16.7%)	2 (4.9%)	
	Sorafenib	27 (93.1%)	10 (83.3%)	37 (90.2%)	
Treatment intention	Ablation	3 (1.2%)	14 (7.9%)	17 (4.0%)	**<.0001**
	Bridge to surgery or transplant	17 (6.9%)	9 (5.1%)	26 (6.2%)	
	Down-sizing/down-staging	60 (24.5%)	77 (43.5%)	137 (32.5%)	
	Palliative	165 (67.3%)	77 (43.5%)	242 (57.3%)	
Treatment intention	Curative	80 (32.7%)	100 (56.5%)	180 (42.7%)	**<.0001**
	Palliative	165 (67.3%)	77 (43.5%)	242 (57.3%)	
Prescribed activity whole liver treatment (GBq)	N :	71	114	185	**0.0102**
	Mean ± SD :	1.5 ± 0.7	1.3 ± 0.6	1.4 ± 0.6	
	(Min;Max) :	(0.7 ; 5.0)	(0.2 ; 3.0)	(0.2 ; 5.0)	
	Median [IQR] :	1.5 [1.2;1.8]	1.3 [0.8;1.7]	1.4 [1.0;1.8]	
Prescribed activity whole liver treatment (GBq)	≤0.993	12 (4.9%)	35 (19.8%)	47 (11.1%)	0.1048
	0.993-1.4	19 (7.8%)	33 (18.6%)	52 (12.3%)	
	1.4-1.76	20 (8.2%)	20 (11.3%)	40 (9.5%)	
	>1.76	20 (8.2%)	26 (14.7%)	46 (10.9%)	
	N :	174	63	237	**0.0089**

Prescribed activity left lobe treatment (GBq)	Mean ± SD :	0.4 ± 0.5	0.3 ± 0.6	0.4 ± 0.5	
	(Min;Max) :	(0.0 ; 2.0)	(0.0 ; 3.0)	(0.0 ; 3.0)	
	Median [IQR] :	0.3 [0.0;0.7]	0.0 [0.0;0.4]	0.0 [0.0;0.7]	
Prescribed activity left lobe treatment (GBq)	≤0	85 (34.7%)	47 (26.6%)	132 (31.3%)	**0.0002**
	0-0.67	44 (18.0%)	3 (1.7%)	47 (11.1%)	
	>0.67	45 (18.4%)	13 (7.3%)	58 (13.7%)	
Prescribed activity right lobe treatment (GBq)	N :	174	63	237	0.4999
	Mean ± SD :	1.1 ± 0.7	1.0 ± 0.9	1.1 ± 0.8	
	(Min;Max) :	(0.0 ; 5.5)	(0.0 ; 3.5)	(0.0 ; 5.5)	
	Median [IQR] :	1.1 [0.8;1.4]	1.0 [0.4;1.5]	1.1 [0.7;1.4]	
Prescribed activity right lobe treatment (GBq)	≤0.666	36 (14.7%)	25 (14.1%)	61 (14.5%)	**0.0031**
	0.666-1.1	50 (20.4%)	10 (5.6%)	60 (14.2%)	
	1.1-1.43	48 (19.6%)	9 (5.1%)	57 (13.5%)	
	>1.43	40 (16.3%)	19 (10.7%)	59 (14.0%)	
APRI	N :	203	172	375	0.7277
	Mean ± SD :	1.1 ± 1.2	1.2 ± 1.4	1.1 ± 1.3	
	(Min;Max) :	(0.1 ; 10.1)	(0.1 ; 14.7)	(0.1 ; 14.7)	
	Median [IQR] :	0.8 [0.4;1.4]	0.8 [0.4;1.4]	0.8 [0.4;1.4]	
APRI	Missing	42 (17.1%)	5 (2.8%)	47 (11.1%)	0.8163
	≤0.40	44 (18.0%)	39 (22.0%)	83 (19.7%)	
	>0.40	159 (64.9%)	133 (75.1%)	292 (69.2%)	

Levels of significance: p <0.05 (Log-rank test [Mantel-Haenszel version]).
ALBI: Albumin-Bilirubin; APRI: Aspartate aminotransferase-to-platelet ratio index; BCLC Barcelona Clinic Liver Cancer; (m)BSA: (modified) Body Surface Area; ECOG: Eastern Cooperative Oncology Group; GBQ: Giga-becquerel; INR: International Normalized Ratio; IQR: Interquartile Range; NASH: Non-alcoholic steatohepatitis; TACE: Transcatheter Arterial Chemoembolization;

Table S8. The effect of centre expertise in survival outcomes between the BSA/mBSA and the partition model cohorts.

The hypothesis is that centres that perform partition model dosimetry are generally better-performing hospitals, because partition model dosimetry requires a certain level of expertise and infrastructure dedicated to TARE. If this hypothesis would be true, we would expect better OS/PFS/hPFS outcomes of the BSA/mBSA patients that are treated in those hospitals.

In the below table we compared the survival outcomes of the following three groups: 1) partition model dosimetry patients; 2) dose calculation with BSA/mBSA for patients that were treated in sites that also performed partition model dosimetry; and 3) dose calculation with BSA/mBSA for patients that were treated in sites that did not perform partition model dosimetry.

Outcome	Group	No. of patients	Median (95% CI)	p value	HR (95% CI)	p value HR
**OS**	1	177	23.4 (18.3-38.9)	<0.0001	0.50 (0.38-0.67)	0
	2	73	15.3 (8.7-20.3)		0.84 (0.60-1.18)	0.3111
	3	172	13.3 (11.5-15.0)			
**PFS**	1	177	7.4 (5.9-10.0)	0.005	0.68 (0.54-0.87)	0.0016
	2	73	5.5 (3.5-6.9)		0.93 (0.69-1.25)	0.6247
	3	171	5.7 (5.1-7.0)			
**hPFS**	1	177	7.4 (6.0-10.0)	0.0955	0.78 (0.61-0.99)	0.0397
	2	73	5.9 (4.0-7.7)		0.97 (0.72-1.32)	0.855
	3	172	6.8 (5.6-7.7)			

Levels of significance: p <0.05 (Log-rank test [Mantel-Haenszel version]).
1. Patients with partition model
2. Patients with BSA/mBSA in sites that also performed partition model
3. Patients with BSA/mBSA in sites that did not perform partition model

This table shows that BSA group 2 did not benefit from being treated at a site that also performed partition model dosimetry. In fact, when we compare only the 2 groups with BSA/mBSA we see that there are no significant differences between the outcomes of the groups, suggesting that dose calculation with BSA leads to similar outcomes, regardless of the expertise and infrastructure of the centre.

**Outcome**	**HR (95% CI)**	**p value HR**
OS	0.85 (0.61-1.18)	0.3326
PFS	0.94 (0.69-1.27)	0.6684
hPFS	0.98 (0.72-1.33)	0.9

Levels of significance: p <0.05 (Log-rank test [Mantel-Haenszel version]).

Table S9. Toxicities.

A: Short-term safety data

Category	Subcategory	HCC (n=422)
Longest hospital stay	n[a]	323
	24 to <36 hours	57 (13.5%)
	36 to <48 hours	42 (10.0%)
	48 to <72 hours	186 (44.1%)
	> or = 72 hours	38 (9.0%)
**Severe day of treatment complications**		
Severe abdominal pain	Yes	3 (0.7%)
Severe abdominal pain	Yes: Grade 3	3 (0.7%)
	Yes: Grade 4	
Severe vomiting	Yes	2 (0.5%)
Severe vomiting	Yes: Grade 3	2 (0.5%)
Vascular	Yes	3 (0.7%)
Vascular	Yes: Major	1 (0.2%)
	Yes: Minor	2 (0.5%)
Severe other	Yes	4 (0.9%)
**Time till death**		
n[a]	422	n[a]
Death within 30 days	3 (0.7%)	Within 30 days
Death between 31 and 60 days	7 (1.7%)	Between 31 and 60 days
Death between 61 and 90 days	15 (3.6%)	Between 61 and 90 days
Lost to follow-up within 90 days	47 (11.1%)	Lost to follow-up within 90 days

B: Adverse events

Category	Subcategory	HCC (n=422)
Patients with adverse events	Number of patients with at least one adverse event	155 (36.7%)
Adverse events (all)	Abdominal pain	60 (14.2%)
	Fatigue	65 (15.4%)
	Fever	25 (5.9%)
	Nausea	37 (8.8%)
	Vomiting	22 (5.2%)
	Gastrointestinal ulceration	3 (0.7%)
	Gastritis	3 (0.7%)
	Radiation cholecystitis	
	Radiation pancreatitis	
	Radioembolisation-induced liver disease	6 (1.4%)
	Other	90 (21.3%)
Adverse events grade 3-4	Abdominal pain	9 (2.1%)
	Fatigue	6 (1.4%)
	Fever	2 (0.5%)
	Nausea	3 (0.7%)
	Vomiting	2 (0.5%)
	Gastrointestinal ulceration	1 (0.2%)
	Gastritis	

	Radiation cholecystitis	
	Radioembolisation-induced liver disease	1 (0.2%)
	Other	14 (3.3%)
Adverse events grade 5	Radioembolisation-induced liver disease	2 (0.5%)
	Other	3 (0.7%)
All adverse events	Number of adverse events	646
	Person years	197.05
	Incidence (95% CI)	3.28 (3.278 - 3.279)
Adverse events of special interest	Number of adverse events	399
	Person years	126.89
	Incidence (95% CI)	3.14 (3.144 - 3.145)
Time without toxicity	Time without toxicity (95% CI)	13.8 months (11.1 - 24.0)

C: Abnormal lab values

**Category**	**Subcategory**	**HCC (n=422)**
Patients with abnormal lab values	No. of patients with at least one abnormal lab	254 (60.2%)
Abnormal lab values	Hypoalbuminemia	126 (29.9%)
	Hyperbilirubinemia	124 (29.4%)
	ALT increased	109 (25.8%)
	AST increased	154 (36.5%)
	INR increased	89 (21.1%)
	Neutrophil count decreased	16 (3.8%)
	Platelet count decreased	137 (32.5%)
	Other	101 (23.9%)
ALT: Alanine Aminotransferase; AST: Aspartate Transaminase; INR: International Normalized Ratio		
